# An Easy Route to Aziridine Ketones and Carbinols

**DOI:** 10.3390/ijms222313145

**Published:** 2021-12-05

**Authors:** Boriss Strumfs, Jekaterina Hermane, Sergey Belyakov, Artjoms Sobolevs, Kirils Velikijs, Romans Uljanovs, Peteris Trapencieris, Ilze Strumfa

**Affiliations:** 1 Department of Pathology, Faculty of Medicine, Riga Stradins University, 16 Dzirciema Street, LV-1007 Riga, Latvia; Boriss.Strumfs@rsu.lv (B.S.); Artjoms.Sobolevs@rsu.lv (A.S.); Kirils.Velikijs@rsu.lv (K.V.); Romans.Uljanovs@rsu.lv (R.U.); 2 Latvian Institute of Organic Synthesis, 21 Aizkraukles Street, LV-1006 Riga, Latvia; h.katrin@gmail.com (J.H.); serg@osi.lv (S.B.); peteris@osi.lv (P.T.)

**Keywords:** aziridines, organolithium reagents, tertiary amides, regioselectivity, ketones, carbinols

## Abstract

*N,N*-Dimethylaziridine-2-carboxamides react with organolithium reagents yielding 2-aziridinylketones. The reaction with one equivalent of organolithium compound is selective to amide carbonyl at a low (−78 °C) temperature. These ketones, in reaction with organolithium reagents, give symmetrical and unsymmetrical aziridinyl carbinols. The usage of excess phenyllithium may serve as a special N-Boc-protecting group cleavage method for acid-sensitive substrates.

## 1. Introduction

The unusual bond angles and shorter than normal sp^3^ carbon bond in aziridines, combined with the strain in the three-membered ring and the electronegativity of the nitrogen atom, make these molecules interesting and powerful building blocks. Aziridines are excellent substrates for the selective synthesis of unnatural amino acids, amino alcohols and different heterocycles of biomedical interest [1,2,3,4,5,6,7,8]. For this reason, easy access to 2-aziridinyl ketones and corresponding carbinols is highly necessary.

Reactions of carboxylic acid derivatives with C-nucleophiles through stable tetrahedral intermediates are common. This is an important method for the synthesis of carbonyl compounds, including ketones and carbinols [9]. It is also known that C-nucleophiles can react with aziridine-2-carboxylic acid derivatives in two ways: either by the opening of the aziridine ring or at a carbonyl C-atom on the side chain. Appropriate substrates for the latter reaction are represented by aziridine-2-carboxylic esters and lithium aziridine-2-carboxylates [10] or Weinreb amides [11,12,13]. The first equivalent of the organolithium nucleophile gives a ketone, and the second forms a carbinol [9].

As mentioned above, the corresponding Weinreb amides are useful substrates for the synthesis of 2-aziridinyl ketones and carbinols [11,12,13,14,15]. These activated substrates are successfully applied to obtain NH-aziridines [11], aziridinyl ketone and carbinol intermediates in the synthesis of optically active protected aminoalcohols [12] and 3-amino-2.3-dihydrobenzopyran-4-ones [13]. Weinreb amides in the series of aziridine-2-carboxylic acid can be obtained from the glycidic ester in a four-step procedure [11,13]. This route includes: (a) saponification of ester, (b) DCC coupling with *N*,*O*-dimethylhydroxylamine to form Weinreb amides, (c) an oxirane ring opening with an azide and (d) a PPh_3_-mediated ring closure to form an aziridine ring. Alternative and much shorter routes to Weinreb amides are aminohydroxylation of cinnamamides followed by the closure of the aziridine ring through the O-mesylate–base procedure [14] and aziridination of α,β-unsaturated Weinreb amides with PhI=NTs [15]. The only direct approach to one specific aziridine Weinreb amide applied to the ester–menthyl N-(α-methylbenzyl)-aziridine-2-carboxylate by trimethyl-aluminium-mediated aminolysis was described by Lee [12]. Thus, the Weinreb amide approach in the aziridine series is a complicated, substrate-specific and unpredictable pathway of synthesis.

Tertiary amides represent useful alternatives to Weinreb amides in carbonyl compound synthesis. Recently, the reaction of activated mono-Boc and bis-Boc amides with Grignard reagents led to high yields in the corresponding aromatic ketones [16]. Some examples of successful utilization of non-activated tertiary dimethylamides in aldehyde and ketone synthesis are also found in the literature [17,18,19]. Thus, hydride addition to dimethylamides in the presence of alkaline metal iodides forms aldehydes [17], but dimethylamide substrates with organosamariums [18] and organolithiums [19] yield the corresponding ketones. However, these methods from dimethylamides have not been applied in the aziridine series. Therefore, we describe an alternative and simple approach to 2-aziridinylketones and corresponding carbinols based on the usage of non-activated robust *N,N*-dimethyl aziridine-2-carboxamides. The carboxamides were obtained from commercially available methyl aziridine-2-carboxylate as the starting material in the reaction with commercially available organolithiums. This protocol may exclude the multistep synthesis of aziridine Weinreb amide substrates.

## 2. Results and Discussion

**Scheme 1:**
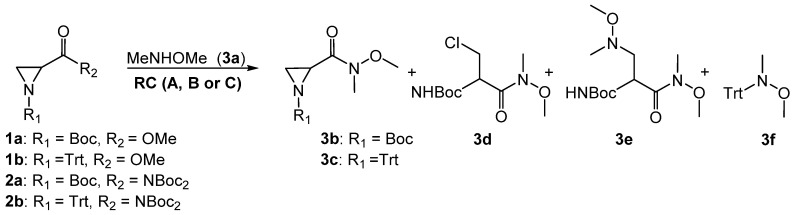
Synthesis of hydroxylamines **3b–f** from esters **1a,b** and carbamates **2a,b** in reaction with hydroxylamine **3a**.

Our initial attempts to obtain aziridine Weinreb amides directly from N-Boc protected ester **1a** using AlMe_3_-mediated aminolysis [12] (Scheme 1, Table 1) produced open-chain ring-opening by-products **3d** and **3e** (entries 1 and 2) and only traces of Weinreb amide **3b** in the case of the modified aminolysis procedure (entry 3). In the case of starting with ester **1b**, only an aziridine degradation by-product, namely, hydroxylamine **3f,** was isolated (entries 4 and 5). With 3 eq. of hydroxylamine **3a**, the starting material **1b** disappeared, and only the by-product was isolated in a 62% yield.

In a recent alternative approach to the expected Weinreb amides, bis-Boc activation was used [16]. With N-Boc aziridine **2a**, it formed the desired Boc-protected Weinreb amide **3b** in a moderate (31%) yield (entry 6). Unfortunately, N-Trt aziridine **2b** yielded only traces of product **3c** after 120 h reflux (entries 6 and 7). After all these failed experiments, we concluded that an alternative method with more convenient and practical starting materials for aziridinyl ketone synthesis was necessary.

**Scheme 2:**
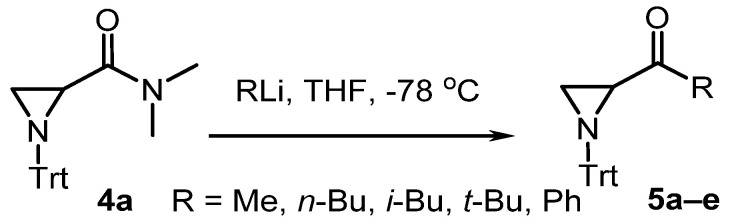
Synthesis of ketones **5a–e** from amide **4a**.

From our previous work [8], we knew that dimethyl amides **4a** (Scheme 2) and **4b** (Scheme 4) were more readily available in comparison with Weinreb amides through direct aminolysis of corresponding methyl esters. In the synthesis of 2-aziridinylketones from *N*-protected *N,N*-dimethyl aziridine-2-carboxamides **4a,b** as substrates, we have demonstrated comparable results in chemical yields and regioselectivity with the Weinreb amides described in the literature [11,12,13].

We have used organolithium reagents as carbon nucleophiles to obtain the desired 2-aziridinylketones **5** and **7** from N-tritylated and N-Boc substrates **4a** and **4b**. Preliminary tests with N-benzyloxycarbonyl analogues of the amide **4** in the reactions with organolithiums showed inappropriate results, most likely because of the side reactions on the active benzyl CH_2_ fragment. Thus, we started with the use of protected N-tritylated amide **4a** (Scheme 2, Table 2). According to our observations, the sterically less hindered compound, ketone **5a** (Table 2, entries 1–3), was obtained in better yields, but bulkier alkyllithiums gave ketones **5b**–**d** (Table 2, entries 4–13) in lower yields. Longer reaction times and an excess of organolithium reagents did not increase the yields of ketones in the reactions with alkyllithiums. Prolonged control experiments (Table 2, entries 3, 6, 8, 13) demonstrated that prolonged reaction time led to a lowering of product **5d** yields because of side reactions. An excess of the organolithium compound did not give side products, because dimethyl amide **4a** and the organolithium reagent initially formed an intermediate adduct [9], which liberated ketones **5** and **7** only in the hydrolysis step. Compared to alkyllithium compounds, phenyllithium reacted more slowly with amide **4a**. The prolonging of the reaction time from 1 h to 12 h increased the yield of ketone **5e** from 51% to 79% using 1 eq of phenyllithium (Table 2, entries 14 and 15). Using an excess of phenyllithium did not improve the product 5e yield (Table 2, entries 16–18). At the same time, a nucleophilic aziridine ring opening was not observed. We have found that under the explored reaction conditions (−78 °C, quenching of reaction mixture without warming), the electrophilic dimethylamide group is more reactive than an electrophilic aziridine ring toward the organolithium reagents used as carbon-nucleophiles.

**Scheme 3:**
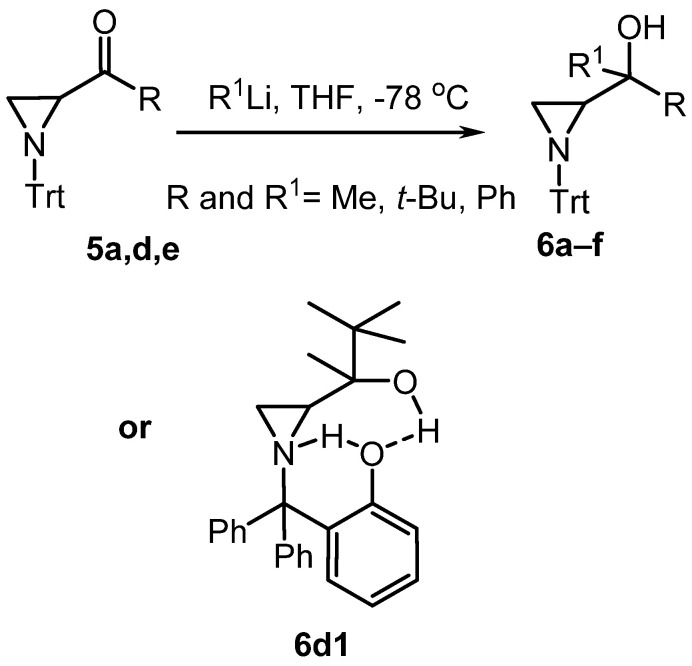
Synthesis of carbinols **6a–f** and phenol **6d1** from ketones **5a,d,e**.

**Figure 1:**
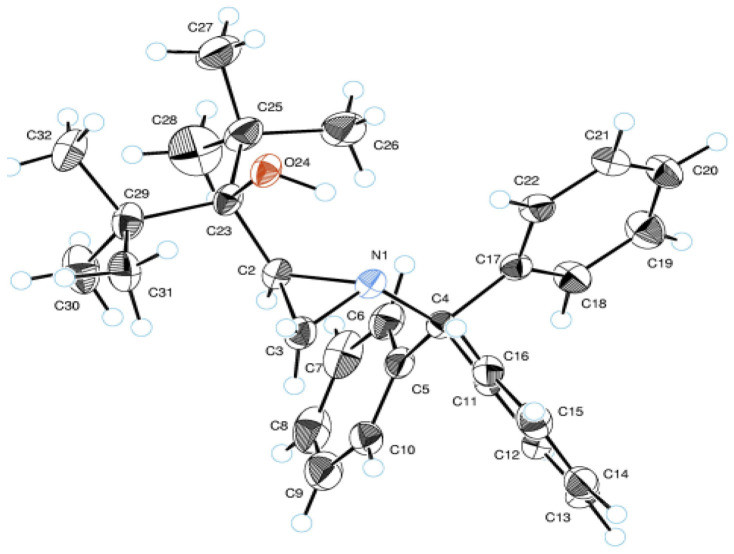
Crystal X-ray structure of carbinol **6c**.

When isolated ketones **5** interacted with the organolithium reagent (R^1^Li) once more, an addition to the substrate carbonyl group was observed. The corresponding symmetrical or unsymmetrical carbinols **6a–f** were isolated after the quenching of reaction mixtures (Scheme 3, Table 3). The reaction of substrate **5a** with methyllithium showed moderate yields of carbinol **6a** (Table 3, entries 1–4). The aromatic group (PhLi as a reagent, product **6b**) and sterically hindered tert-butyl group (*tert*-BuLi as a reagent, product **6c**) stimulated the formation of carbinols (Table 3, entries 5–12). Moreover, in reactions of ketones **5d**,**e** with PhLi and tert-BuLi, longer reaction times increased yields of products **6b**,**c** (Table 3, entries 6, 8, 11). The structure of carbinol **6c** was confirmed by X-ray analysis (Figure 1).

**Figure 2:**
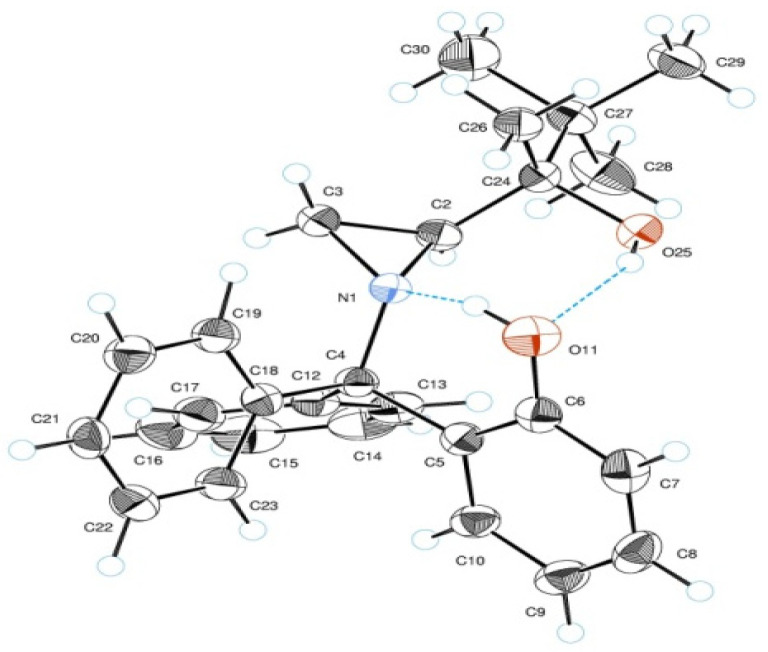
Crystal X-ray structure of carbinol **6d1**.

Ketone **5d** reacted with MeLi unexpectedly in the N-trityl group, and instead of carbinol **6d,** the hydroxylation product phenol **6d1** was isolated in a 52% yield (Table 3, entry 14). On the other hand, from ketone **5a** in reaction with tert-BuLi (Table 3, entry 13), a carbinol **6d** and a phenol **6d1** as a ~1:1 mixture were obtained. Formation of the phenol **6d1** may be a result of ortho-lithiation of the phenyl group in the substrate trityl moiety and will be a matter for further investigations. The structure of phenol **6d1** was confirmed by X-ray analysis (Figure 2).

**Scheme 4:**
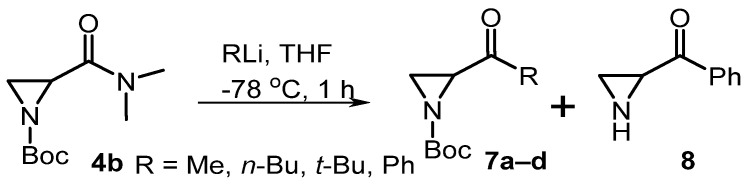
Synthesis of ketones **7a–d** and **8** from dimethylamide **4b**.

Reactions of organolithiums with both electron-rich Trt (substrates **4a**, Scheme 2, Table 2) and electron-deficient Boc (substrate **4b**, Scheme 4, Table 4) N-substituents containing dimethylamides were found to be regioselective (Table 4, entries 1–5). No side reactions at the aziridine ring system were observed. However, in the reaction of amide **4b** with a four-fold excess of phenyllithium beside the expected ketone **7d**, N-deprotected aziridine **8** was observed (Table 4, entry 6). We concluded that NH-aziridine **8** was formed by deprotection of the Boc group in basic conditions. To complete the N-Boc deprotection reaction, we have modified reaction conditions (entry 7) by warming up the reaction mixture before quenching. As a result, we have isolated NH-aziridine **8** as a sole product in a 66% yield.

The structure of NH-aziridine **8** [20] was confirmed chemically by the performed reaction with Boc-anhydride, and the expected ketone **7d** was isolated in a 78% yield. Longer reaction times up to 12 h after warming the reaction mixture to room temperature with an excess of organolithium reagent only gave aziridine cleavage products as inseparable mixtures. Similarly, Weinreb amide **3b** in the reaction with PhLi also resulted in inseparable reaction mixtures. Thus, we have concluded that electron-deficient aziridine **4b**, in the reaction with one equivalent of organolithium reagent, is selective to amide carbonyl only at a low (−78 °C) temperature.

**Scheme 5:**
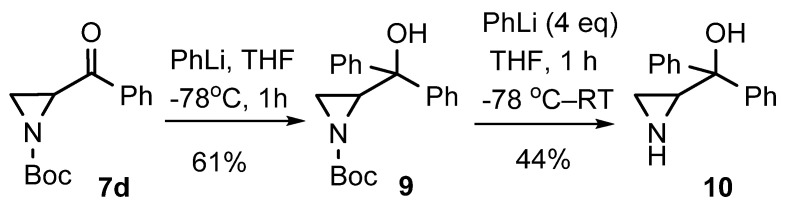
Synthesis of carbinols **9** and **10** from ketone **7d**.

To test the scope and limitations of the current reaction of N-Boc 2-carbonylaziridines with PhLi, we have tested two additional substrates: ketone **7d** and benzyl ester **1c**. The interaction of ketone **7d** with 1 eq. of PhLi formed Boc-protected carbinol **9**, but an excess of PhLi led to N-Boc deprotection and gave carbinol **10** (Scheme 5) in a moderate yield.

**Scheme 6:**
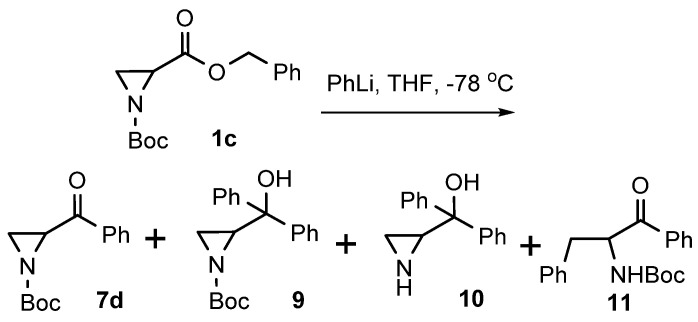
Synthesis of ketones **7d**, **11** and carbinols **9**, **10** from benzyl ester **1c**.

The reaction of benzyl ester **1c** with PhLi was more complex, and a mixture of ketone **7d** and carbinol **9** was obtained (Scheme 6, Table 5, entries 1 and 2). The yield of the carbinol **9** increased by using an excess of PhLi (entry 2). A longer reaction time decreased the total yield and led to the formation of deprotected carbinol **10** (entry 4).

**Scheme 7:**
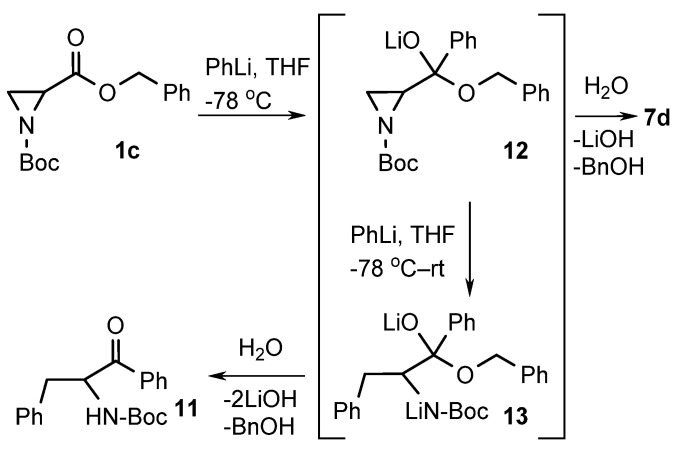
Formation of ketones **7d** and **11** from benzyl ester **1c** through intermediates **12**, **13**.

During the warming of the reaction mixture to RT, the aziridine ring cleavage by-product **11** [21] was isolated in a 9% yield (entry 5). The proposed tetrahedral intermediate 12 (Scheme 7) was formed from ester **1c** in a nucleophilic attack with PhLi and, after hydrolysis, yielded the target ketone **7d**. At the same time, the intermediate **12** reacted with an excess of PhLi and gave ring-opening intermediate **13**, which, under hydrolysis conditions, turned to ketone **11**.

## 3. Materials and Methods

The ^1^H NMR spectra were recorded on Varian Mercury 200 (200 MHz) and Varian Mercury plus 400 (400 MHz) spectrometers. TMS or CHCl_3_ (δ 7.26 ppm, solvent CDCl_3_) were used as an internal standard. The ^13^C NMR spectra were recorded on a Varian Mercury plus 400 (101 MHz) spectrometer. CHCl_3_ (δ 77.16 ppm) was used as an internal standard. HRMS were measured on a Waters Synapt G2–Si mass spectrometer. IR spectra were recorded on a Shimadzu FTIR IR Prestige-21 spectrometer. Melting points were determined on a Gallenkamp heating stage; uncorrected values were shown. TLC was carried out on DC Alufolien plates of Kieselgel 60. Column chromatography was carried out on Kieselgel (Acros), 0.023–0.070 mm, pore diameter ca 6 nm. Tetrahydrofuran was distilled from Na/benzophenone. Esters **1a** and **1b** were prepared according to the literature from aziridine-2-carboxylate in reactions with Boc-anhydride [22] and trityl-chloride [23], respectively. Ester **1c** was obtained in the reaction of benzyl aziridine-2-carboxylate with Boc-anhydride [23]. Activated bis-Boc amides **2a,b** were prepared by the 4-dimethylaminopyridine (DMAP)-catalyzed reaction of the corresponding amide with Boc_2_O [24]. Hydroxylamines **3b–f** were synthesized from corresponding esters **1** as described in [12] or from activated bis-Boc amides **2** according to the published procedure [24]. Aziridine-2-carboxylic acid amides **4a** and **4b** were obtained from the aziridine-2-carboxylic acid dimethylamide in accordance with the previous reports [8]. All reactions involving organolithium compounds were carried out under an argon atmosphere. Diffraction data for structures **6d** and **6d1** were collected at −90 °C on a Bruker–Nonius Kappa CCD diffractometer using graphite monochromated Mo-Kα radiation (λ = 0.71073 Å). The crystal structure was solved by direct methods [25] and refined by full-matrix least-squares [26]. All non-hydrogen atoms were refined in an anisotropic approximation. H-atoms were refined by the riding model.

The general procedure for the reaction of ester **1c**, amides **4a**,**b**, ketones **5a**,**d**,**e** and **7d** with organolithium reagents was the following. A round-bottomed flask was heated for 5 h at 150 °C and cooled in a stream of argon to room temperature. Then, 20 mL abs. THF was placed into the flask, and 0.5–6.5 mmol of aziridine substrate **1c**, **4a**,**b**, **5a**,**d**,**e** or **7d** was added. The resulting solution was cooled with dry ice and acetone in a stream of argon to −78 °C. To the cooled and stirred substrate solution, an organolithium reagent (solution in hexanes or toluene) was added dropwise over 10 min. The resulting reaction mixture was stirred at −78 °C under argon for 1, 2, 12 or 24 h, then 20 mL of a 1:1 water-THF mixture was added, and the reaction mixture was warmed to RT. The mixture was partitioned between water (50 mL) and diethyl ether (50 mL), the layers were separated and the organic layer was extracted with diethyl ether (3 × 20 mL). The combined ether extracts were washed with water (2 × 20 mL) and brine (20 mL), dried over Na_2_SO_4_, filtered off and evaporated under reduced pressure. The obtained crude product was purified with column chromatography on silica (200 mL silica for 1 g of material) with PE-EtOAc eluent.

Experimental details for compounds **2**,**3**, **5**–**11** and characterization data for com-pounds **1**–**11** including 1H and 13C NMR, IR spectra, HRMS or elemental analysis and crystal data for compounds 6c and 6d1 are presented in supplementary materials.

## 4. Conclusions

In summary, we have demonstrated that both N-trityl and the more electron-deficient N-Boc *N,N*-dimethyl aziridine-2-carboxamides react regioselectively with organolithium reagents. No significant side reactions were observed if reaction mixtures were quenched before warming up. The reaction with one equivalent of organolithium reagent is selective to amide carbonyl at a low (−78 °C) temperature.

The best observed reaction conditions for-2-aziridinylketones are −78 °C temperature and 1–2 h reaction time for aliphatic N-trityl-2-aziridinylketones **5a–d** and N-Boc ketones **7a**–**d**. In the case of aromatic ketone **5e**, a prolonged reaction time (12 h) is necessary. For both symmetrical and unsymmetrical carbinols **6a**–**f**, a longer (12 h) reaction time is preferred.

Therefore, it has been demonstrated that this is a simple and practical route to selectively obtaining 2-aziridinylketones and both symmetrical and unsymmetrical 2-aziridinylcarbinols. *N,N*-Dimethyl aziridine-2-carboxamides are better substrates than the corresponding esters or Weinreb amides. The N-Boc-protecting group nucleophilic cleavage with an excess of phenyllithium was demonstrated in the case of 2-aziridinylketone and the corresponding carbinol. This method may serve as a special Boc-protecting group cleavage for acid-sensitive substrates.

## Figures and Tables

**Table 1:** Reaction conditions and yields of hydroxylamines **3b–f**.

#	SM	RC	Eq. 3a	SM (%)	3b,c (%)	3d (%)	3e (%)	3f (%)
1	**1a**	A	3	-	-	55	36	-
2	**1a**	A	1	35	-	25	20	-
3	**1a**	B	3	52	Traces	-	-	-
4	**1b**	A	3	-	-	-	-	62
5	**1b**	A	1	39	-	-	-	42
6	**2a**	C	2	56	31	-	-	-
7	**2a**	C	2	80	Traces	-	-	-

Reaction conditions (RC): A: MeNHOMe × HCl, AlMe_3_, DCM, −10 °C–RT, 2 h; B: MeNHOMe, AlMe_3_, DCM, −10 °C–RT, 2 h; C: MeNHOMe, DCM, reflux, 120 h.

**Table 2:** Reaction conditions and yields of ketones **5a**–**e**.

#	Eq. RLi	Time (h)	R	Product	Yield (%)
1	1	2	Me	**5a**	77
2	2	2	Me	**5a**	93
3	2	24	Me	**5a**	70
4	1	2	*n*-Bu	**5b**	39
5	2	2	*n*-Bu	**5b**	44
6	2	24	*n*-Bu	**5b**	38
7	1	2	*i*-Bu	**5c**	41
8	1	24	*i*-Bu	**5c**	36
9	2	2	*i*-Bu	**5c**	43
10	2	24	*i*-Bu	**5c**	47
11	1	2	*t*-Bu	**5d**	30
12	2	2	*t*-Bu	**5d**	32
13	2	24	*t*-Bu	**5d**	29
14	1	2	Ph	**5e**	51
15	1	12	Ph	**5e**	79
16	2	2	Ph	**5e**	50
17	2	12	Ph	**5e**	81
18	4	24	Ph	**5e**	78

**Table 3:** Reaction conditions and yields of carbinols **6a–f** and phenol **6d1**.

#	Eq. R^1^Li	Time (h)	R	R^1^	Product	Yield (%)
1	1	1	Me	Me	**6a**	33
2	1	12	Me	Me	**6a**	35
3	4	1	Me	Me	**6a**	33
4	4	12	Me	Me	**6a**	32
5	1	1	Ph	Ph	**6b**	82
6	1	12	Ph	Ph	**6b**	85
7	4	1	Ph	Ph	**6b**	79
8	4	12	Ph	Ph	**6b**	99
9	4	24	Ph	Ph	**6b**	98
10	1	1	*t*-Bu	*t*-Bu	**6c**	30
11	1	12	*t*-Bu	*t*-Bu	**6c**	68
12	4	12	*t*-Bu	*t*-Bu	**6c**	70
13	4	12	Me	*t*-Bu	**6d** *	25
14	4	12	*t*-Bu	Me	**6d1**	52
15	4	12	*t*-Bu	Ph	**6e**	100
16	1	12	Ph	Me	**6f**	88
17	4	12	Ph	Me	**6f**	61

* in ~1:1 mixture with **6d1.**

**Table 4:** Reaction conditions and yields of compounds **7a–d** and **8**.

#	Eq. RLi	R	Product 7 (%)	Product 8 (%)
1	1	Me	**7a** (62)	-
2	2	Me	**7a** (8)	-
3	1	*n*-Bu	**7b** (75)	-
4	1	*t*-Bu	**7c** (82)	-
5	1	Ph	**7d** (60)	-
6	4	Ph	**7d** (51)	35
7	4 *	Ph	-	66

* Warmed to room temperature before water addition.

**Table 5:** Reaction conditions and yields of compounds **7d**, **9–11**.

#	Eq. PhLi	Time (h)	Product 7d (%)	Product 9 (%)	Product 10 (%)	Product 11 (%)
1	1	1	26	5	-	-
2	2	1	10	40	-	-
3	4	1	8	52	-	-
4	4	12	-	5	22	-
5	4	12 *	-	2	15	9

* Warmed to room temperature before water addition.

## Data Availability

Supplementary materials are included and represent original data obtained by the authors.

## Supplementary Materials

The following are available online at https://www.mdpi.com/article/10.3390/ijms222313145/s1.
