# Supplementary material for: An Easy Route to Aziridine Ketones and Carbinols
Source: Int J Mol Sci. 2021 Dec 5;22(23):13145. doi: 10.3390/ijms222313145 (PMC8658269; doi:10.3390/ijms222313145)
Supplement: Supplementary file 1 [file ijms-22-13145-s001.zip › ijms-1471021-Supplementary-IMG_FIXED2.pdf]

An easy route to aziridine ketones and carbinols

Experimental details for compounds 2,3, 5-11. Characterization data for compounds 1-11

***tert*-Butyl 2-(bis(*tert*-butoxycarbonyl)carbamoyl)aziridine-1-carboxylate (2a):**

Prepared after literature procedure [21, 26] from aziridine-2-carboxamide (0.50 g; 5.81 mmol), Boc_2O (3.80 g; 17.42 mmol) and DMAP (0.07 g; 0.58 mmol). Yield 1.89 g (84%). Title compound **2a** was isolated by column chromatography on 450 mL silica with PE-EtOAc (3:1) eluent as a white crystalline solid, mp 67°C; R_f = 0.6 (PE-EtOAc; 3:1). ^{1}H NMR (400 MHz, $CDCl_3$) δ 3.99 (1H, dd, *J* = 5.5, 3.2 Hz, C_2H), 2.57 (1H, dd, *J* = 3.2, 1.6 Hz, C_3H), 2.46 (1H, dd, *J* = 5.5, 1.6 Hz, C_3H), 1.52 (18H, s, *t*-butyl), 1.44 (9H, s, *t*-butyl). ^{13}C NMR (101 MHz, $CDCl_3$) δ 159.9, 149.9, 149.2, 85.6, 82.2, 36.1, 32.6, 27.9, 27.7. HRMS (EI) $[M+Na]^+$, found 409.1953. $C_{18}H_{30}N_2O_7Na$ requires 409.1951.

***tert*-Butyl (*tert*-butoxycarbonyl)(1-tritylaziridine-2-carbonyl)carbamate (2b):**

Prepared after literature procedure [8] from N-tritylaziridine-2-carboxamide (7.08 g, 21.55 mmol), Boc_2O (9.41 g, 43.11 mmol) and DMAP (0.26 g, 2.16 mmol). Yield 8.72 g (77%). Title compound **2b** was isolated by column chromatography on 500 mL silica with PE-EtOAc (10:1) eluent as a white crystalline solid, mp 113°C, R_f = 0.4 (PE-EtOAc, 10:1). ^{1}H NMR (400 MHz, $CDCl_3$) δ 7.50-7.40 (6H, m, Ar), 7.25-7.10 (9H, m, Ar), 2.80 (1H, dd, *J* = 6.1, 2.7 Hz, C_2H), 2.32 (1H, dd, *J* = 2.7, 1.8 Hz, C_3H), 1.40-1.36 (19H, m, C_3H and *t*-butyl). ^{13}C NMR (101 MHz, $CDCl_3$) δ 171.8, 149.5, 143.9, 129.6, 127.7, 127.0, 84.2, 74.7, 33.0, 29.9, 27.7. HRMS (EI) $[M+Na]^+$, found 551.2515. $C_{32}H_{36}N_2O_5Na$ requires 551.2522.

***tert*-Butyl 2-(methoxy(methyl)carbamoyl)aziridine-1-carboxylate (3b):**

Prepared after literature procedure [8] from *bis*-Boc amide **2a** (0.08 g, 0.21 mmol), hydroxylamine **3a** (prepared from MeNHOMe*HCl (0.04 g, 0.42 mmol) and NaOH (0.017 g, 0.43 mmol)). Yield 0.015 g (31%) after procedure [8] (Table1, entry 6) and <1% (traces amount) after procedure [4] (Table 1, entry 3). Title compound **3b** was isolated by column chromatography on 200 mL silica with PE-EtOAc (3:1) eluent as a white foam R_f = 0.2 (PE-EtOAc, 3:1). ^{1}H NMR (400 MHz, $CDCl_3$) δ 3.83 (3H, s, NMe), 3.59-3.53 (1H, m, C_2H), 3.26 (3H, s, OMe), 2.61 (1H, dd, *J* = 3.1, 1.5 Hz, C_3H), 2.37 (1H, dd, *J* = 5.2, 1.5 Hz, C_3H), 1.45 (9H, s, *t*-butyl). ^{13}C NMR (101 MHz, $CDCl_3$) δ 168.9, 159.6, 86.1, 52.5, 34.8, 31.3, 27.8. HRMS (EI) $[M+Na]^+$, found 253.1164. $C_{10}H_{18}N_2O_4Na$ requires 253.1164.

N-Methoxy-N-methyl-1-tritylaziridine-2-carboxamide (3c):

Prepared after literature procedure [8] from *bis*-Boc amide **2b** (0.25 g, 0.47 mmol), hydroxylamine **3a** (prepared from MeNHOMe*HCl (0.09 g, 0.94 mmol) and NaOH (0.04 g, 0.95 mmol)). Yield 0.005g of impure material (Table1, entry 7). Title compound **3c** was isolated by column chromatography on 200 mL silica with PE-EtOAc (3:1) eluent as a white foam containing impurities R_f = 0.2 (PE-EtOAc, 3:1). ^{1}H NMR (400 MHz, $CDCl_3$) δ 7.51-7.42 (6H, m, Ar), 7.25-7.10 (9H, m, Ar), 3.30 (3H, s, NMe), 3.13 (3H, s, OMe), 2.35-2.24 (2H, m, C_2H and C_3H), 1.33 (1H, dd, *J* = 6.1, 1.8 Hz, C_3H). ^{13}C NMR (101 MHz, $CDCl_3$) δ 144.0, 129.6, 127.7, 122.0, 84.3, 74.6, 61.5, 28.1, 27.7. HRMS (EI) $[M+Na]^+$, found 395.1734 $C_{24}H_{24}N_2O_2Na$ requires 395.1735.

***tert*-Butyl (3-chloro-1-(methoxy(methyl)amino)-1-oxopropan-2-yl)carbamate (3d):**

Prepared after literature procedure [4] from ester **1a** (0.60 g, 2.98 mmol) MeNHOMe*HCl and $AlMe_3$ (1 M in heptane). Yield 0.44 g, 55% (0.87 g, 8.91 mmol, 3 eq MeNHOMe*HCl, 8.90 mL $AlMe_3$, Table 1, entry 1); 0.15 g, 25% (0.29 g, 2.98 mmol, 1 eq MeNHOMe*HCl, 2.97 mL $AlMe_3$, Table 1, entry 2). Title compound **3d** was isolated by column chromatography on 200 mL silica with PE-EtOAc (3:1 then 1:1) eluent as a white crystalline solid, mp 134°C R_f = 0.3 (PE-EtOAc, 1:1). ^{1}H NMR (400 MHz, $CDCl_3$) δ 5.56-5.46 (1H, m, NH), 5.03-4.93 (1H, m, CH), 3.82-3.78 (2H, m, CH_2), 3.77 (3H, s, NMe), 3.25 (3H, s, OMe), 1.45 (9H, s, *t*-Bu). ^{13}C NMR (101 MHz, $CDCl_3$) δ 169.2, 155.2, 80.4, 61.8, 51.7, 44.7, 32.4, 28.5. Anal. Calcd for $C_{10}H_{19}ClN_2O_4$: C 45.03, H 7.18, N 10.50%. Found: C 45.62, H 7.19, N 10.17%.

***tert*-Butyl (3,7-dimethyl-4-oxo-2,8-dioxa-3,7-diazanonan-5-yl)carbamate (3e):**

Prepared after literature procedure [4] from ester **1a** (0.60 g, 2.98 mmol), MeNHOMe*HCl and $AlMe_3$ (1 M in heptane). Yield 0.31 g, 36% (0.87 g, 8.91 mmol, 3 eq MeNHOMe*HCl, 8.90 mL $AlMe_3$, Table 1, entry 1); 0.17 g, 20% (0.29 g, 2.98 mmol, 1 eq MeNHOMe*HCl, 2.97 mL $AlMe_3$, Table 1, entry 2). Title compound **3d** was isolated by column chromatography on 200 mL silica with PE-EtOAc (3:1 then 1:1) eluent as a white crystalline solid, mp 116°C R_f = 0.2 (PE-EtOAc, 1:1). ^{1}H NMR (400 MHz, $CDCl_3$) δ 5.51-5.41 (1H, m, NH), 4.84-4.73 (1H, m, CH), 3.76 (3H, s, NMe), 3.43 (3H, s, OMe), 3.19 (3H, s, OMe), 3.01-2.91 (1H, m, CH_2), 2.88-2.82 (1H, m, CH_2), 2.56 (3H, s, NMe), 1.43 (9H, s, *t*-Bu). ^{13}C NMR (101 MHz, $CDCl_3$) δ 171.9, 155.7, 79.7, 61.7, 61.6, 59.5, 50.0, 45.3, 32.6, 28.5. HRMS (EI) $[M+Na]^+$, found 314.1707. $C_{12}H_{25}N_3O_5Na$ requires 314.1692.

N,O-dimethyl-N-tritylhydroxylamine (3f):

Prepared after literature procedure [4] from ester **1b** (0.14 g, 0.41 mmol), MeNHOMe*HCl and $AlMe_3$ (1 M in heptane). Yield 0.08 g, 62% (0.12 g, 1.23 mmol, 3 eq MeNHOMe*HCl, 1.23 mL $AlMe_3$, Table 1, entry 4); 0.05 g, 42% (0.04 g, 0.41 mmol, 1 eq MeNHOMe*HCl, 0.41 mL $AlMe_3$, Table 1, entry 5). Title compound **3f** was isolated by column chromatography on 50 mL silica with PE-EtOAc (3:1) eluent as a white amorphous solid R_f = 0.8 (PE-EtOAc, 3:1). ^1H NMR (400 MHz, $CDCl_3$) δ 7.52-7.46 (6H, m, Ar), 7.26-7.11 (9H, m, Ar), 3.52 (3H, s, OMe), 2.33 (3H, s, NMe). ^{13}C NMR (101 MHz, $CDCl_3$) δ 143.1, 129.9, 127.4, 126.4, 80.0, 58.8, 38.2. HRMS (EI) $[M+H]^+$, found 304.1704. $C_{21}H_{22}NO$ requires 304.1701.

1-(1-Triphenylmethylaziridine-2-yl) ethanone (5a):

Prepared after general procedure from amide **4a** (2.04 g, 5.72 mmol), MeLi (1.5 M solution in hexanes), 10 mL THF. Yield 1.44 g, 77% (2 h, 1 eq, 5.72 mmol, 3.86 mL MeLi; Table 2, entry 1); 1.75 g, 93% (2 h, 2 eq, 11.44 mmol, 7.63 mL MeLi; Table 2, entry 2); 1.32 g, 70% (24 h, 2 eq, 11.44 mmol, 7.63 mL MeLi, Table 2, entry 3). Title compound **5a** was isolated by column chromatography on 400 mL silica with PE-EtOAc (4:1) eluent as a white crystalline solid, mp 105°C, R_f = 0.5 (PE-EtOAc, 4:1). IR ($CHCl_3$) 3057, 3021, 1699, 1489, 1448, 1354, 1218, 1003, 754, 708 cm^{-1}. ^1H NMR (400 MHz, $CDCl_3$) δ 7.52-7.39 (6H, m, Ar), 7.36-7.17 (9H, m, Ar), 2.86 (3H, s, methyl), 2.21 (1H, dd, *J* = 2.6, 0.9 Hz, C_3H), 1.98 (1H, dd, *J* = 6.3, 2.6 Hz, C_3H), 1.44 (1H, dd, *J* = 6.3, 0.9 Hz, C_2H). ^{13}C NMR (101 MHz, $CDCl_3$) δ 207.9, 143.7, 129.4, 128.1, 127.9, 127.2, 74.7, 39.6, 29.2, 25.2. HRMS (EI) $[M+H]^+$, found 328.1694. $C_{23}H_{22}NO$ requires 328.1701.

1-(1-Triphenylmethylaziridine-2-yl) pentane-1-one (5b):

Prepared after general procedure from amide **4a** (2.26 g, 6.57 mmol), *n*-BuLi (2.5 M solution in hexanes), 10 mL THF. Yield 0.95 g, 39% (2 h, 1 eq, 6.57 mmol, 2.54 mL *n*-BuLi; Table 2, entry 4); 1.07 g, 44% (2 h, 2 eq, 13.14 mmol, 5.07 mL *n*-BuLi; Table 2, entry 5); 0.92 g, 38% (24 h, 2 eq, 13.14 mmol, 5.07 mL *n*-BuLi; Table 2, entry 6). Title compound **5b** was isolated by column chromatography on 400 mL silica with PE-EtOAc (4:1) eluent as a white crystalline solid, mp 95°C, R_f = 0.6 (PE-EtOAc, 4:1). IR ($CHCl_3$) 3057, 2958, 2931, 2872, 1706, 1489, 1448, 1218, 1004, 751, 710 cm^{-1}. ^1H NMR (400 MHz, $CDCl_3$) δ 7.34-7.16 (15H, m, Ar), 2.73-2.64 (1H, m, *n*-butyl), 2.54-2.44 (1H, m, *n*-butyl); 2.19 (1H, dd, *J* = 2.9, 1.4 Hz, C_3H), 2.00 (1H, dd, *J* = 6.3, 2.9 Hz, C_3H), 1.67-1.51 (2H, m, *n*-butyl), 1.41 (1H, dd, *J* = 6.3, 1.4 Hz, C_2H), 1.40-1.30 (2H, m, *n*-butyl); 0.93 (3H, t, *J* = 7.1Hz, *n*-butyl). ^{13}C NMR (101 MHz, $CDCl_3$) δ 209.5, 143.7, 129.5, 127.8, 127.1, 74.6, 38.6, 38.0, 29.3, 25.8, 22.6, 14.1. HRMS (EI) $[M+H]^+$, found 370.2169. $C_{26}H_{28}NO$ requires 370.2171.

2-Methyl-1-(1-triphenylmethylaziridine-2-yl)-butane-1-one (mixture of diastereomers) **(5c):**

Prepared after general procedure from amide **4a** (0.54 g, 1.50 mmol), *s*-BuLi (1.25 M solution in hexanes), 15 mL THF. Yield 0.23g, 41% (2h, 1eq, 1.50 mmol, 1.20mL *s*-BuLi; Table 2, entry 7); 0.20 g, 36% (24 h, 1 eq, 1.50 mmol, 1.20 mL *s*-BuLi; Table 2, entry 8); 0.24g, 43% (2h, 2eq, 3.00 mmol, 2.40mL *s*-BuLi; Table 2, entry 9) 0.26 g, 47% (24 h, 2 eq, 3.00 mmol, 2.40 mL *s*-BuLi; Table 2, entry 10). Title compound **5c** was isolated by column chromatography on 150 mL silica with PE-EtOAc (9:1) eluent as a white amorphous solid, R_f = 0.5 (PE-EtOAc, 4:1). IR ($CHCl_3$) 3350, 3056, 3022, 2968, 2933, 2876, 1713, 1598, 1489, 1466, 1381, 1218, 757, 704 cm^{-1}. ^1H NMR (400 MHz, $CDCl_3$) δ (mixture of isomers) 7.56-7.40 (6H, m, Ar), 7.35-7.15 (9H, m, Ar), 2.68 (1H, heptet, *J* = 7.0 Hz, *s*-Bu), 2.23-2.20 (1H, m, C_2H), 2.03 (1H, dd, *J* = 6.3, 2.8 Hz, C_3H), 1.74-1.54 (1H, m, *s*-Bu), 1.43 (1H, dd, *J* = 6.2, 1.7 Hz, C_3H), 1.36 (1H, duplet of heptets, *J* = 7.4, 2.4 Hz, *s*-Bu), 1.06 and 1.02 (1.5H and 1.5H, d and d, *J* = 7.0 and 7.0 Hz, *s*-Bu), 0.86 (3H, dt, *J* = 7.4, 2.4 Hz, *s*-Bu). ^{13}C NMR (101 MHz, $CDCl_3$) δ 212.2, 212.0, 148.5, 147.0, 144.4, 143.8, 129.5, 128.2, 128.1, 127.7, 127.3, 127.1, 126.8, 44.6, 45.8, 45.3, 37.0, 36.9, 30.2, 30.0, 26.3, 25.7, 16.5, 16.0, 11.9. HRMS (EI) $[M+Na]^+$, found 392.1985. $C_{26}H_{27}NONa$ requires 392.1990.

2.2-Dimethyl-1-(1-triphenylmethylaziridine-2-yl)-propane-1-one (5d):

Prepared after general procedure from amide **4a** (2.14 g, 6.00 mmol), *t*-BuLi (1.5 M solution in hexanes), 15 mL THF. Yield 0.66 g, 30% (2 h, 1 eq, 6.00 mmol, 4.00 mL *t*-BuLi; Table 2, entry 11); 0.71 g, 32% (2 h, 2 eq, 12.00 mmol, 8.00 mL *t*-BuLi; Table 2, entry 12); 0.64 g, 29% (24 h, 2 eq, 12.00 mmol, 8.00 mL *t*-BuLi; Table 2, entry 13). Title compound **5d** was isolated by column chromatography on 300 mL silica with PE-EtOAc (10:1) eluent as a white crystalline solid, mp 100°C, R_f = 0.4 (PE-EtOAc, 5:1). IR ($CHCl_3$) 3057, 3021, 2969, 2932, 1713, 1487, 1476, 1395, 1218, 1070, 1014, 756, 707 cm^{-1}. ^1H NMR (400 MHz, $CDCl_3$) δ 7.57-7.42 (6H, m, Ar), 7.37-7.15 (9H, m, Ar), 2.29-2.16 (2H, m, C_3H), 1.44 (1H, dd, *J* = 6.1, 2.4 Hz, C_2H), 1.04 (9H, s, *t*Bu). ^{13}C NMR (101 MHz, $CDCl_3$) δ 212.0, 144.0, 129.6, 127.7, 127.1, 74.7, 44.4, 32.7, 31.1, 26.2. Anal. Calcd for $C_{26}H_{27}NO$: C 84.51, H 7.37, N 3.79%. Found: C 84.43, H 7.51, N, 3.73%.

(1-Triphenylmethylaziridine-2-yl)-phenyl-methanone (5e):

Prepared after general procedure from amide **4a** (3.00 g, 8.42 mmol), PhLi (2.00 M solution in toluene), 21 mL THF. Yield 1.67 g, 51% (2 h, 1 eq, 8.42 mmol, 4.20 mL PhLi; Table 2, entry 14); 2.59 g, 79% (12 h, 1 eq, 8.42 mmol, 4.20 mL PhLi; Table 2, entry 15); 1.64 g, 50% (2 h, 2 eq, 16.84 mmol, 8.40 mL PhLi; Table 2, entry 16); 2.66 g, 81% (12 h, 2 eq, 16.84 mmol, 8.40 mL PhLi; Table 2, entry 17); 2.56 g, 78% (24 h, 4 eq, 33.66 mmol, 16.80

mL PhLi; Table 2, entry 18). Title compound **5e** was isolated by column chromatography on 400 mL silica with PE-EtOAc (10:1) eluent as a white crystalline solid, mp 129°C, R_f = 0.3 (PE-EtOAc, 7:1). IR ($CHCl_3$) 3084, 3063, 3019, 1965, 1683, 1598, 1489, 1447, 1397, 1231, 1033, 754, 708 cm^{-1}. ^1H NMR (400 MHz, $CDCl_3$) δ 7.91-7.11 (20H, m, Ar), 2.74 (1H, dd, *J* = 6.3, 2.8 Hz, C_2H), 2.43 (1H, unresolved dd, *J* = 2.0 Hz, C_3H), 1.60 (1H, dd, *J* = 6.3, 2.0 Hz, C_3H). ^{13}C NMR (101 MHz, $CDCl_3$) δ 197.1, 143.9, 137.4, 133.3, 129.6, 128.7, 128.4, 127.8, 127.1, 74.9, 35.0, 30.9. Anal. Calcd for $C_{28}H_{23}NO$: C 86.34, H 5.96, N 3.60%. Found: C 86.08, H 5.92, N 3.49%.

2-(1-Triphenylmethylaziridine-2-yl)-propan-2-ol (6a):

Prepared after general procedure from ketone **5a** (0.44 g, 1.34 mmol), MeLi (1.60 M solution in hexanes), 20 mL THF. Yield 0.15 g, 33% (1 h, 1 eq, 1.34 mmol, 0.84 mL MeLi; Table 3, entry 1); 0.16 g, 35% (12 h, 1 eq, 1.34 mmol, 0.84 mL MeLi; Table 3, entry 2); 0.15 g, 33% (1 h, 4 eq, 5.36 mmol, 3.36 mL MeLi; Table 3, entry 3); 0.15 g, 32% (12 h, 4 eq, 5.36 mmol, 3.36 mL MeLi; Table 3, entry 4). Title compound **6a** was isolated by column chromatography on 140 mL silica with PE-EtOAc (10:1) eluent as a white crystalline solid, mp 120°C, R_f = 0.4 (PE-EtOAc, 9:1). IR ($CHCl_3$) 3463, 3056, 2974, 2929, 1596, 1489, 1448, 1332, 1220, 1152, 1025, 954, 756, 707 cm^{-1}. ^1H NMR (400 MHz, $CDCl_3$) δ 7.58-7.43 (6H, m, Ar), 7.32-7.16 (9H, m, Ar), 3.04 (1H, s, OH), 1.89 (1H, unresolved dd, *J* = 3.4 Hz, C_3H), 1.36 (1H, dd, *J* = 6.2, 3.4 Hz, C_2H), 1.15 (3H, s, Me), 1.09 (3H, s, Me), 1.11-1.08 (1H, m, C_3H). ^{13}C NMR (101 MHz, $CDCl_3$) δ 144.4, 129.6, 127.7, 127.0, 74.1, 67.6, 42.5, 29.6, 26.6, 23.6. HRMS (EI) $[M+H]^+$, found 344.2011. $C_{24}H_{26}NO$ requires 344.2014.

Diphenyl-(1-triphenylmethylaziridine-2-yl)-methanol (6b):

Prepared after general procedure from ketone **5e** (0.50 g, 1.28 mmol), PhLi (2.00 M solution in toluene), 15 mL THF. Yield 0.49 g, 82% (1 h, 1 eq, 1.28 mmol, 0.64 mL PhLi; Table 3, entry 5); 0.51 g, 85% (12 h, 1 eq, 1.28 mmol, 0.64 mL PhLi; Table 3, entry 6); 0.47 g, 79% (1 h, 4 eq, 5.12 mmol, 2.56 mL PhLi; Table 3, entry 7); 0.459 g, 99% (12 h, 4 eq, 5.12 mmol, 2.56 mL PhLi; Table 3, entry 8) 0.59 g, 98% (24 h, 4 eq, 5.12 mmol, 2.56 mL PhLi; Table 3, entry 9). Title compound **6b** was isolated by column chromatography on 170 mL silica with PE-EtOAc (10:1) eluent as a white crystalline solid, mp 154°C, R_f = 0.5 (PE-EtOAc, 9:1). IR ($CHCl_3$) 3401, 3087, 3062, 3024, 1953, 1598, 1495, 1447, 1338, 1217, 1184, 1024, 991, 764, 707 cm^{-1}. ^1H NMR (400 MHz, $CDCl_3$) δ 7.48-7.10 (25H, m, Ar), 4.45 (1H, s, OH), 2.38 (1H, dd, *J* = 6.3, 3.3 Hz, C_2H), 2.11 (1H, unresolved dd, *J* = 3.3 Hz, C_3H), 1.36 (1H, unresolved dd, *J* = 6.3 Hz, C_3H). ^{13}C NMR (101 MHz, $CDCl_3$) δ 147.1, 145.6, 143.8, 129.4, 128.1, 127.9, 127.6, 127.0, 126.9, 126.4, 126.1, 74.2, 41.7, 24.0. Anal. Calcd for $C_{34}H_{29}NO$: C 87.33, H 6.25, N 3.00%. Found: C 87.03, H 6.12, N 2.85%.

2,2,4,4-Tetramethyl-(1-triphenylmethylaziridine-2-yl)-pentane-3-ol (6c):

Prepared after general procedure from ketone **5d** (0.50 g, 1.35 mmol), *t*-BuLi (1.50 M solution in hexanes), 15 mL THF. Yield 0.17 g, 30% (1 h, 1 eq, 1.35 mmol, 0.90 mL *t*-BuLi; Table 3, entry 10); 0.39 g, 68% (12 h, 1 eq, 1.35 mmol, 0.90 mL *t*-BuLi; Table 3, entry 11); 0.41 g, 70% (12 h, 4 eq, 5.40 mmol, 3.60 mL *t*-BuLi; Table 3, entry 12). Title compound **6c** was isolated by column chromatography on 200 mL silica with PE-EtOAc (10:1) eluent as a white crystalline solid, mp 167°C, R_f = 0.5 (PE-EtOAc, 10:1). IR ($CHCl_3$) 3467, 3061, 3018, 2965, 2913, 2875, 1594, 1489, 1449, 1391, 1216, 1004, 974, 742, 709 cm^{-1}. ^1H NMR (400 MHz, $CDCl_3$) δ 7.56-7.48 (6H, m, Ar), 7.29-7.21 (6H, m, Ar), 7.20-7.14 (3H, m, Ar), 2.87 (1H, s, OH), 2.31 (1H, unresolved dd, *J* = 3.3 Hz, C_3H), 1.42-1.31 (2H, m, C_3H and C_2H), 0.92 (9H, s, *t*-Bu), 0.86 (9H, s, *t*-Bu). ^{13}C NMR (101 MHz, $CDCl_3$) δ 144.3, 129.8, 127.6, 126.7, 77.0, 75.6, 42.2, 41.4, 40.9, 30.2, 30.0, 26.5. Anal. Calcd for $C_{30}H_{37}NO$: C 84.25, H 8.72, N 3.27%. Found: C 83.93, H 8.98, N 3.27%. Crystal data: monoclinic, a = 25.1187(8), b = 8.7502(3), c = 23.5529(8) Å, β = 105.528(2)°; V = 4987.8(3) Å^3, Z = 8, μ = 0.067 mm^{-1}, Dcalc = 1.139g·cm^{-3}; space group is *C*2/*c*. For further details, see crystallographic data deposited at the Cambridge Crystallographic Data Centre as Supplementary Publications Numbers CCDC 1866495. Copies of the data can be obtained, free of charge, on application to CCDC, 12 Union Road, Cambridge CB2 1EZ, UK.

Mixture of 3,3-Dimethyl-2-(1-trityl-aziridin-2-yl)-butan-2-ol (6d) (mixture of diastereomers) and phenol (6d1):

Prepared after general procedure from ketone **5a** (0.15 g, 0.46 mmol), *t*-BuLi (1.50 M solution in hexanes), 15 mL THF. Yield 0.09 g of mixture, ~25% (12 h, 4 eq, 1.84 mmol, 1.24 mL *t*-BuLi; Table 3, entry 13). Title compound **6d** in 1:1 mixture with compound **6d1** was isolated by column chromatography on 50 mL silica with PE-EtOAc (10:1) eluent as a colourless oil, R_f = 0.4 (PE-EtOAc 10:1). IR ($CHCl_3$) 2967, 1595, 1491, 1448, 1394, 1367, 1330, 1235, 1218, 1121, 1095, 1021, 912, 758, 708 cm^{-1}. ^1H NMR (400 MHz, $CDCl_3$) δ 7.58-7.46 (5H, m, Ar), 7.32-7.11(10H, m, Ar), 3.02 (0.5H, brs, OH, **6d**), 2.38 (0.5H, brs, OH, **6d1**), 2.13 (0.5H, unresolved dd, *J* = 3.4 Hz, C_3H, **6d**), 1.80 (0.5H, unresolved dd, *J* = 3.3 Hz, C_3H, **6d1**), 1.57 (0.5H, dd, *J* = 6.6, 3.3 Hz, C_2H, **6d1**), 1.37 (0.5H, dd, *J* = 6.1, 3.6 Hz, C_2H, **6d**), 1.20 (0.5H, unresolved dd, *J* = 6.6 Hz, C_3H, **6d**), 1.15 (0.5H, unresolved dd, *J* = 6.6 Hz, C_3H, **6d1**), 1.14 (1.5H, s, Me, **6d**), 1.10 ((1.5H, s, Me, **6d1**), 0.83 (4.5H, s, *t*-Bu, **6d**), 0.78 (4.5H, s, *t*-Bu, **6d1**). ^{13}C NMR (101 MHz, $CDCl_3$) δ 144.4, 144.3, 129.7, 129.6, 127.7, 127.6, 126.9, 74.7, 74.5, 74.3, 72.6, 40.6, 39.3, 37.6, 37.5, 25.9, 25.8, 25.7, 25.6, 24.5, 19.4. LC-MS m/z 408 $[M+Na]^+$. HRMS (EI) $[M+Na]^+$, found 408.2298. $C_{27}H_{31}NONa$ requires 408.2303.

2-{[2-(1-Hydroxy-1,2,2-trimethyl-propyl)-aziridin-1-yl]diphenyl-methyl}phenol (mixture of diastereomers) **(6d1):**

Prepared after general procedure from ketone **5d** (0.15 g, 0.41 mmol), MeLi (1.60 M solution in hexanes), 15 mL THF. Yield 0.09 g, 52% (12 h, 4 eq, 1.64 mmol, 1.03 mL MeLi; Table 3, entry 14). Title compound **6d1** was isolated by column chromatography on 100 mL silica with PE-EtOAc (10:1) eluent as a white crystalline solid, mp 135°C, R_f = 0.5 (PE-EtOAc, 5:1). IR ($CHCl_3$) 3571, 2957, 1586, 1472, 1410, 1370, 1243, 1126, 1037, 1009, 928, 819, 763. ^1H NMR (400 MHz, $CDCl_3$) δ 7.54-7.49 (6H, m, Ar), 7.30-7.16 (8H, m, Ar), 2.35 (1H, brs, OH), 1.79 (1H, unresolved dd, *J* = 3.3 Hz, C_3H), 1.57 (1H, dd, *J* = 6.6, 3.3 Hz, C_2H), 1.16 (1H, unresolved dd, *J* = 6.6 Hz, C_3H), 1.11 (3H, s, Me), 0.76 (9H, s, *t*-Bu). ^{13}C NMR (101 MHz, $CDCl_3$) δ 144.5, 129.6, 127.7, 126.9, 74.7, 74.5, 39.4, 37.6, 25.8, 25.7, 19.4. HRMS (EI) $[M+Na]^+$, found 424.2252. $C_{27}H_{31}NO_2Na$ requires 424.2252. HRMS (EI) $[M-H]^-$, found 400.2278. $C_{27}H_{30}NO_2$ requires 400.2277. Anal. Calcd for $C_{27}H_{31}NO_2$: C 80.76, H 7.78, N 3.49%. Found: C 79.71, H 7.71, N 3.42%. Crystal data: monoclinic, a = 10.1290(2), b = 21.4734(5), c = 11.2441(3) Å, β = 114.2409(10)°; V = 2229.98(9) Å^3, Z = 4, μ = 0.074 mm^{-1}, Dcalc = 1.196g·cm^{-3}; space group is $P2_1/n$. For further details, see crystallographic data deposited at the Cambridge Crystallographic Data Centre as Supplementary Publications Numbers CCDC 1868265. Copies of the data can be obtained, free of charge, on application to CCDC, 12 Union Road, Cambridge CB2 1EZ, UK.

2,2-Dimethyl-1-phenyl-(1-triphenylmethyl-aziridine-2-yl)-propane-1-ol (mixture of diastereomers) **(6e):**

Prepared after general procedure from ketone **5d** (0.15 g, 0.41 mmol), PhLi (2.00 M solution in toluene, 4 eq, 1.64 mmol, 0.82 mL), 10 mL THF. Reaction time 12 h. Yield 0.18 g, 100% (Table 3, entry 15). Title compound **6e** was isolated by column chromatography on 100 mL silica with PE-EtOAc (10:1) eluent as a white crystalline solid, mp 170°C, R_f = 0.5 (PE-EtOAc, 5:1). IR ($CHCl_3$) 3367, 3057, 2979, 2962, 2907, 2872, 1596, 1487, 1447, 1394, 1338, 1218, 1030, 986, 759, 707 cm^{-1}. ^1H NMR (400 MHz, $CDCl_3$) δ 7.57-7.45 (6H, m, Ar), 7.34-7.04 (14H, m, Ar), 4.53 (1H, s, OH), 2.13 (1H, dd, *J* = 7.0, 3.7 Hz, C_2H), 1.38 (1H, unresolved dd, *J* = 3.7 Hz, C_3H); 1.31 (1H, unresolved dd, *J* = 7.0 Hz, C_3H); 0.70 (9H, s, *t*-Bu). ^{13}C NMR (101 MHz, $CDCl_3$) δ 45.4, 143.9, 129.7, 127.7, 127.5, 127.1, 126.6, 126.0, 76.3, 74.8, 40.1, 38.4, 26.3, 25.9. HRMS (EI) $[M+H]^+$, found 448.2629. $C_{32}H_{34}NO$ requires 448.1990. Anal. Calcd for $C_{32}H_{33}NO$: C 85.87, H 7.43, N 3.13%. Found: C 85.98, H 7.17, N 2.97%.

1-Phenyl-(1-triphenylmethylaziridine-2-yl)-ethanol (mixture of diastereomers) **(6f):**

Prepared after general procedure from ketone **5e** (0.15 g, 0.39 mmol), MeLi (1.60 M solution in hexanes), 15 mL THF. Reaction time 12 h. Yield 0.14 g, 88% (1 eq, 0.39 mmol, 0.24 mL MeLi; Table 3, entry 16); 0.10 g, 61% (4 eq, 1.56 mmol, 0.96 mL MeLi; Table 3, entry 17). Title compound **6f** (0.49 g, 61%) was isolated by column chromatography on 150 mL silica with PE-EtOAc (4:1) eluent as a white crystalline solid, mp 151°C, R_f = 0.6 (PE-EtOAc, 4:1). IR ($CHCl_3$) 3446, 3057, 3030, 2979, 1596, 1495, 1447, 1329, 1218, 1091, 1061, 1020, 923, 754, 701 cm^{-1}. ^1H NMR (400 MHz, $CDCl_3$) δ 7.43-7.09 (20H, m, Ar), 3.59 (1H, s, OH), 2.03 (1H, unresolved dd, *J* =3.3 Hz, C_3H), 1.90 (1H, dd, *J* = 6.6, 3.3 Hz, C_2H), 1.37 (3H, s, Me), 1.25 (1H, unresolved dd, *J* = 6.6 Hz C_3H). ^{13}C NMR (101 MHz, $CDCl_3$) δ 148.3, 144.0, 129.4, 128.1, 127.5, 126.8, 126.6, 124.9, 73.9, 71.2, 42.6, 28.6, 23.6. HRMS (EI) $[M+H]^+$, found 406.2164. $C_{29}H_{28}NO$ requires 406.2171.

(1-*tert*-Butyloxycarbonylaziridine-2-yl)-methyl-methanone (7a):

Prepared after general procedure from amide **4b** (0.51 g, 2.38 mmol), MeLi (1.50 M solution in hexanes), 10 mL THF. Reaction time 1 h. Yield 0.27 g, 62% (1 eq, 2.38 mmol, 1.59 mL MeLi; Table 4, entry 1); 0.04 g, 8% (2 eq, 4.76 mmol, 3.18 mL MeLi; Table 4, entry 2). Title compound **7a** was isolated by column chromatography on 100 mL silica with PE-EtOAc (2:1) eluent as a colourless oil, R_f = 0.6 (PE-EtOAc, 2:1). IR ($CHCl_3$) 3422, 2981, 2934, 1733, 1368, 1331, 1300, 1159, 854 cm^{-1}. ^1H NMR (400 MHz, $CDCl_3$) δ 3.05 (1H, dd, *J* 6.0, 3.2Hz, C_2H), 2.39 (1H, dd, *J* 6.0, 1.3Hz, C_3H), 2.35 (1H, dd, *J* 3.2, 1.3Hz, C_3H), 2.15 (3H, s, Me), 1.40 (9H, s, *t*-Bu). ^{13}C NMR (101 MHz, $CDCl_3$) δ 203.6, 160.1, 82.3, 41.1, 31.8, 27.9, 27.0. LC-MS m/z 186 $[M+H]^+$. HRMS (EI) $[M+Na]^+$, found 208.0951. $C_9H_{15}NO_3Na$ requires 208.0950.

(1-*tert*-Butyloxycarbonylaziridine-2-yl)-n-butyl-methanone (7b):

Prepared after general procedure from amide **4b** (0.50 g, 2.33 mmol), *n*-BuLi (2.50 M solution in toluene, 1 eq, 2.33 mmol, 0.93 mL), 10 mL THF, reaction time 1 h. Yield 0.40 g, 75% (Table 4, entry 3). Title compound **7b** was isolated by column chromatography on 100 mL silica with PE-EtOAc (3:1) eluent as a colourless oil, R_f = 0.7 (PE-EtOAc, 3:1). IR ($CHCl_3$) 2962, 2935, 2874, 1733, 1730, 1396, 1369, 1338, 1300, 1220, 1159, 1055, 855 cm^{-1}. ^1H NMR (400 MHz, $CDCl_3$) δ 3.11-3.07 (1H, m, C_2H), 2.59-2.41 (2H, m, *n*-Bu), 2.39-2.36 (2H, m, C_3H), 1.61-1.53 (2H, m, *n*-Bu), 1.42 (9H, s, *t*-Bu), 1.37-1.25 (2H, m, *n*-Bu), 0.88 (3H, t, *J* = 7.3 Hz, *n*-Bu). ^{13}C NMR (101 MHz, $CDCl_3$) δ 205.6, 160.1, 82.1, 40.4, 32.0, 28.0, 25.6, 22.4, 13.9. LC-MS m/z 228 $[M+H]^+$. HRMS (EI) $[M+Na]^+$, found 250.1418. $C_{12}H_{21}NO_3Na$ requires 250.1419.

(1-*tert*-Butyloxycarbonylaziridine-2-yl)-*tert*-butyl-methanone (7c):

Prepared after general procedure from amide **4b** (0.58 g, 2.71 mmol), *t*-BuLi (1.50 M solution in hexanes, 1.80 mL, 2.71 mmol), 20 mL THF. Reaction time 1 h. Yield 0.50 g, 82% (Table 4, entry 4). Title compound **7c** was isolated by column chromatography on 40 mL silica with PE-EtOAc (3:1) eluent as a colourless oil, R_f = 0.7 (PE-EtOAc, 3:1). IR ($CHCl_3$) 2974, 2935, 2874, 1735, 1479, 1394, 1369, 1334, 1220, 1160, 1064, 1013, 856, 799 cm^{-1}; LC-MS m/z 228 $[M+H]^+$. ^1H NMR (400 MHz, $CDCl_3$) δ 3.41 (1H, dd, *J* = 5.1, 3.1 Hz, C_2H), 2.40 (1 H, dd, *J* = 3.1, 2.1 Hz, C_3H), 2.31 (1H, dd, *J* = 5.1, 2.1 Hz, C_3H), 1.40 (9H, s, *t*-Bu, 1.21 (9H, s, *t*-Bu). ^{13}C NMR (101 MHz, $CDCl_3$) δ 208.3, 160.0, 81.8, 44.3, 35.8, 32.8, 28.0, 25.6. HRMS (EI) $[M-H]^-$, found 226.1438. $C_{12}H_{21}NO_3$ requires 226.1443. HRMS (EI) $[M+Na]^+$, found 250.1421. $C_{12}H_{21}NO_3Na$ requires 250.1419.

(1-*tert*-Butyloxycarbonylaziridine-2-yl)-phenyl-methanone (7d):

Prepared after general procedure from amide **4b** (0.50 g, 2.33 mmol), PhLi (2.00 M solution in toluene), 20 mL THF. Yield 0.29 g, 51% (1 h, 4 eq, 9.32 mmol, 4.66 mL PhLi; Table 4, entry 6); 0.34 g, 60% (1 h, 1 eq, 2.33 mmol, 1.17 mL PhLi; Table 4, entry 5). From ester **1c** (0.50 g, 1.80 mmol), PhLi (2.00 M solution in toluene), 20 mL THF. Reaction time 1 h. Yield 0.12 g, 26% (1 eq, 1.80 mmol, 0.90 mL PhLi; Table 5, entry 1); 0.05 g, 10% (2 eq, 3.60 mmol, 1.80 mL PhLi; Table 5, entry 2); 0.04 g, 8% (4 eq, 7.20 mmol, 3.60 mL PhLi; Table 5, entry 3). From ketone **8** (0.10 g, 0.68 mmol), Boc_2O (0.15 g, 0.68 mmol), 5 mL CH_2Cl_2, RT, reaction time 1 h. Yield 0.13 g, 78%. Title compound **7d** was isolated by column chromatography on 300 mL silica with PE-EtOAc (1:1) eluent as a white crystalline solid, mp 55°C. R_f = 0.5 (PE-EtOAc, 2:1). IR ($CHCl_3$) 3066, 2979, 2934, 1733, 1683, 1598, 1450, 1370, 1338, 1310, 1231, 1158, 1018, 851, 710 cm^{-1}. ^1H NMR (400 MHz, $CDCl_3$) δ 8.11-8.07 (2H, m, Ar), 7.65-7.60 (1H, m, Ar), 7.55-7.49 (2H, m, Ar), 3.93 (1H, dd, *J* = 5.3, 3.3 Hz, C_2H), 2.70 (1H, dd, *J* = 3.3, 1.5 Hz, C_3H), 2.53 (1H, dd, *J* = 5.3, 1.5 Hz, C_3H), 1.44 (9H, s, *t*-Bu). ^{13}C NMR (101 MHz, $CDCl_3$) δ 193.8, 160.4, 136.7, 134.0, 128.9, 128.7, 82.2, 37.6, 32.7, 28.0. HRMS (EI) $[2M+Na]^+$, found 517.2321. $C_{28}H_{34}N_2O_6Na$ requires 517.2315. Anal. Calcd for $C_{14}H_{17}NO_3$: C 68.00, H 6.93, N 5.66%. Found: C 68.01, H 6.90, N 5.54%.

Aziridine-2-yl-phenyl-methanone (8) [20]:

Prepared after general procedure from amide **4b** (0.50 g, 2.33 mmol), PhLi (2.00 M solution in toluene), 20 mL THF. Reaction time 1 h. Yield 0.22 g, 66% (4 eq, 9.32 mmol, 1.17 mL PhLi, warmed to RT; Table 3, entry 7); 0.12 g, 35% (4 eq, 9.32 mmol, 4.68 mL PhLi; Table 4, entry 6). Title compound **8** was isolated by column chromatography on 300 mL silica with PE-EtOAc (1:1) eluent as a colourless oil, R_f = 0.2 (PE-EtOAc, 1:1). ^1H NMR (400 MHz, $CDCl_3$) δ 8.09-7.98 (2H, m, Ar), 7.69-7.20 (3H, m, Ar), 3.47 (1H, dd, *J* = 5.7, 2.9 Hz, C_2H), 2.02 (1H, br s, NH), 2.04 (1H, dd, *J* = 5.7, 1.4 Hz, C_3H), 1.97-1.86 (1H, m, C_3H). LC-MS m/z 170 $[M+Na]^+$.

Diphenyl-(1-*tert*-butyloxycarbonylaziridine-2-yl)-methanol (9):

Prepared after general procedure from ester **1c** (0.50 g, 2.33 mmol), PhLi (2.00 M solution in toluene), 20 mL THF. Yield 0.03 g, 5% (1 h, 1 eq, 2.33 mmol, 1.17 mL PhLi; Table 4, entry 1); 0.24 g, 40% (1 h, 2 eq, 4.66 mmol, 2.34 mL PhLi; Table 4, entry 2); 0.30 g, 52% (1 h, 4 eq, 9.32 mmol, 4.68 mL PhLi; Table 4, entry 3); 0.03 g, 5% (12 h, 4 eq, 9.32 mmol, 4.68 mL PhLi; Table 4, entry 4); 0.01 g, 2% (12 h, 4 eq, 9.32 mmol, 4.68 mL PhLi; warmed to RT before quenching; Table 4, entry 5). From ketone **7a** (0.40 g, 1.62 mmol), PhLi (2.00 M solution in toluene, 0.81 mL, 1.62 mmol), 10 mL THF. Reaction time 1 h. Yield 0.32 g, 61%. Title compound **9** was isolated by column chromatography on 150 mL silica with PE-EtOAc (4:1) eluent as a white crystalline solid, mp 111°C, R_f = 0.6 (PE-EtOAc, 4:1). IR ($CHCl_3$) 3510, 3063, 3027, 2979, 2931, 1716, 1448, 1369, 1308, 1160, 699 cm^{-1}. ^1H NMR (400 MHz, $CDCl_3$) δ 7.70-7.65 (2H, m, Ar), 7.40-7.23 (8H, m, Ar), 3.38 (1H, dd, *J* = 6.3, 3.7 Hz, C_2H), 2.77 (1H, s, OH); 2.36 (1H, unresolved dd, *J* = 3.7 Hz, C_3H), 2.26 (1H, unresolved dd, *J* = 6.3 Hz, C_3H), 1.45 (9H, s, *t*-Bu). ^{13}C NMR (101 MHz, $CDCl_3$) δ 162.2, 146.0, 144.2, 128.3, 127.4, 126.3, 82.1, 75.0, 44.3, 28.3, 28.0. LC-MS m/z 348 $[M+Na]^+$. Anal. Calcd for $C_{20}H_{23}NO_3$: C 73.82, H 7.12, N 4.30%. Found: C 73.34, H 7.04, N 4.21%.

Aziridine-2-yl-diphenyl-methanol (10):

Prepared after general procedure from ester **1c** (0.50 g, 2.33 mmol), PhLi (2.00 M solution in toluene, 4 eq, 9.32 mmol, 4.66 mL), 20 mL THF. Reaction time 12 h. Yield 0.09 g, 22% (Table 5, entry 4); 0.06 g, 15% (warmed to RT, table 5, entry 5). From carbinol **9** (0.10 g, 0.29 mmol), PhLi (2.00 M solution in toluene, 4 eq, 1.16 mmol, 0.58 mL), 10 mL THF. Reaction time 1 h. Yield 0.03 g, 44%. Title compound **10** was isolated by column chromatography on 20 mL silica with PE-EtOAc (4:1) eluent as a white crystalline solid, mp 135°C, R_f = 0.2 (PE-EtOAc, 4:1). IR ($CHCl_3$) 3265, 3057, 3031, 2756, 1490, 1448,1195, 863, 771, 751, 700 cm^{-1}. ^1H NMR (400 MHz, $CDCl_3$) δ 7.48-7.40 (4H, m, Ar), 7.36-7.20 (6H, m, Ar), 3.51 (1H, br s, OH); 2.97-2.87 (1H, m, C_2H), 1.88 (1H, unresolved dd, *J* = 5.8 Hz, C_3H), 1.74 (1H, unresolved dd, *J* = 3.6 Hz, C_3H), 1.56 (1H, br s, NH). ^{13}C NMR (101 MHz, $CDCl_3$) δ 128.4, 128.3, 127.3, 126.7, 126.5. LC-MS m/z 248 $[M+Na]^+$. Anal. Calcd for $C_{15}H_{15}NO$: C 79.97, H 6.71, N 6.22%. Found: C 79.64, H 6.72, N 5.99%.

(1-Benzyl-2-oxo-2-phenyl-ethyl) carbaminic acid *tert*-butyl ester (11) [21]:

Prepared after general procedure from ester **1c** (0.50 g, 1.80 mmol), PhLi (2.00 M solution in toluene, 7.20 mmol, 3.60 mL), 20 mL THF. Reaction mixture was stirred for 12 h at -78°C, then warmed to the room temperature. Yield 0.05 g, 9%. Title compound **11** was isolated by column chromatography on 200 mL silica with PE-EtOAc (7:1) eluent as a white amorphous solid, R_f = 0.3 (PE-EtOAc, 1:1). ^{1}H NMR (400 MHz, $CDCl_3$) δ 7.46-7.23 (10H, m, Ar), 4.80-4.59 (1H, m, NH); 3.60 (1H, dd, *J* 6.9, 4.3Hz, C_2H), 3.53-3.32 (1H, m, C_3H), 2.90-2.71 (1H, m, C_3H), 1.43 (9H, s). LC-MS m/z 348 $[M+Na]^+$.

References

1. Singh, G.S. Advances in the synthesis and chemistry of aziridines. *Adv. Heterocyc. Chem.*, **2019**, *129*, 245-335. DOI: 10.1016/bs.aihch.2018.12.003
2. Macha, L.; D'hooghe, M.; Ha, H-J. Deployment of Aziridines for the Synthesis of Alkaloids and Their Derivatives. *Synthesis*, **2019**, *51*, 1491-1515. DOI: 10.1055/s-0037-1611715
3. Pellissier, H. Recent Developments in Asymmetric Aziridination. *Adv. Synth. Catal.*, **2014**, *356*, 1899-1935. DOI: 10.1002/adsc.201400312
4. Strumfs, B.**;** Uljanovs, R.; Velikijs, K.; Trapencieris, P.; Strumfa, I. , 3-Arylaziridine-2-carboxylic Acid Derivatives and (3-Arylaziridin-2-yl)ketones: The Aziridination Approaches. *Int. J. Mol. Sci.* **2021**, *22*(18), 9861- 9904. DOI: 10.3390/ijms22189861
5. Zhou, Z.; Kürti, L. Electrophilic Amination: An Update. *Synlett*, **2019**, *30*, 1525-1535. DOI: 10.1055/s-0037-1611861
6. Degennaro, L.; Trinchera, P.; Luisi, R. Recent Advances in the Stereoselective Synthesis of Aziridines. *Chem. Rev.*, **2014**, *114*, 7881-7929. DOI: 10.1021/cr400553c
7. Yudin, A.K., Ed. Aziridines and Epoxides in Organic Synthesis. Weinheim: Wiley-VCH, **2006**, 492 pp. DOI: 10.1002/3527607862
8. Shtrumfs, B.; Hermane, J.; Kalvinsh, I.; Trapencieris, P. Unnatural amino acids. 3. Aziridinyl ketones from esters and amides of aziridine-2-carboxylic acid. *Chem. Heterocycl. Comp.*, **2007**, *43*, 169-174. DOI: 10.1007/s10593-007-0028-7
9. Adler, M.; Adler, S.; Boche, G. J. Tetrahedral Intermediates in Reactions of Carboxylic Acid Derivatives with Nucleophiles. Phys. Org. Chem., **2005**, *18*, 193-209. DOI: 10.1002/poc.807
10. Deyrup, J.A.; Moyer, C.L. Aziridinemethanols. *J. Org. Chem.*, **1970**, *35*, 3424-3428. DOI: 10.1021/jo00835a052
11. Yu, L.; Kokai, A.; Yudin, A.K. Preparation and Reactivity of Versatile α-Amino Ketones. *J. Org. Chem.*, **2007**, *72*, 1737-1741. DOI: 10.1021/jo062401o
12. Yun, J.M.; Sim, T.B.; Hahm, H.S.; Lee. W.K. Efficient Synthesis of Enantiomerically Pure 2-Acylaziridines: Facile Syntheses of *N*-Boc-safingol, *N*-Boc-d-erythro-sphinganine, and *N*-Boc-spisulosine from a Common Intermediate. *J. Org. Chem.*, **2003**, *68*, 7675-7680. DOI: 10.1021/jo034755a
13. Woydowski, K.; Liebscher, J. Synthesis of Optically Active 3-Amino-2,3-dihydrobenzopyran-4-ones by Ring Transformation of Aziridinecarboxamides. *Synthesis*, **2000**, 1444-1448. DOI: 10.1055/s-2000-7101
14. Rubin, A.E.; Sharpless, K.B. A Highly Efficient Aminohydroxylation Process. *Angew Chem Int Ed*, **1997**, *36*, 2637-2640. DOI: 10.1002/anie.199726371
15. Molander, G.A.; Stengel, P.J. Reduction of 2-acylaziridines by samarium (II) iodide. An efficient and regioselective route to β-amino carbonyl compounds. *Tetrahedron*, **1997**, *53*, 8887-8912. DOI: 10.1016/S0040-4020(97)90399-9
16. Sureshbabu, P.; Azeez, S.; Muniyappan, N.; Sabiah, S.; Kandasamy, J. Chemoselective Synthesis of Aryl Ketones from Amides and Grignard Reagents via C(O)-N Bond Cleavage under Catalyst-Free Conditions. *J Org Chem*, **2019**, *84*, 11823-11838. DOI: 10.1021/acs.joc.9b01699
17. Chan, G.H.; Ong, D.Y.; Yen, Z.; Chiba, S. Reduction of *N,N*-Dimethylcarboxamides to Aldehydes by Sodium Hydride–Iodide Composite. *Helv Chem Acta*, **2018**, *101*, e1800049. DOI: 10.1002/hlca.201800049
18. Anthore-Dalion, L.; Benischke, A.D.; Wei, B.; Berionni, G.; Knochel, P. The Halogen-Samarium Exchange Reaction: Synthetic Applications and Kinetics. *Angew Chem Int Ed*, **2019**, *58*, 4046-4050. DOI: 10.1002/anie.201814373
19. Nadano, R.; Fuchibe, K.; Ikeda, M.; Takahashi, H.; Ichikawa, J. Rapid and Slow Generation of 1-Trifluoromethylvinyllithium: Syntheses and Applications of CF3-Containing Allylic Alcohols, Allylic Amines, and Vinyl Ketones. *Chem Asian J*, **2010**, *5*, 1875-1883. DOI: 10.1002/asia.201000139
20. Liu, Z.; Liu, J.; Zhang, L.; Liao, P.; Song, J.; Bi, X. Silver(I)-catalyzed hydroazidation of ethynyl carbinols: synthesis of 2-azidoallyl alcohols. *Angew. Chem., Int. Ed. Engl.* **2014**, *53*, 5305-5309. DOI: 10.1002/anie.201310264

21. Yang, H.; Li, H.; Wittenberg, R.; Egi, M.; Huang, W.; Liebeskind, L.S. Ambient Temperature Synthesis of High Enantiopurity *N*-Protected Peptidyl Ketones by Peptidyl Thiol Ester−Boronic Acid Cross-Coupling. *J. Am. Chem. Soc.* **2007**, *129*, 1132-1140. DOI: 10.1021/ja0658719
22. Strumfs, B.; Hermane, J.; Belyakov, S.; Trapencieris, P. Acyl migration from N to C in aziridine-2-carboxylic esters. *Tetrahedron,* **2014**, *70*, 355-362. DOI: 10.1016/j.tet.2013.11.052
23. Shtrumfs, B.; Chernyak D.; Kums, I.; Kalvins, I.; Trapencieris, P. Unnatural amino acids. 2. Simple method for obtaining esters of aziridine-2-carboxylic acids by transesterification reaction. *Chem. Heterocycl. Comp.* **2004**, *40*, 725-733. DOI: 10.1023/B:COHC.0000040767.96090.cc
24. Ivanova, J.; Štrumfs, B.; Žalubovskis, R. Access to NH-aziridine-2-carboxamides through Davidsen acylimidodicarbonate activation. *Comptes Rendus Chimie*, **2019**, *22*, 283-293. DOI: 10.1016/j.crci.2019.03.001
25. Altomare, A.; Burla, M.C.; Camalli, M.; Cascarano, G.L.; Giacovazzo, C.; Guagliardi, A.; Moliterni, A.G.G.; Polidori, A.; Spagna, R. *SIR97*: a new tool for crystal structure determination and refinement. *J. Appl. Crystallogr.* **1999**, *32*, 115-119. DOI: 10.1107/S0021889898007717
26. Sheldrick, G.M. A Short History of SHELX. *Acta Crystallogr.* **2008**, *A64*, 112-122. DOI: 10.1107/S0108767307043931-*tert*-Butyloxycarbonylaziridine-2-carboxylic acid methyl ester 1a

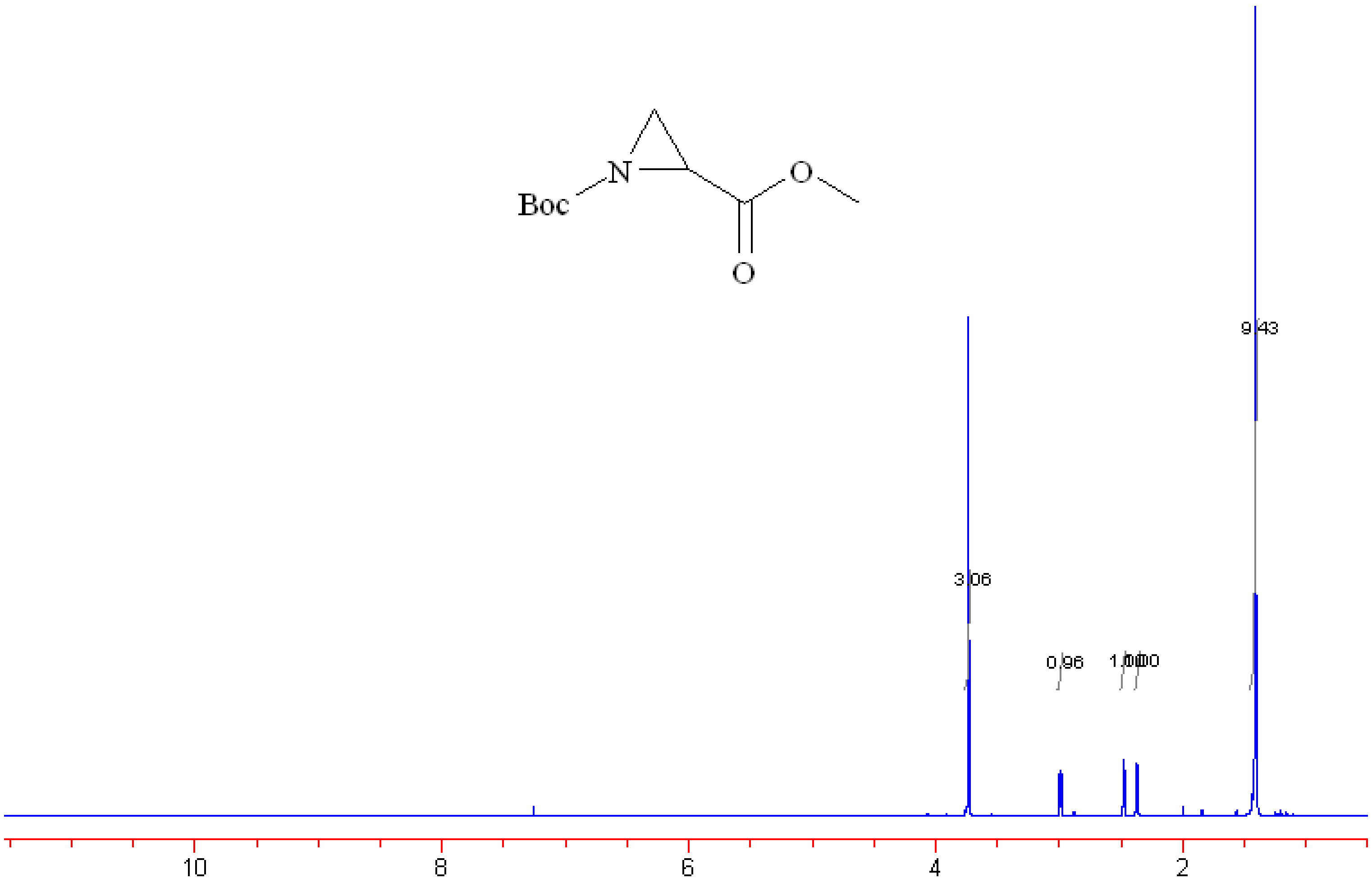

1-*tert*-Butyloxycarbonylaziridine-2-carboxylic acid methyl ester 1a

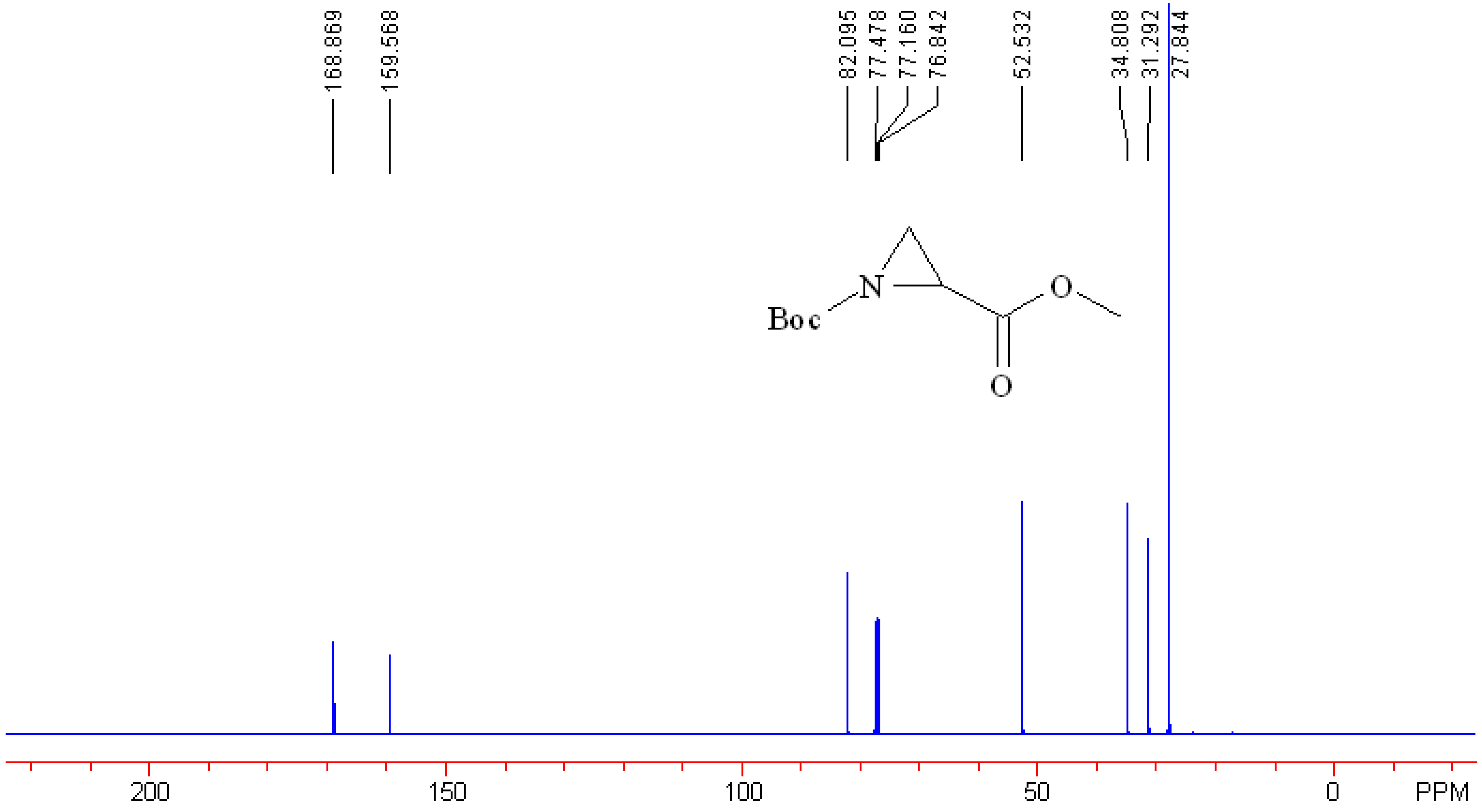

1-*tert*-Butyloxycarbonylaziridine-2-carboxylic acid methyl ester 1a

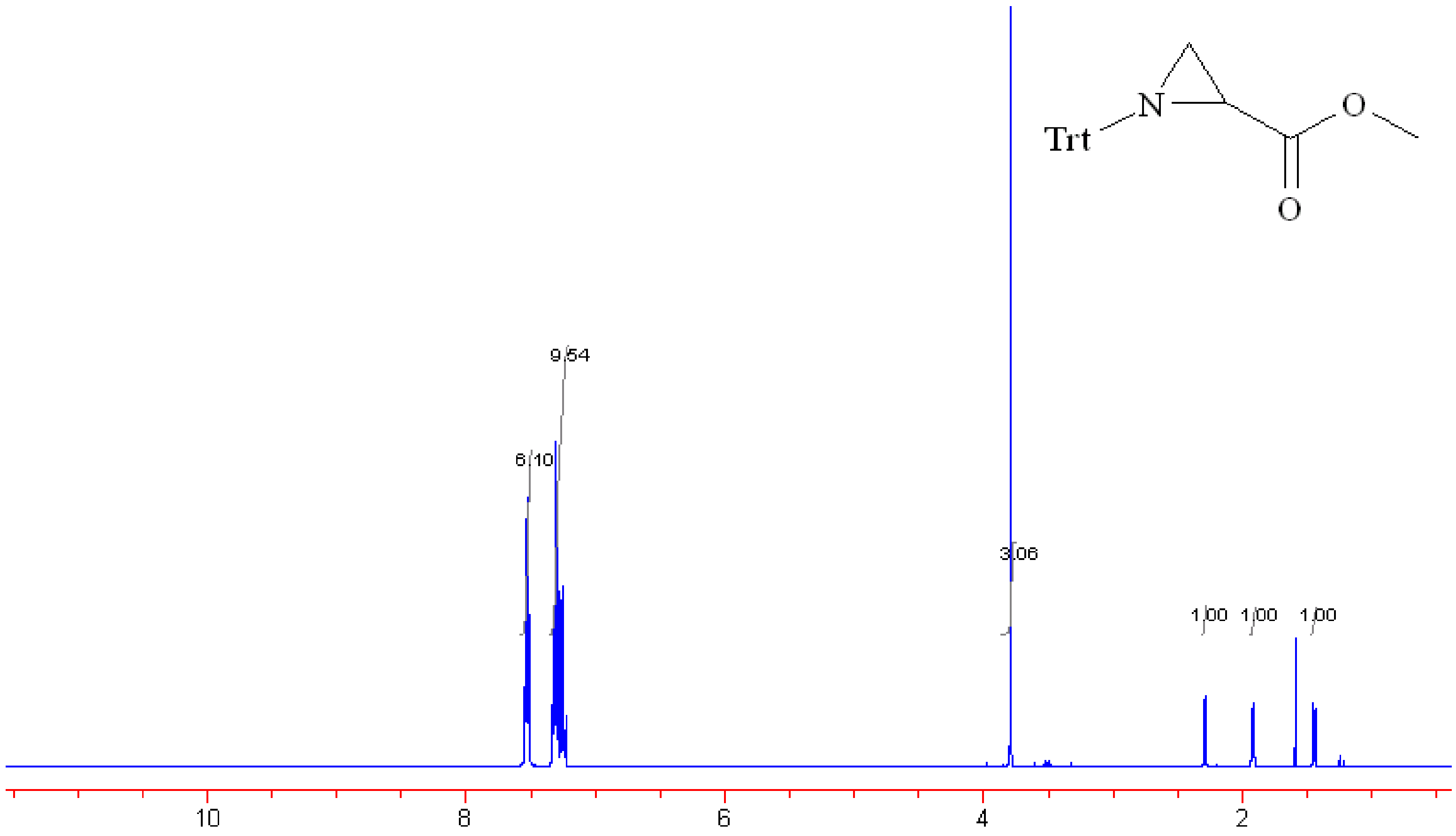

1-Trityl-aziridine-2-carboxylic acid methyl ester 1b

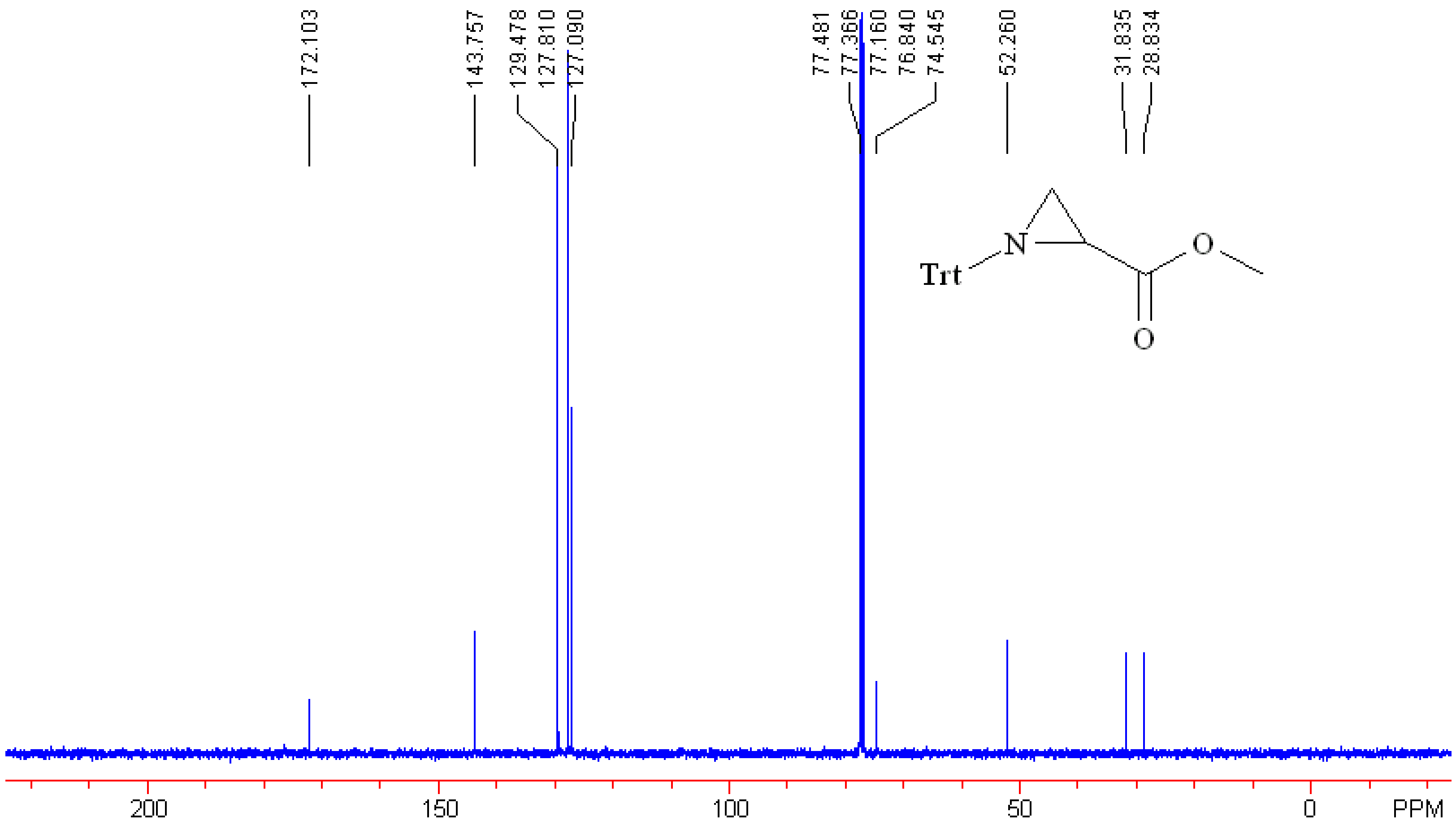

1-Trityl-aziridine-2-carboxylic acid methyl ester 1b

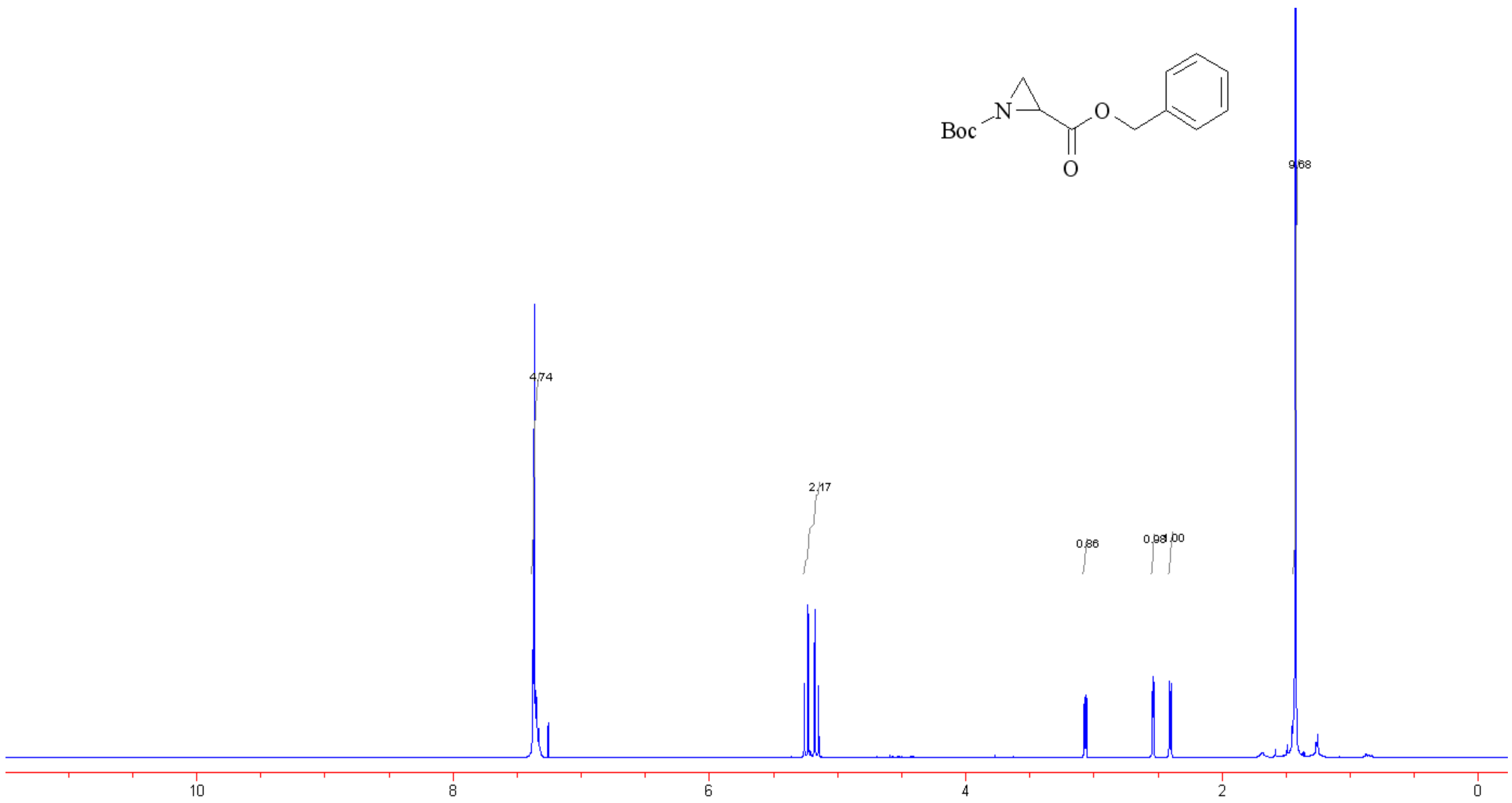

Aziridine-1,2-dicarboxylic acid 2-benzyl ester 1-*tert*-butyl ester 1c

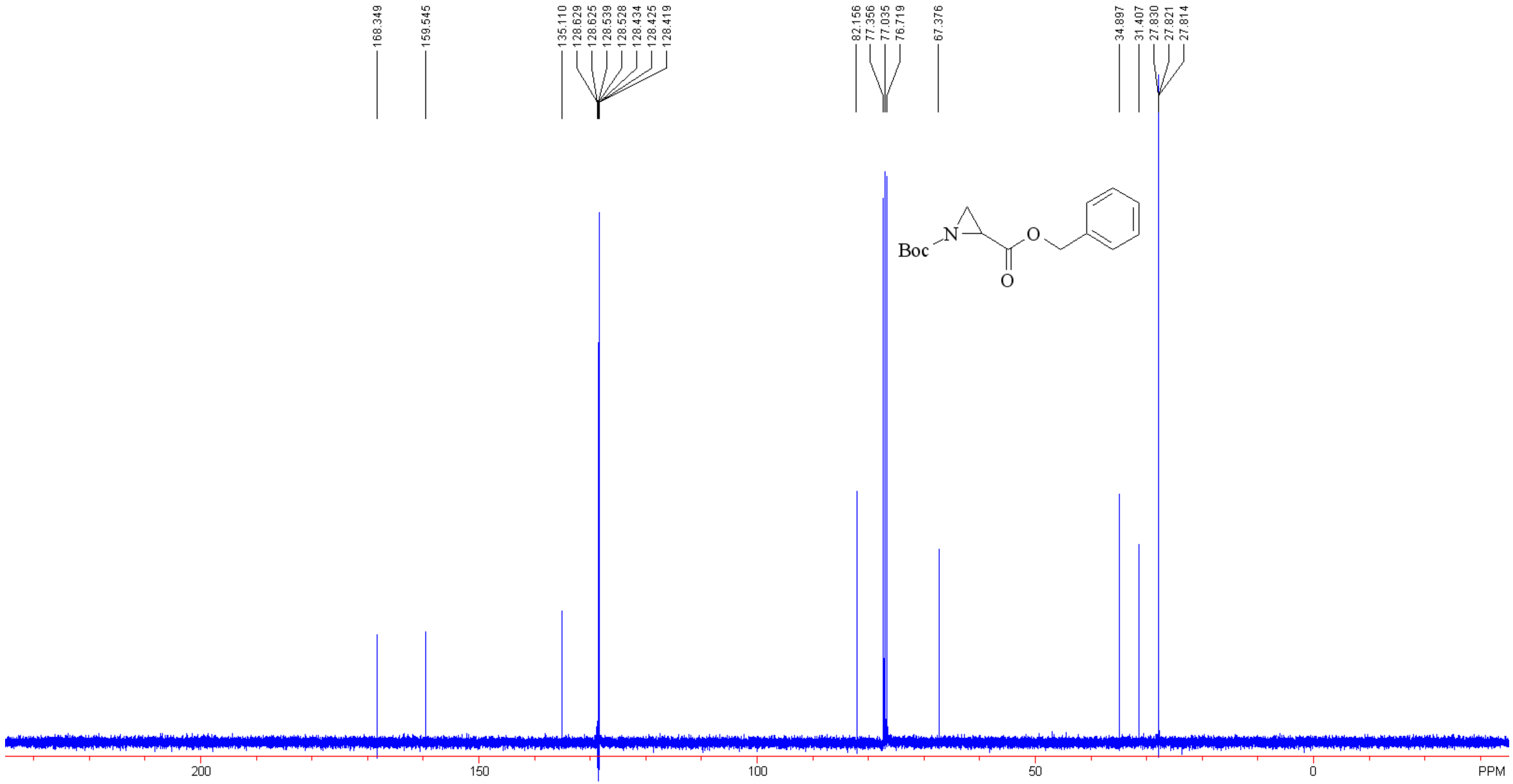

Aziridine-1,2-dicarboxylic acid 2-benzyl ester 1-*tert*-butyl ester 1c

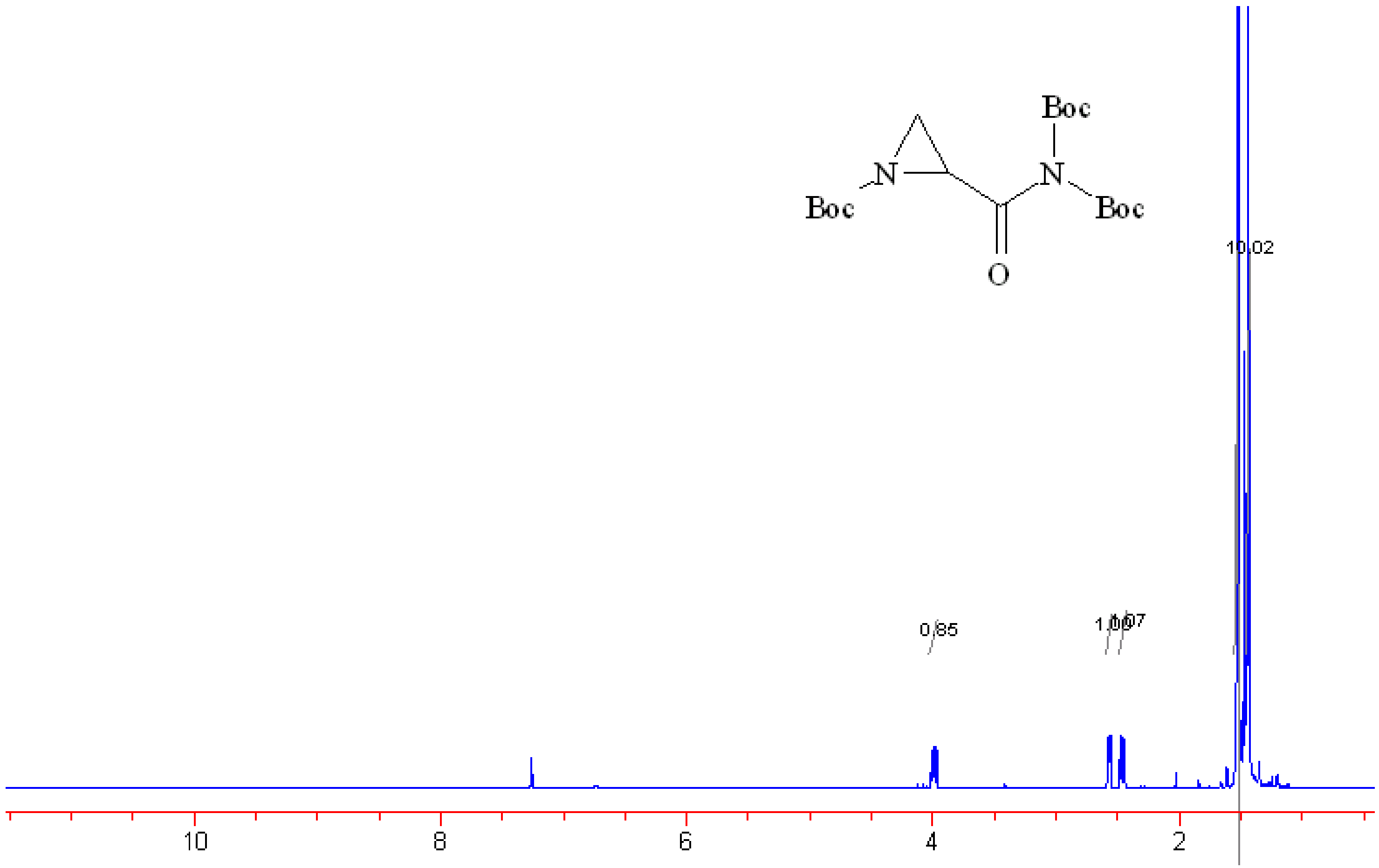

***tert*-Butyl 2-(bis(*tert*-butoxycarbonyl)carbamoyl)aziridine-1-carboxylate 2a**

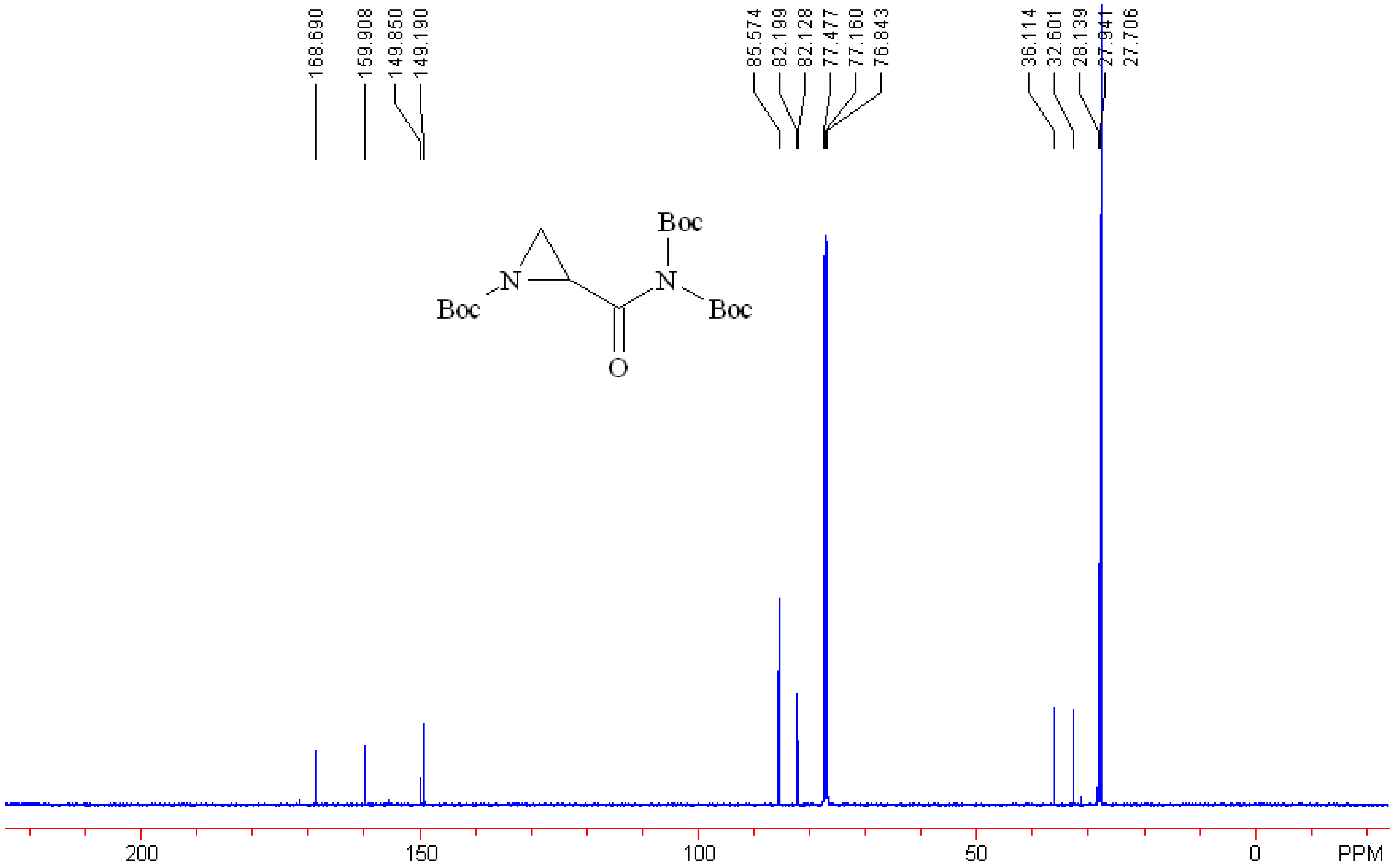

***tert*-Butyl 2-(bis(*tert*-butoxycarbonyl)carbamoyl)aziridine-1-carboxylate 2a**

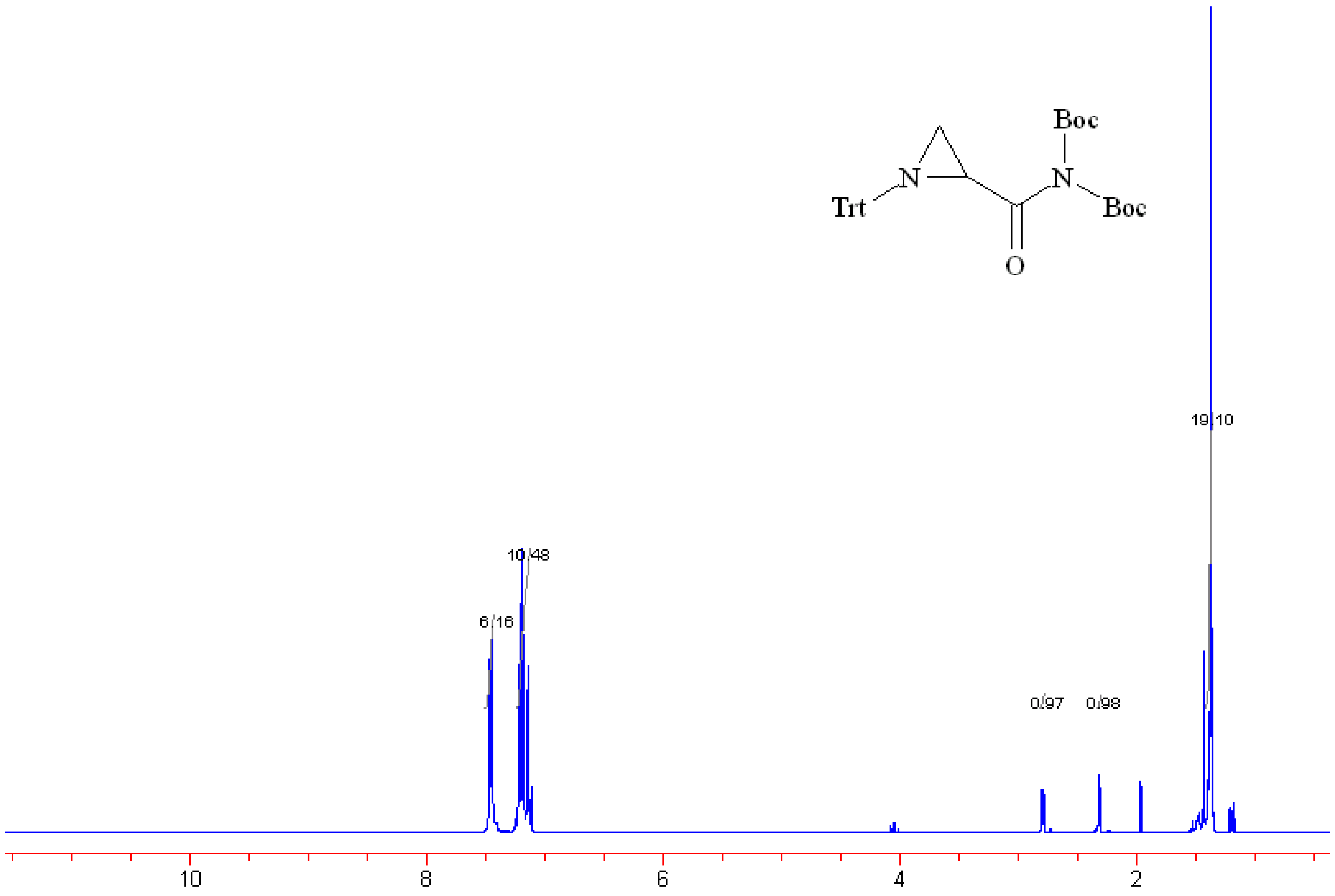

Boc
N
Trt
N
Boc
O
6.16
10.48
0.97
0.98
19.10
10
8
6
4
2

***tert*-Butyl (*tert*-butoxycarbonyl)(1-tritylaziridine-2-carbonyl)carbamate 2b**

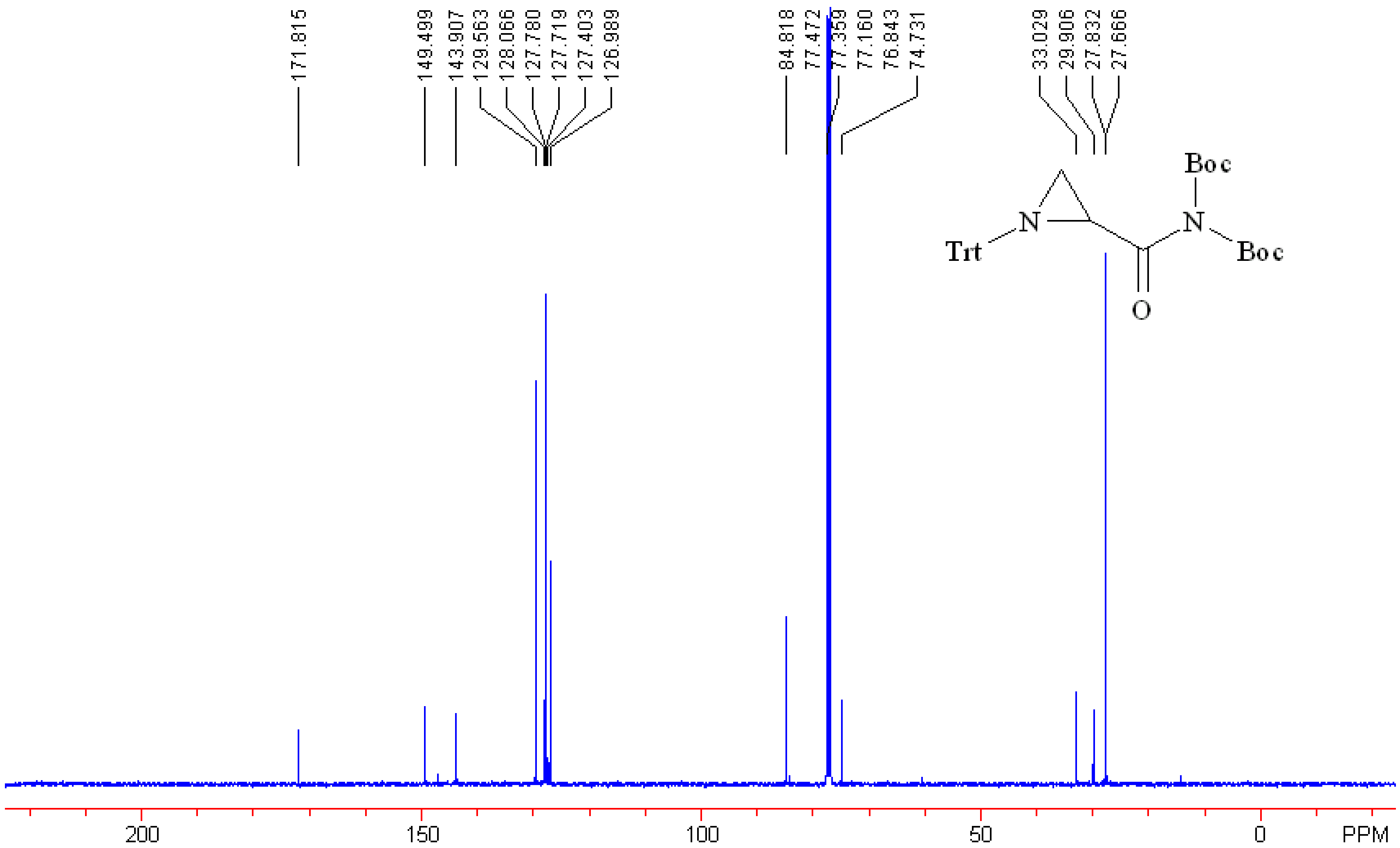

***tert*-Butyl (*tert*-butoxycarbonyl)(1-tritylaziridine-2-carbonyl)carbamate 2b**

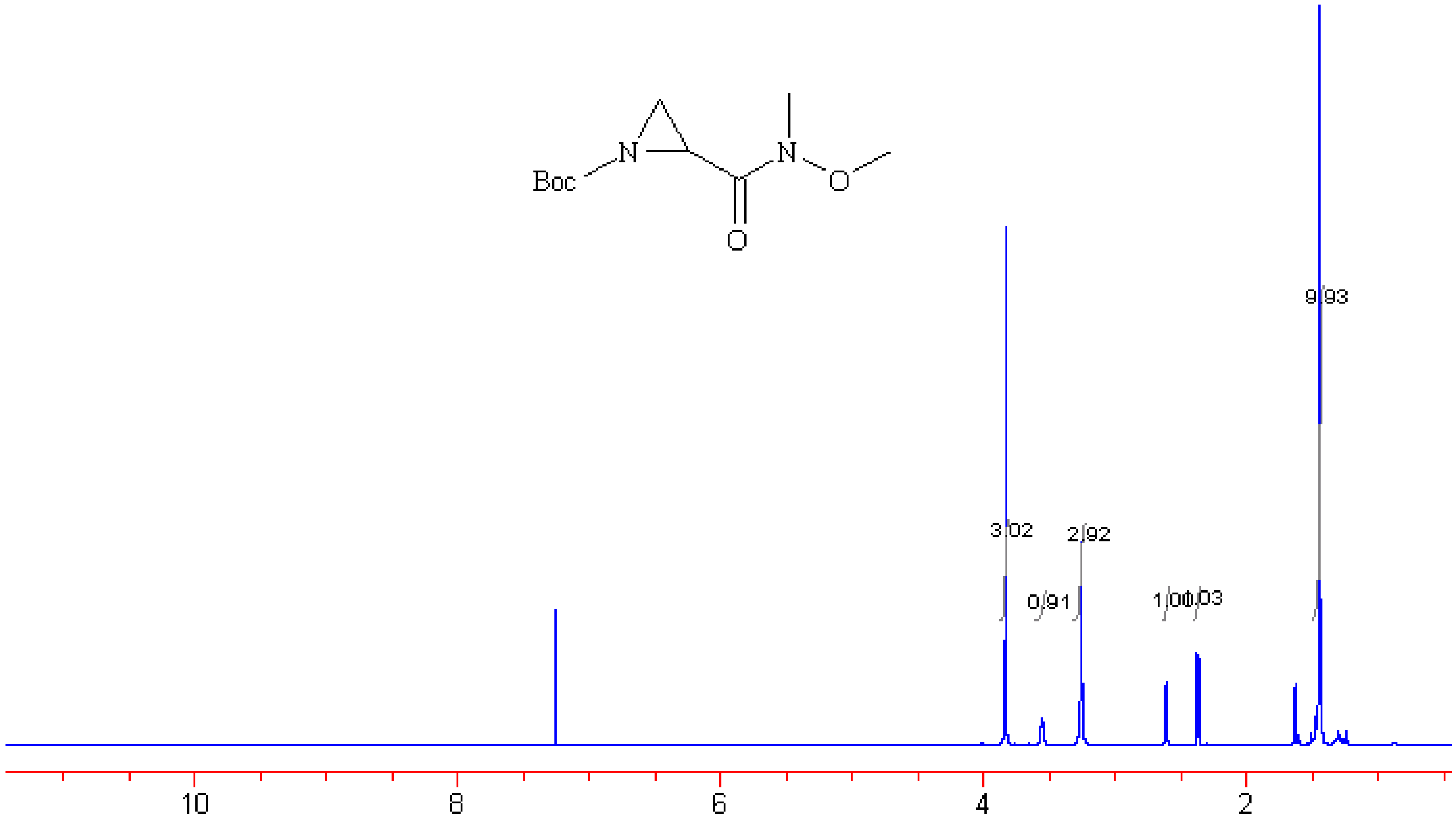

***tert*-Butyl 2-(methoxy(methyl)carbamoyl)aziridine-1-carboxylate 3b**

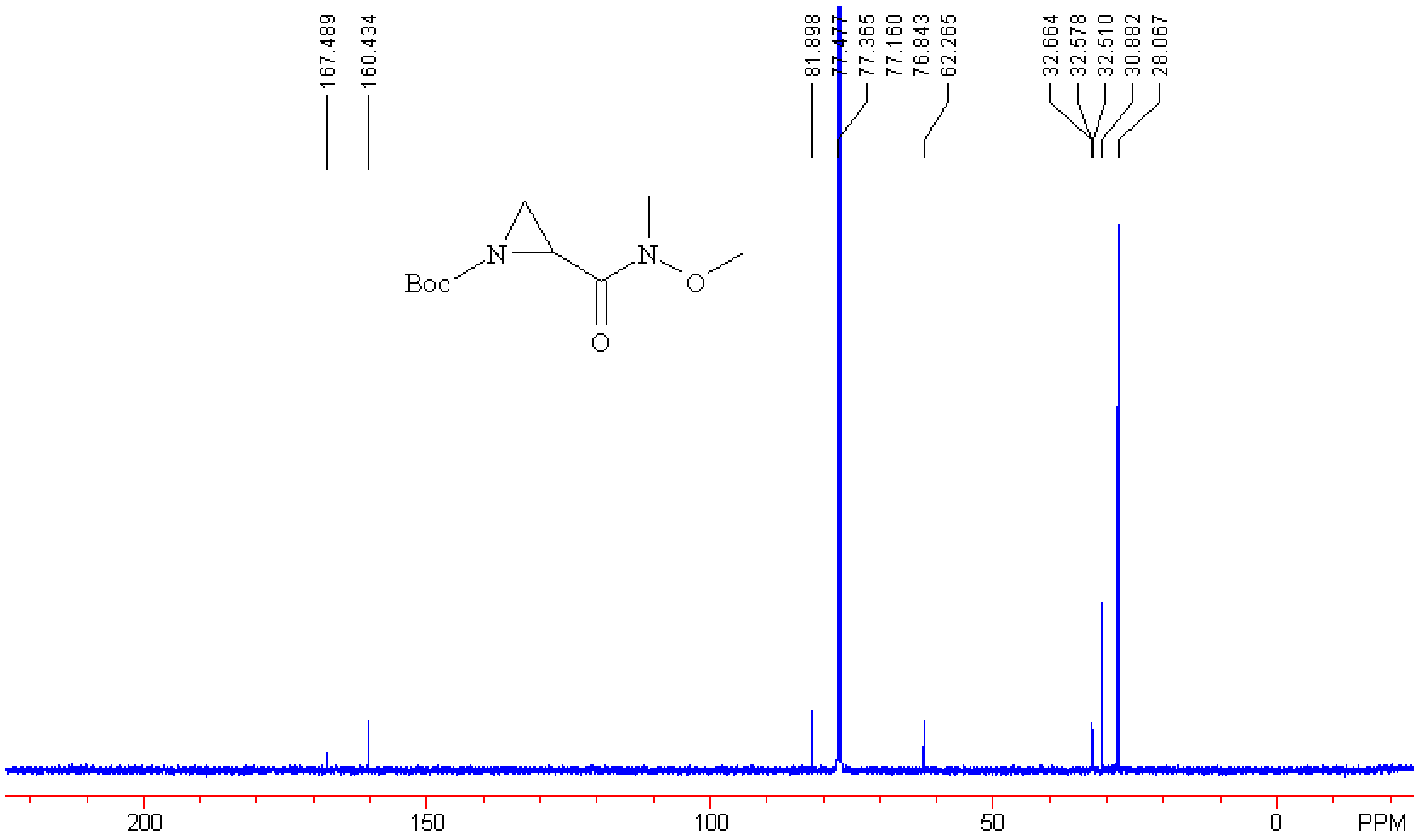

***tert*-Butyl 2-(methoxy(methyl)carbamoyl)aziridine-1-carboxylate 3b**

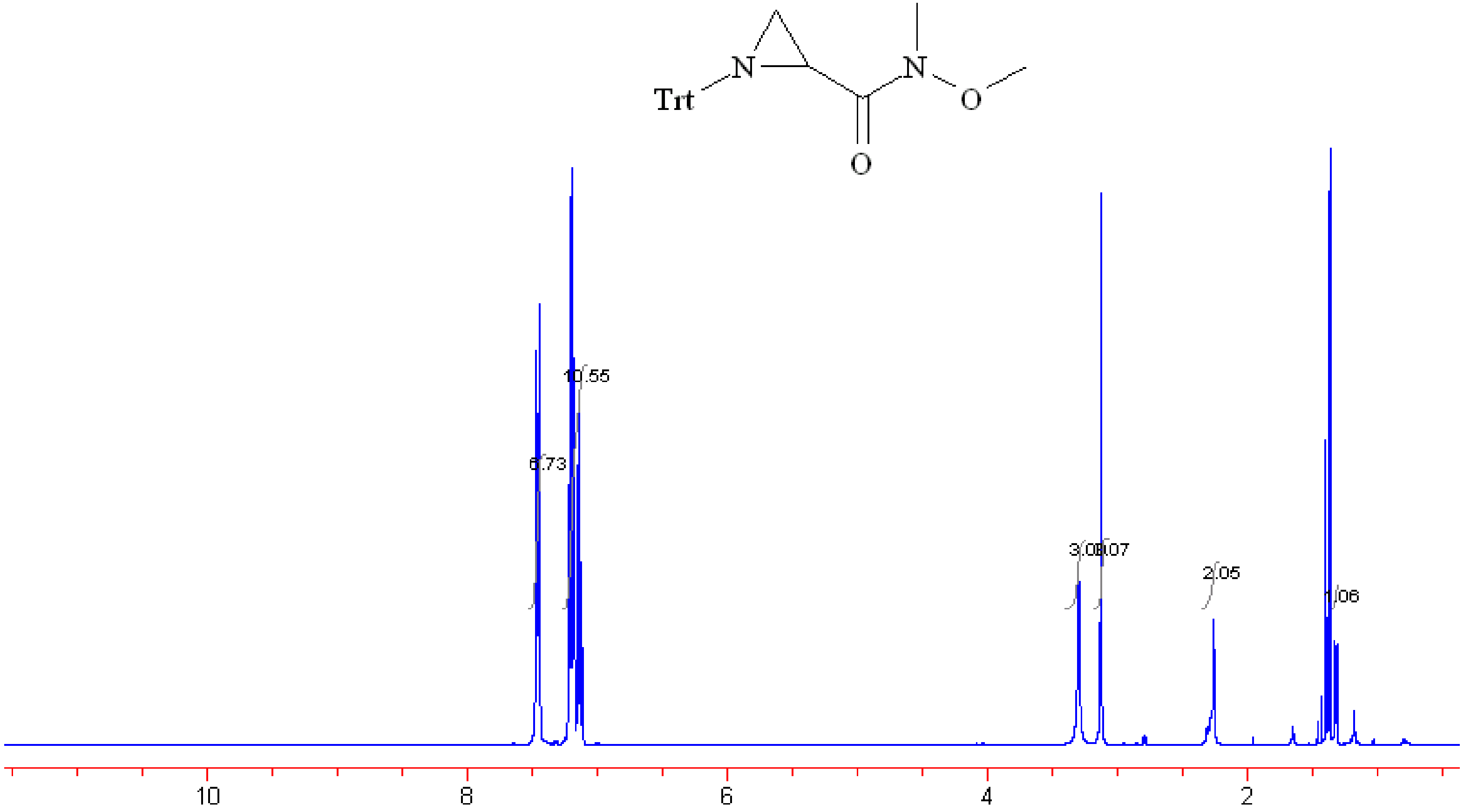

N-Methoxy-N-methyl-1-tritylaziridine-2-carboxamide 3c

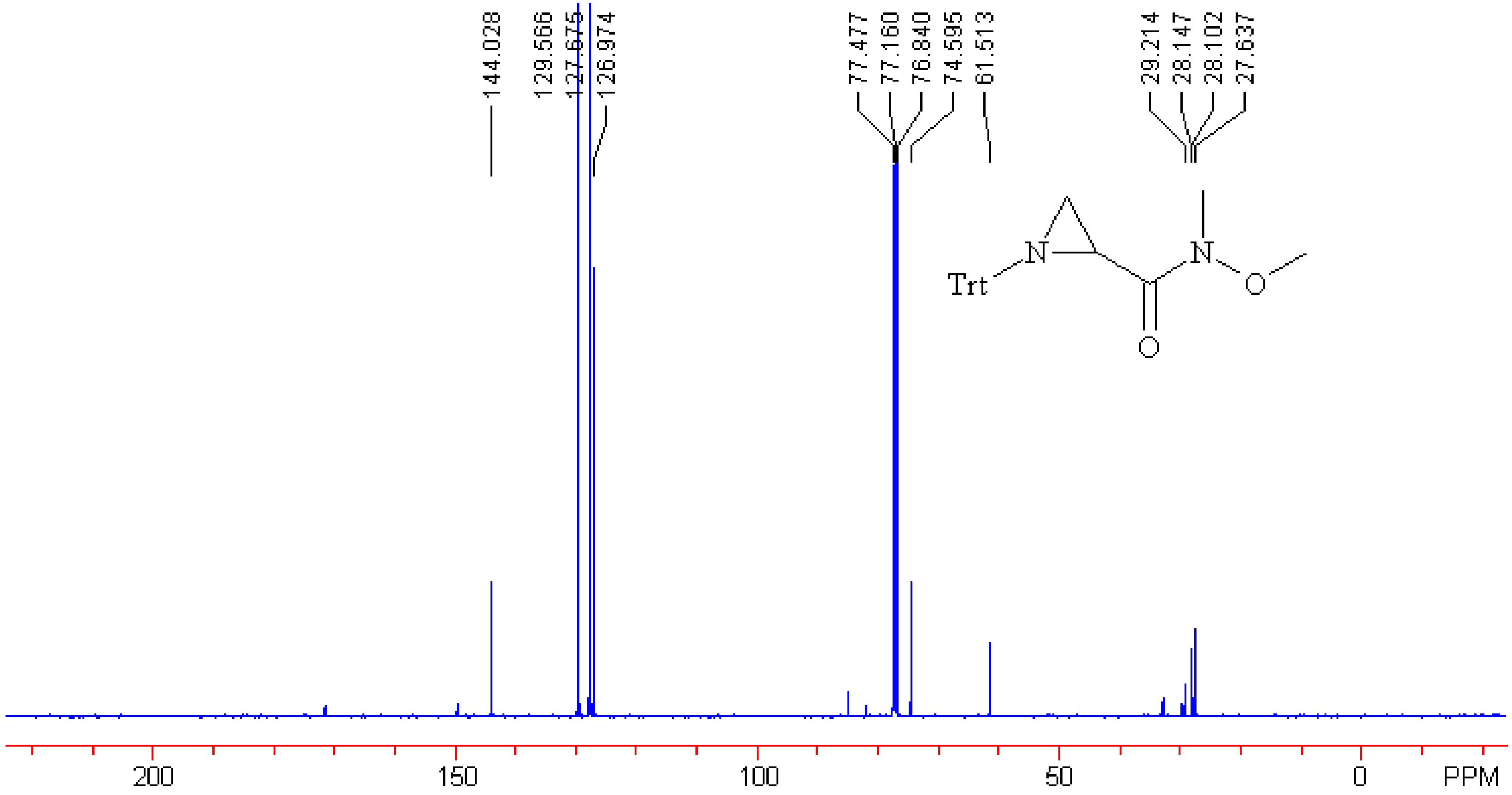

N-Methoxy-N-methyl-1-tritylaziridine-2-carboxamide 3c

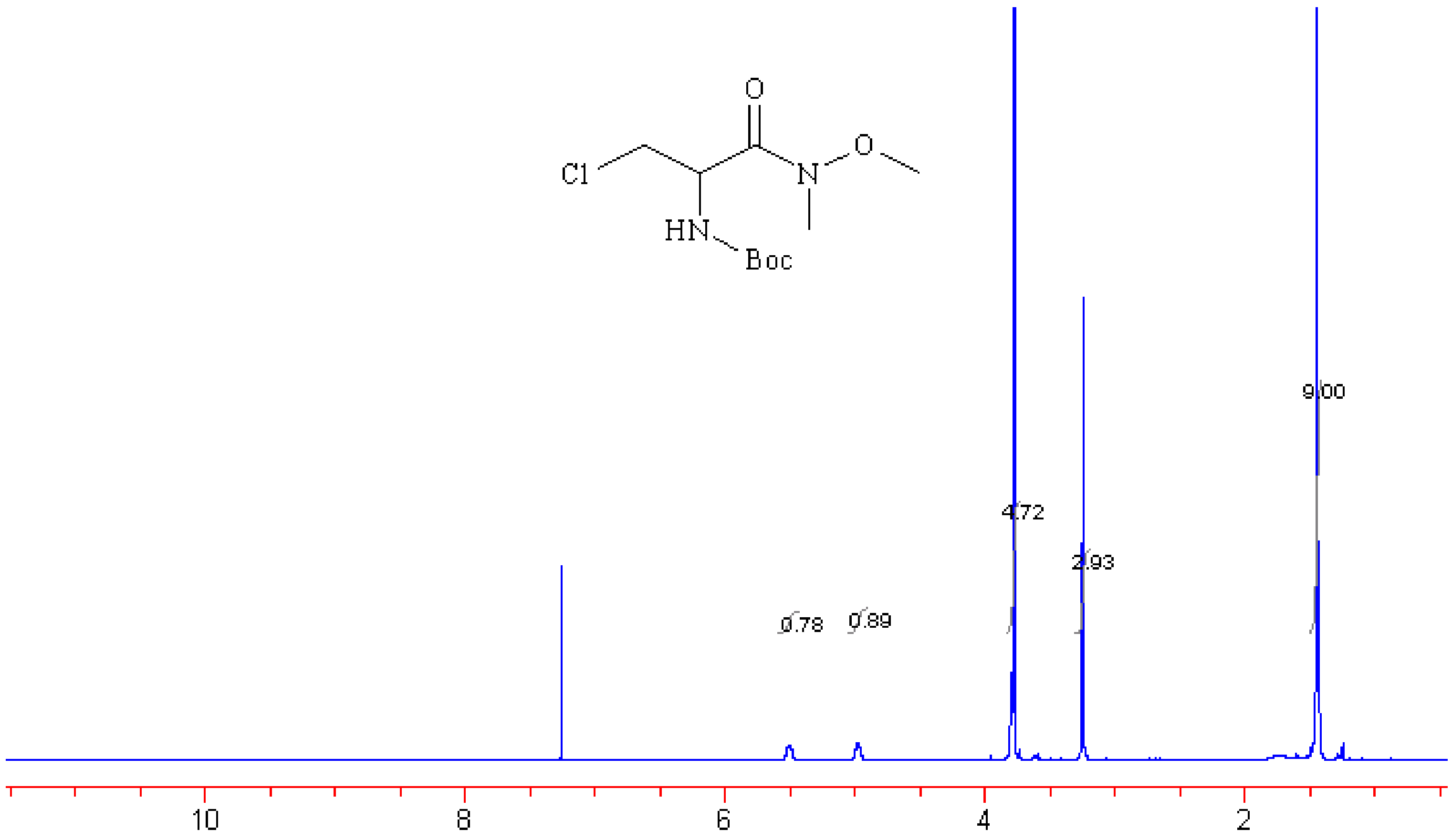

***tert*-Butyl (3-chloro-1-(methoxy(methyl)amino)-1-oxopropan-2-yl)carbamate 3d**

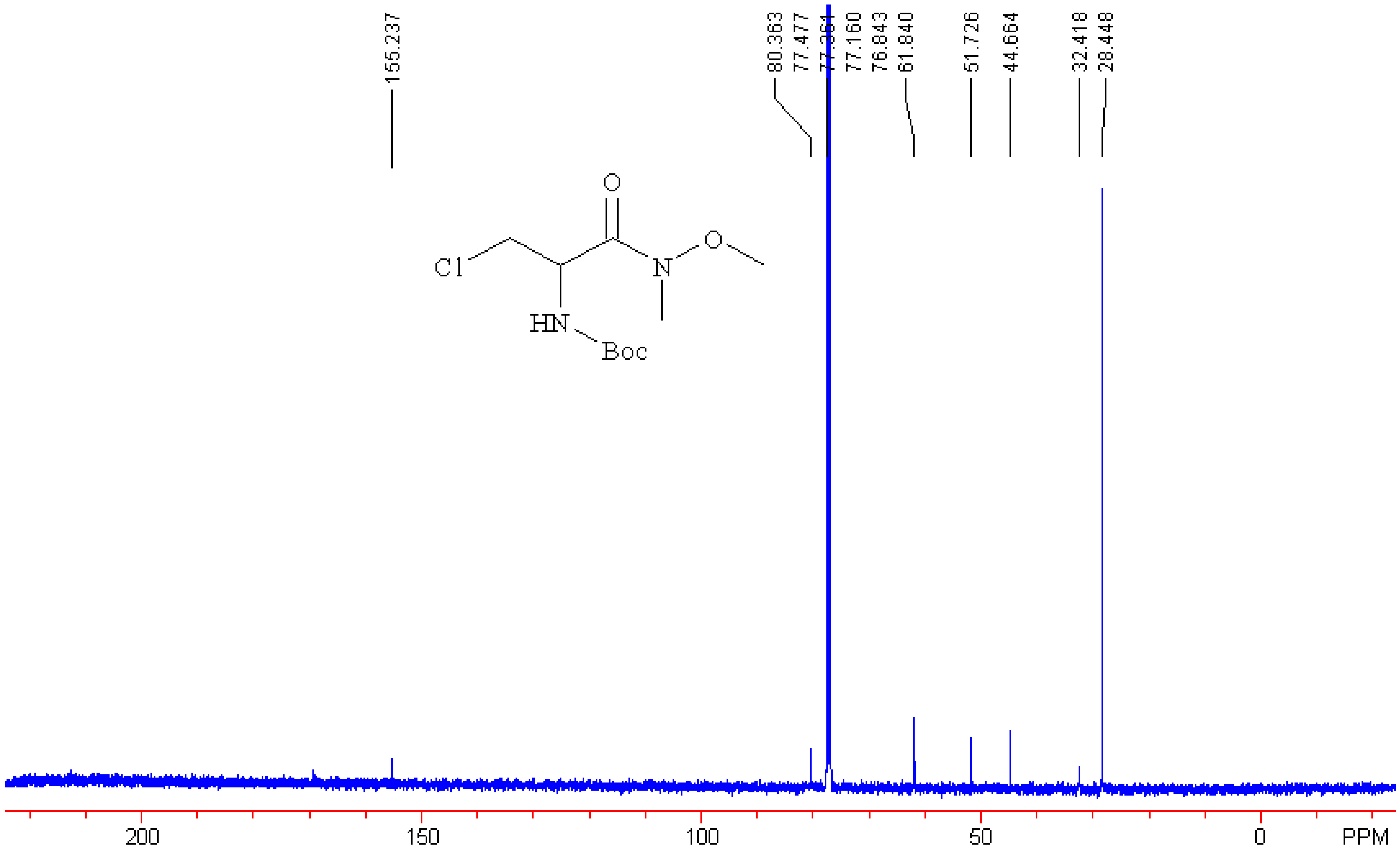

***tert*-Butyl (3-chloro-1-(methoxy(methyl)amino)-1-oxopropan-2-yl)carbamate 3d**

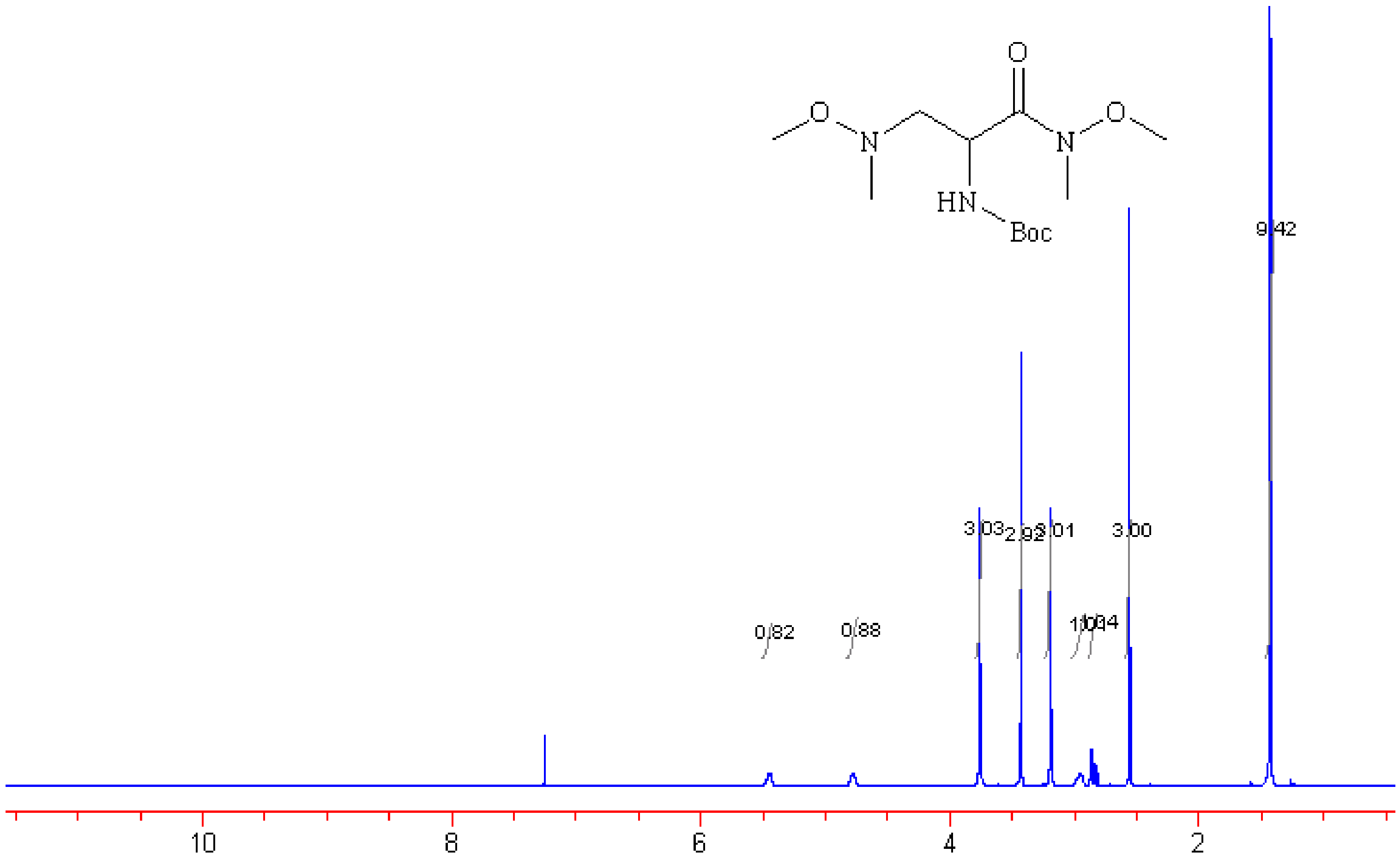

***tert*-Butyl (3,7-dimethyl-4-oxo-2,8-dioxa-3,7-diazanonan-5-yl)carbamate 3e**

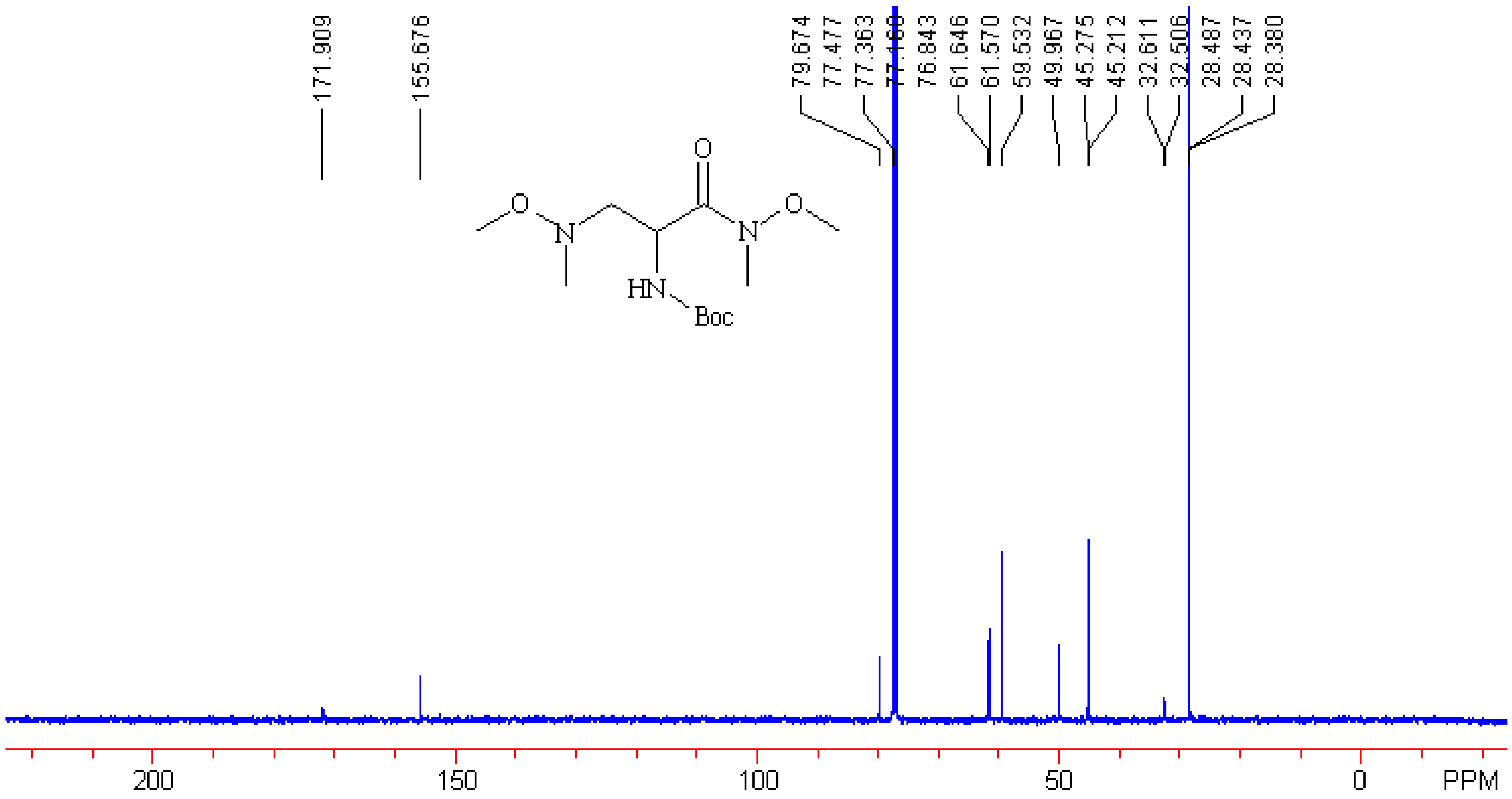

***tert*-Butyl (3,7-dimethyl-4-oxo-2,8-dioxa-3,7-diazanonan-5-yl)carbamate 3e**

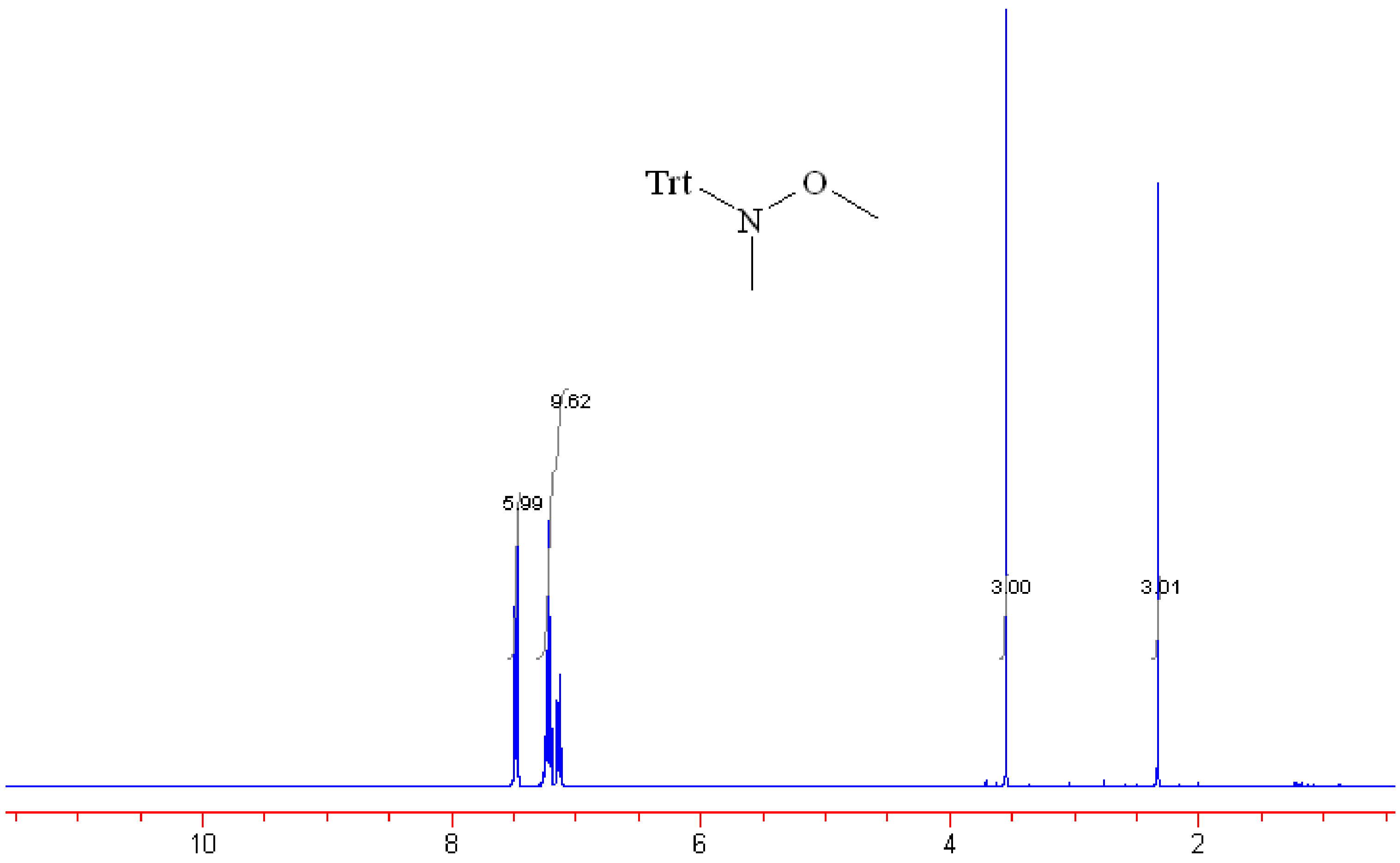

N,O-Dimethyl-N-tritylhydroxylamine 3f

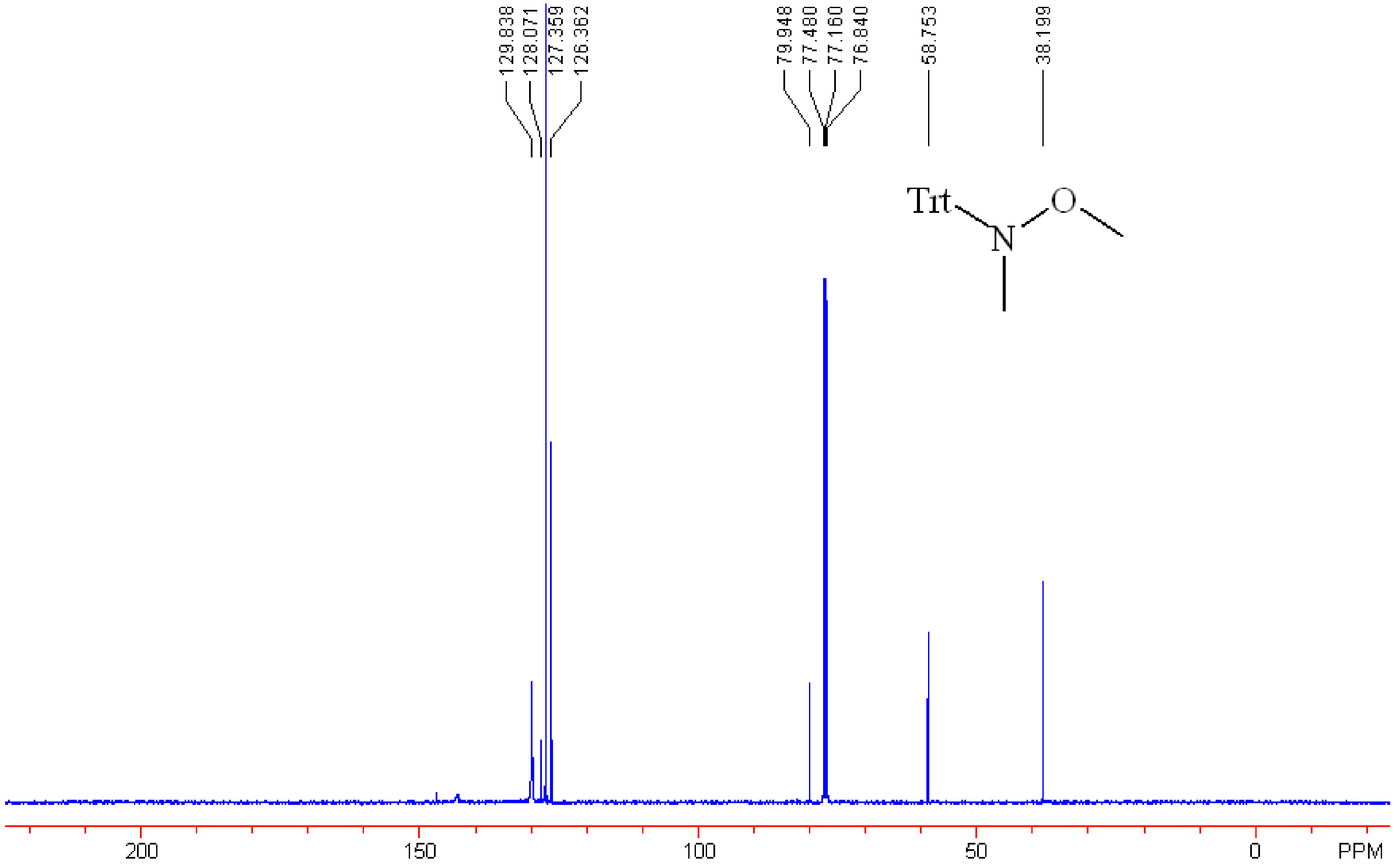

N,O-Dimethyl-N-tritylhydroxylamine 3f

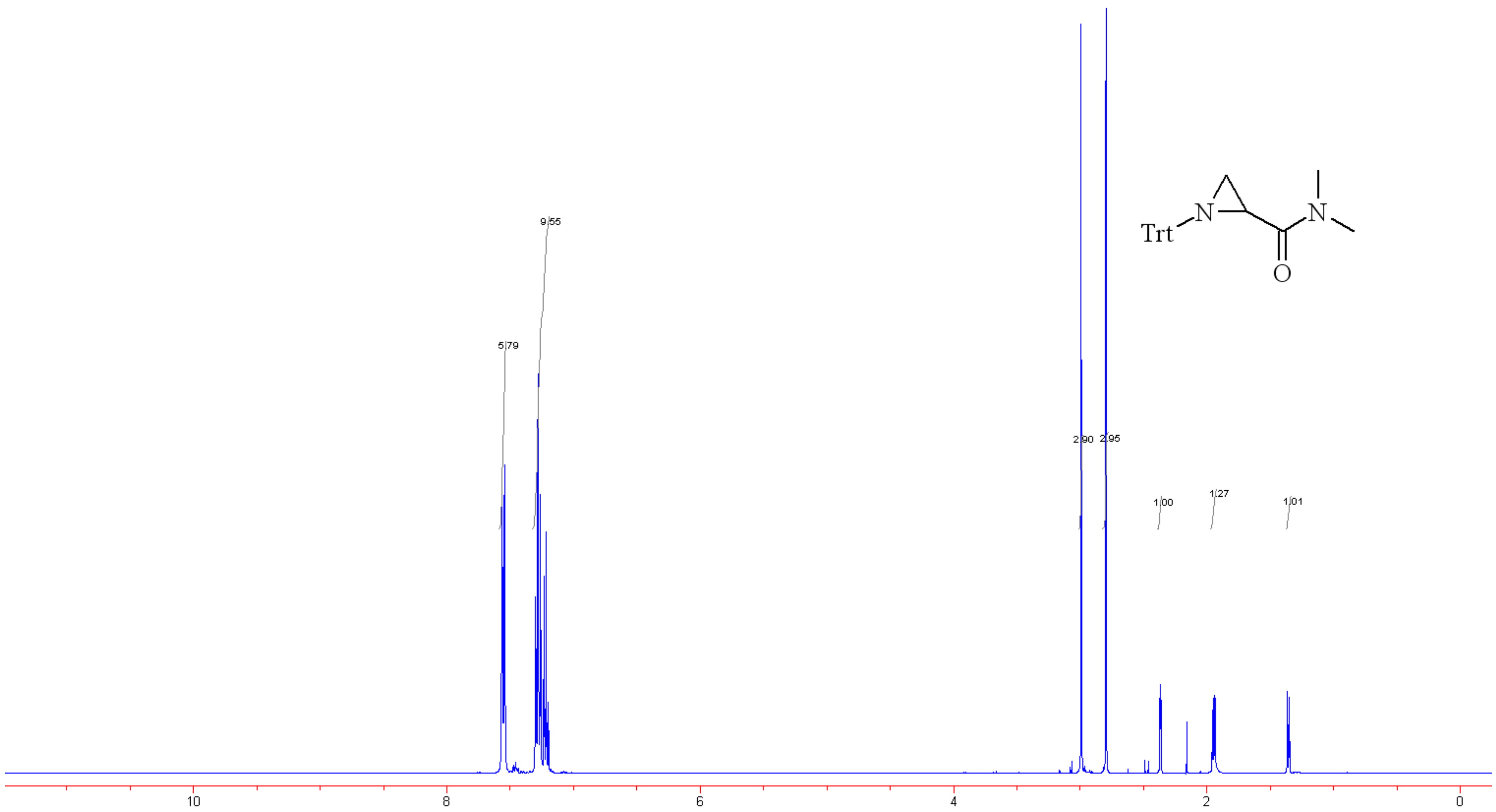

1-Trityl-aziridine-2-carboxylic acid dimethylamide 4a

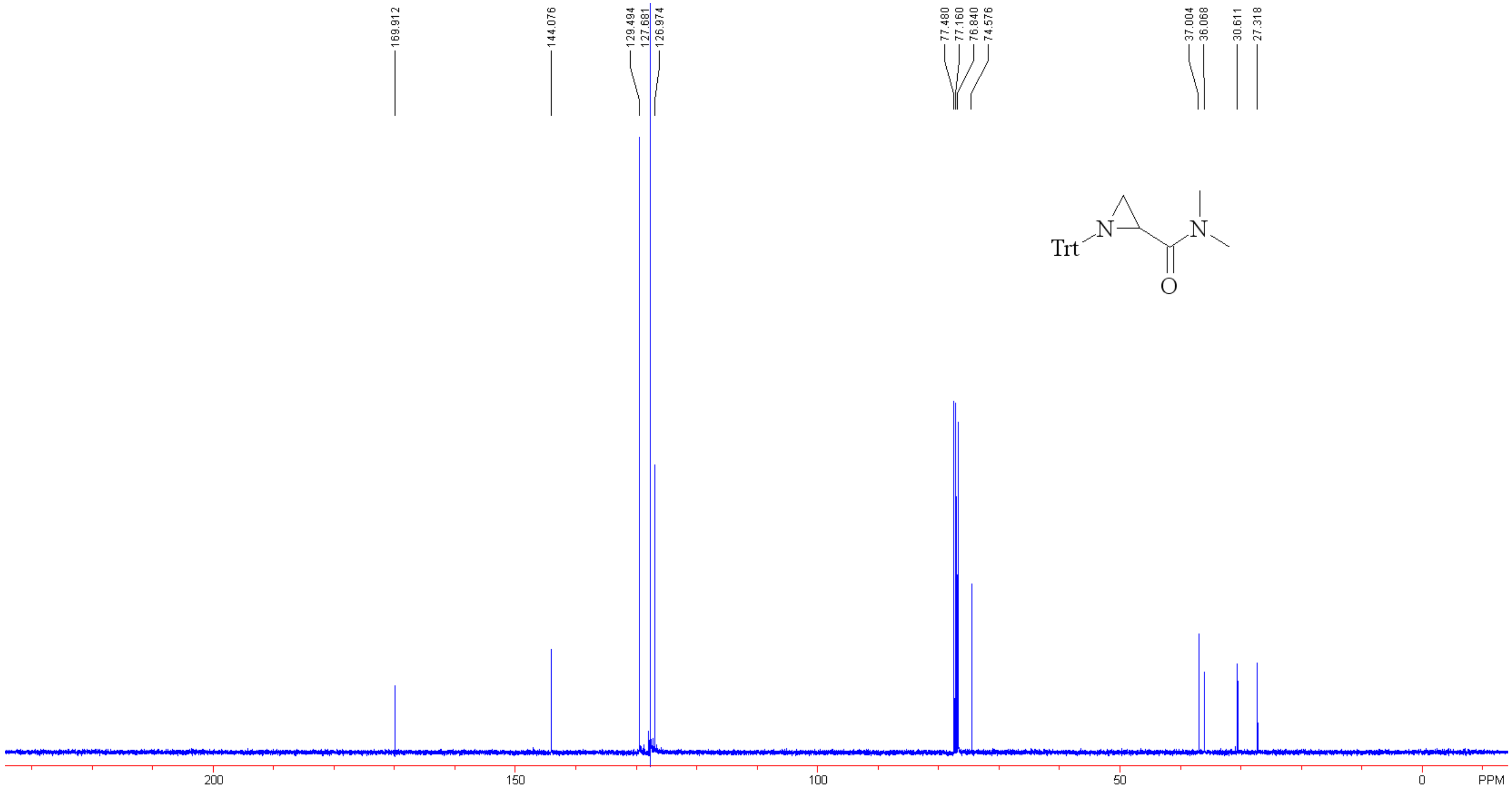

1-Trityl-aziridine-2-carboxylic acid dimethylamide 4a

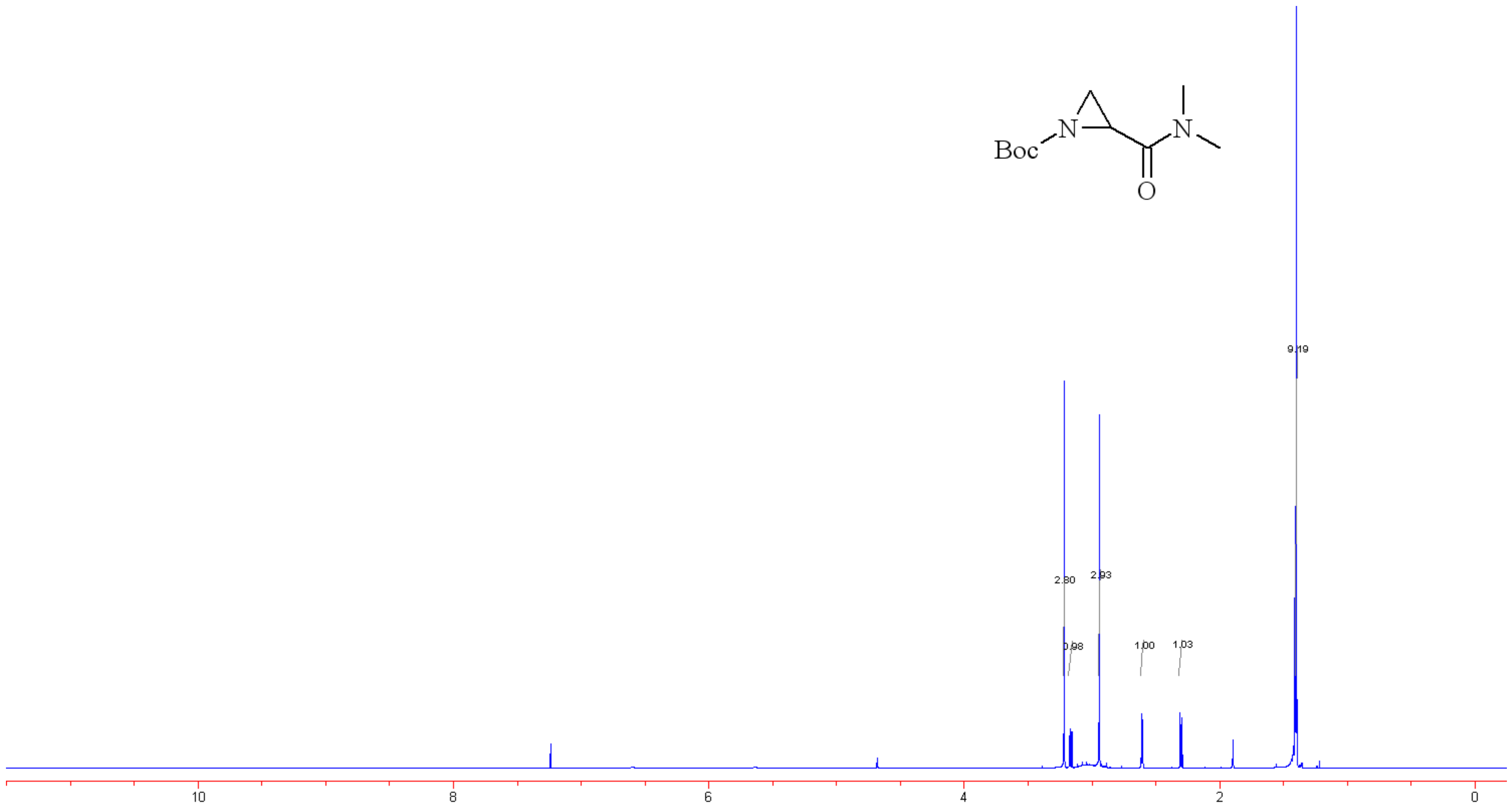

2-Dimethylcarbamoyl-aziridine-1-carboxylic acid *tert*-butyl ester 4b

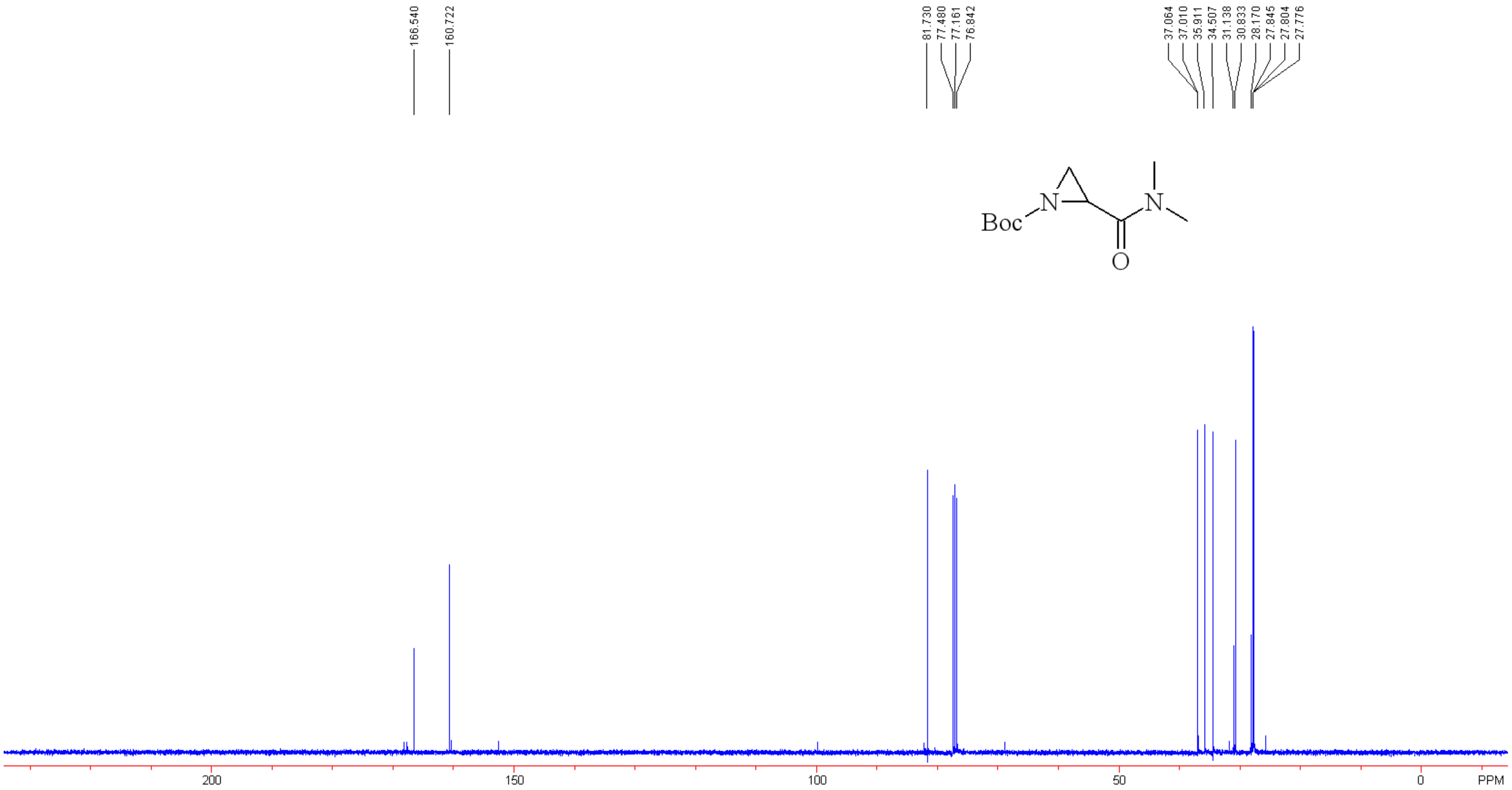

2-Dimethylcarbamoyl-aziridine-1-carboxylic acid *tert*-butyl ester 4b

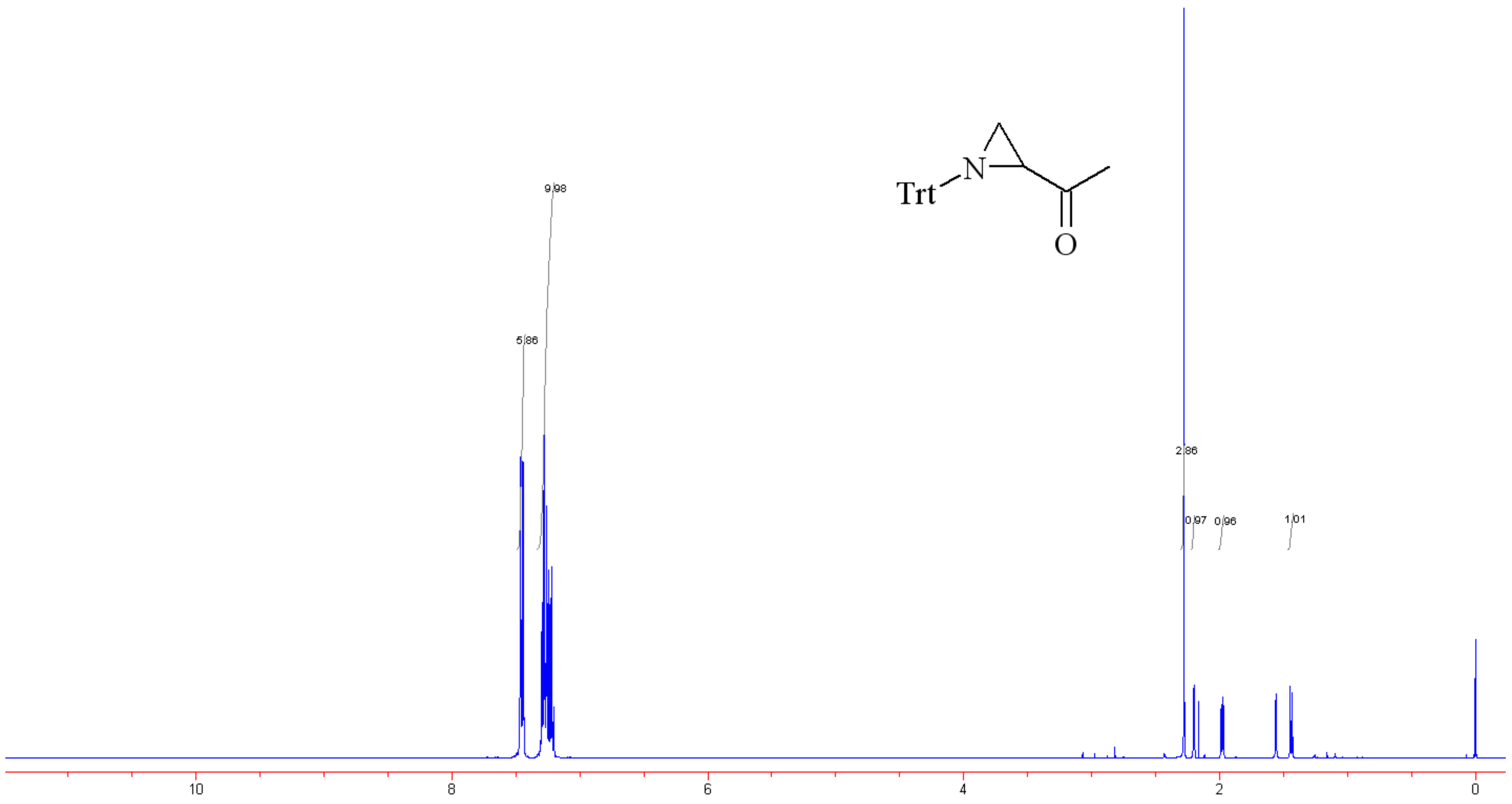

1-(1-Triphenylmethylaziridine-2-yl) ethanone 5a

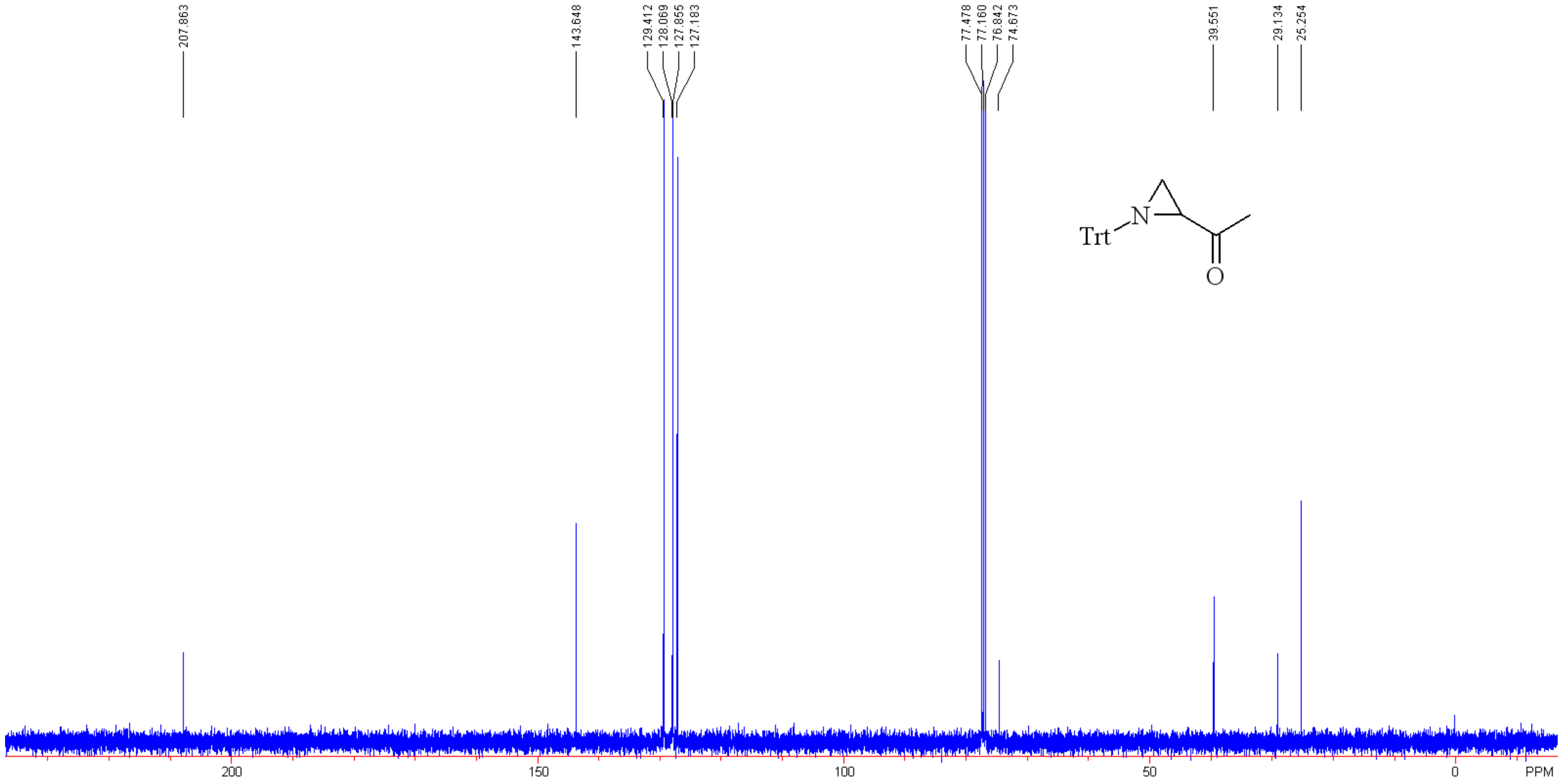

1-(1-Triphenylmethylaziridine-2-yl) ethanone 5a

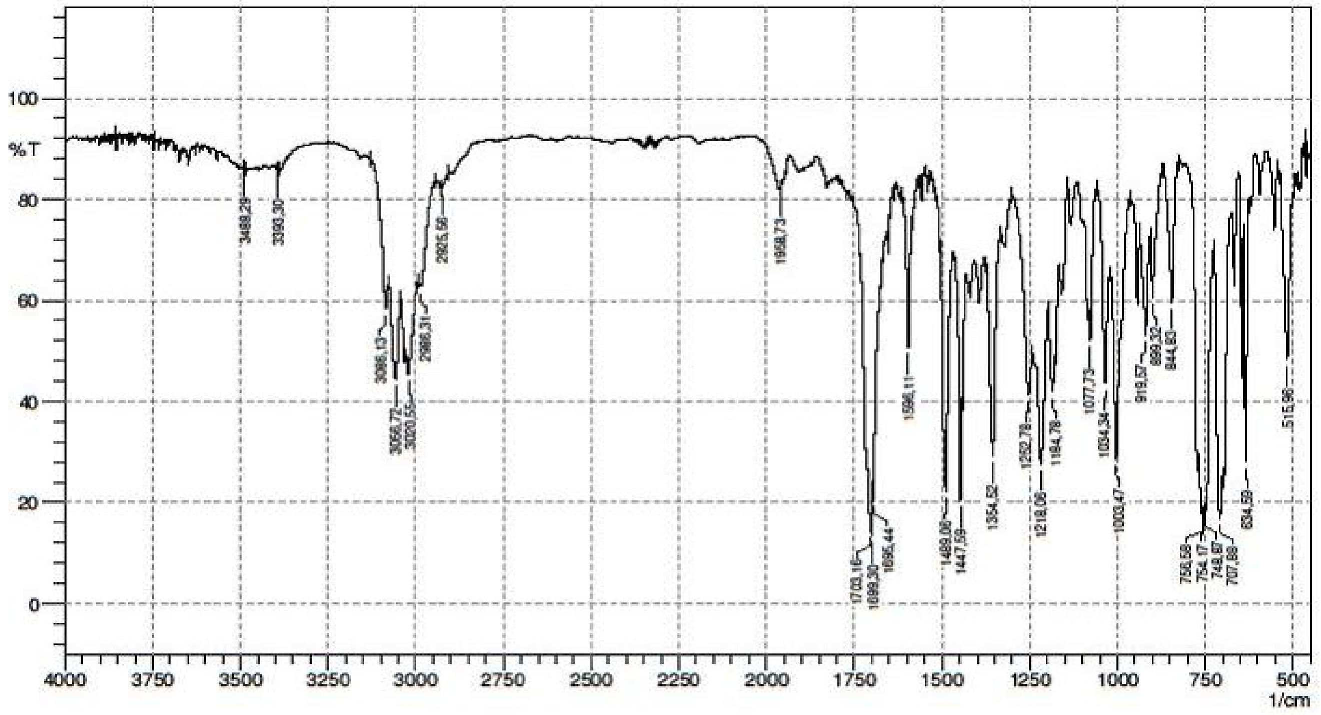

K30

1-(1-Triphenylmethylaziridine-2-yl) ethanone 5a

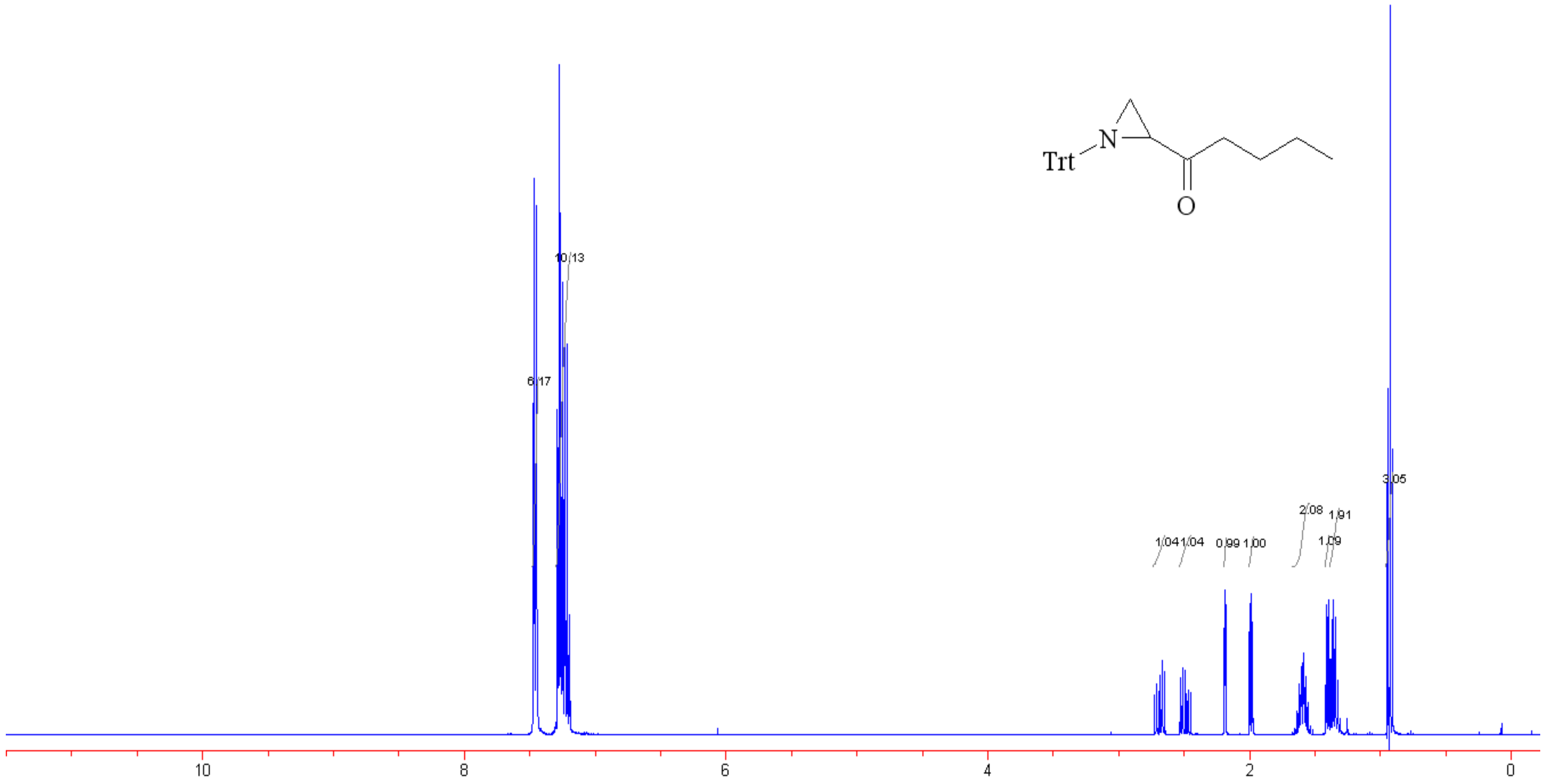

1-(1-Triphenylmethylaziridine-2-yl) pentane-1-one 5b

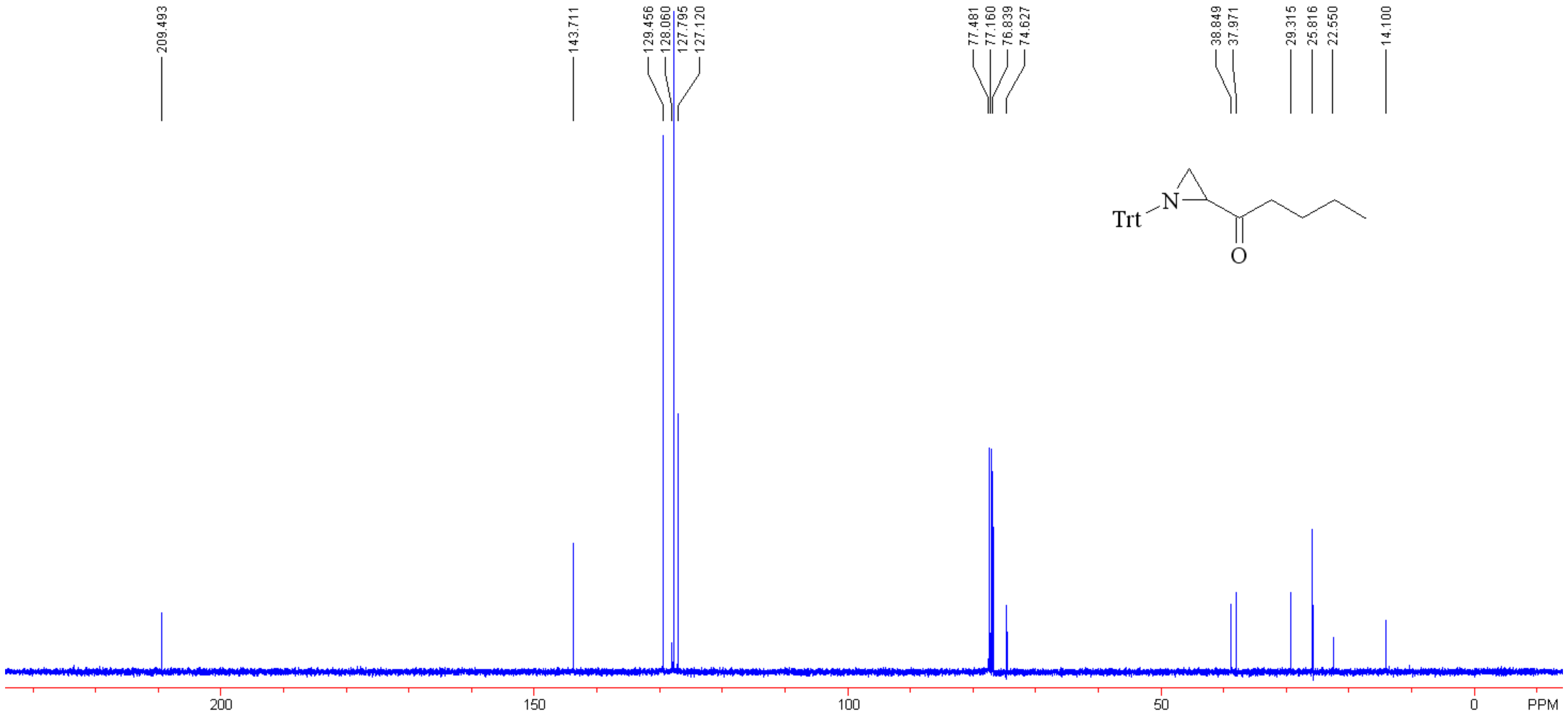

1-(1-Triphenylmethylaziridine-2-yl) pentane-1-one 5b

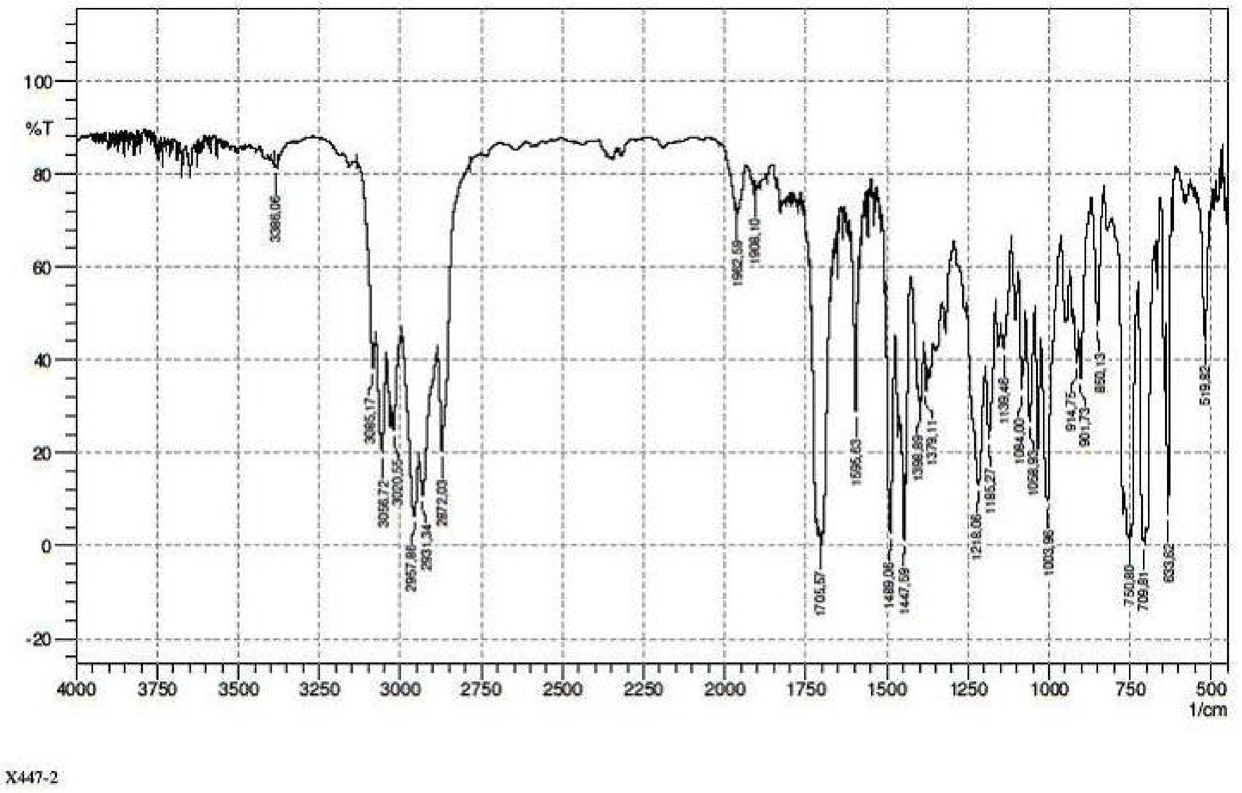

1-(1-Triphenylmethylaziridine-2-yl) pentane-1-one 5b

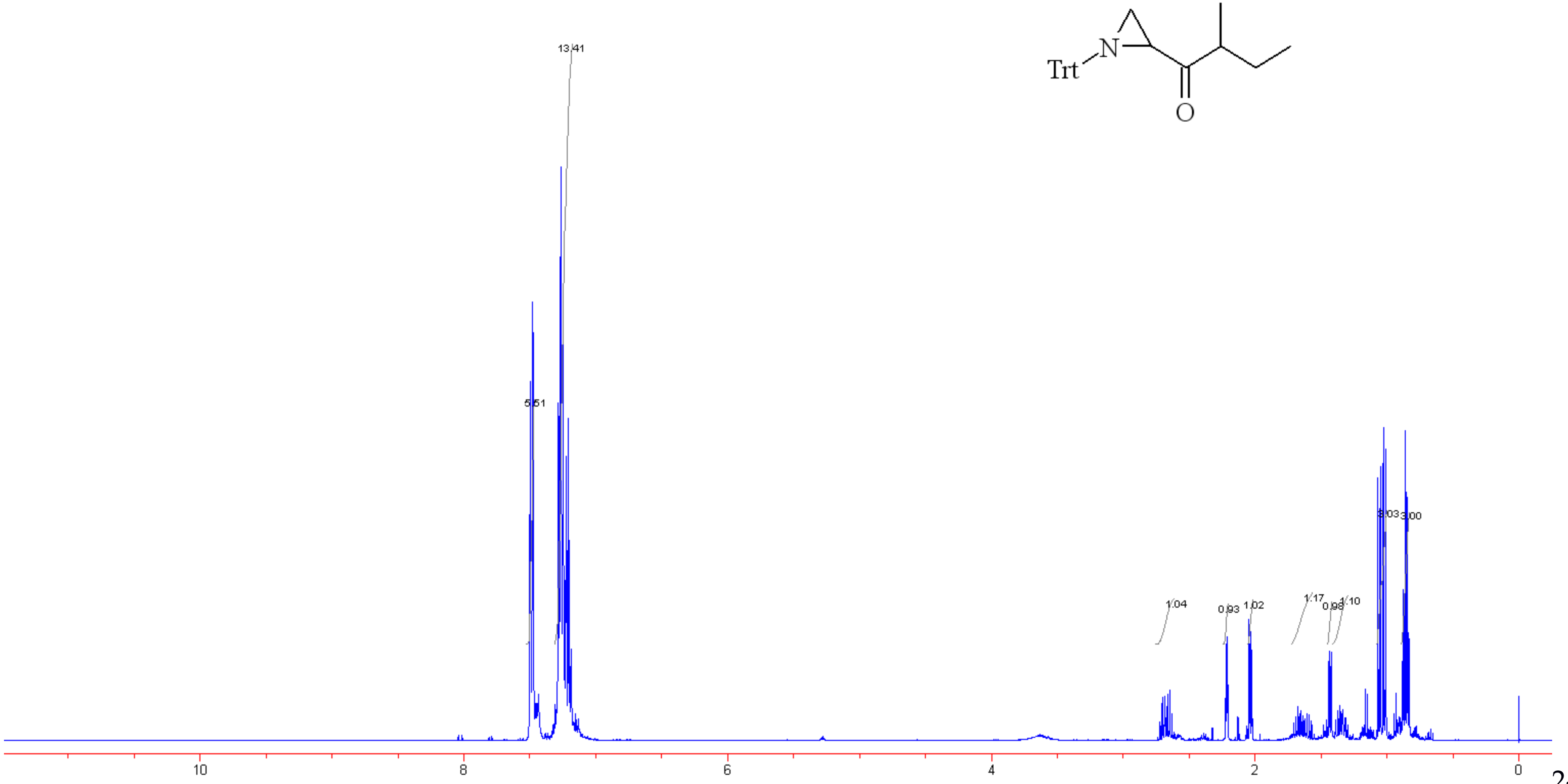

2-
Methyl-1-(1-triphenylmethylaziridine-2-yl)-butane-1-one (mixture of diastereomers) 5c

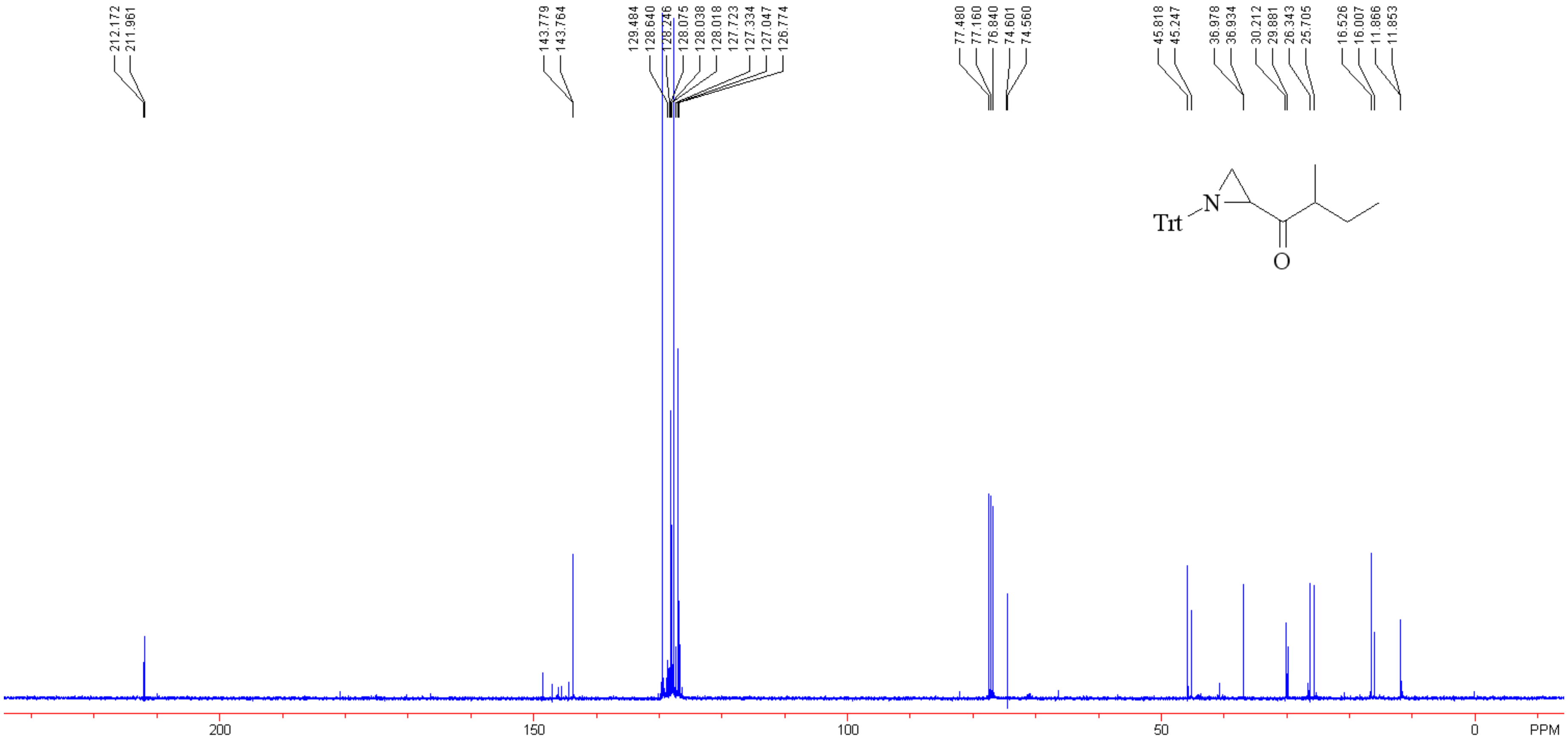

Methyl-1-(1-triphenylmethylaziridine-2-yl)-butane-1-one (mixture of diastereomers) 5c

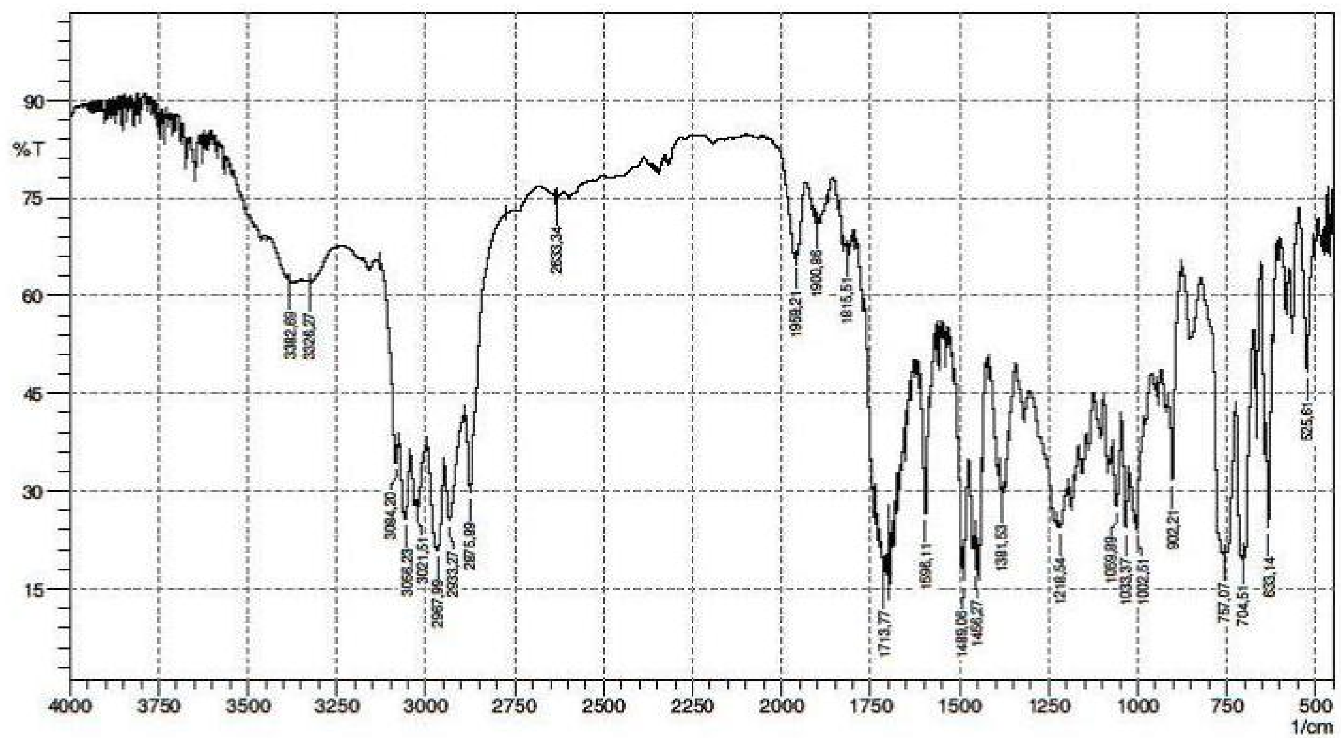

K28H2

Methyl-1-(1-triphenylmethylaziridine-2-yl)-butane-1-one (mixture of diastereomers) 5c

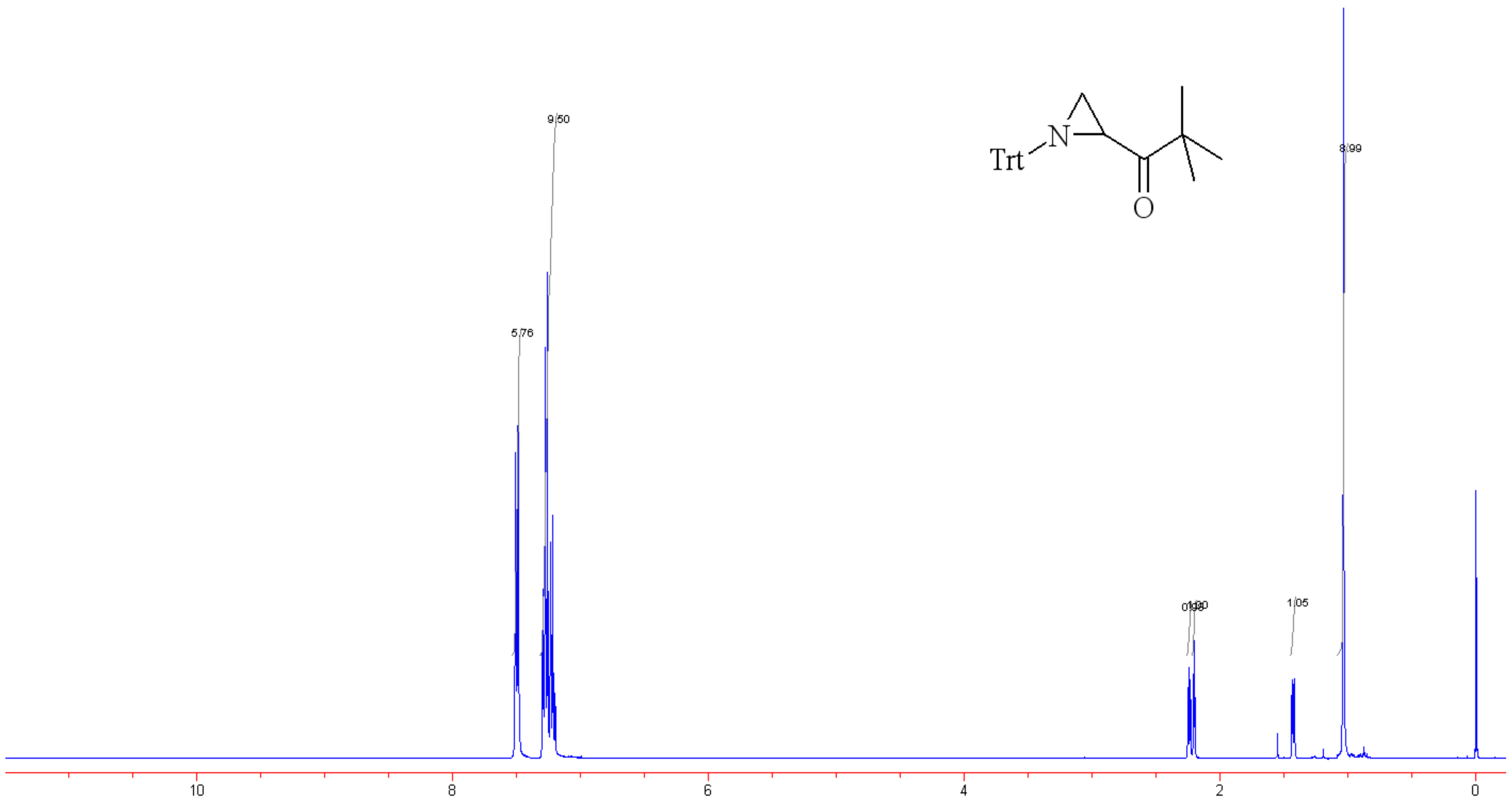

2.2-Dimethyl-1-(1- triphenylmethylaziridine-2-yl)-propane-1-one 5d

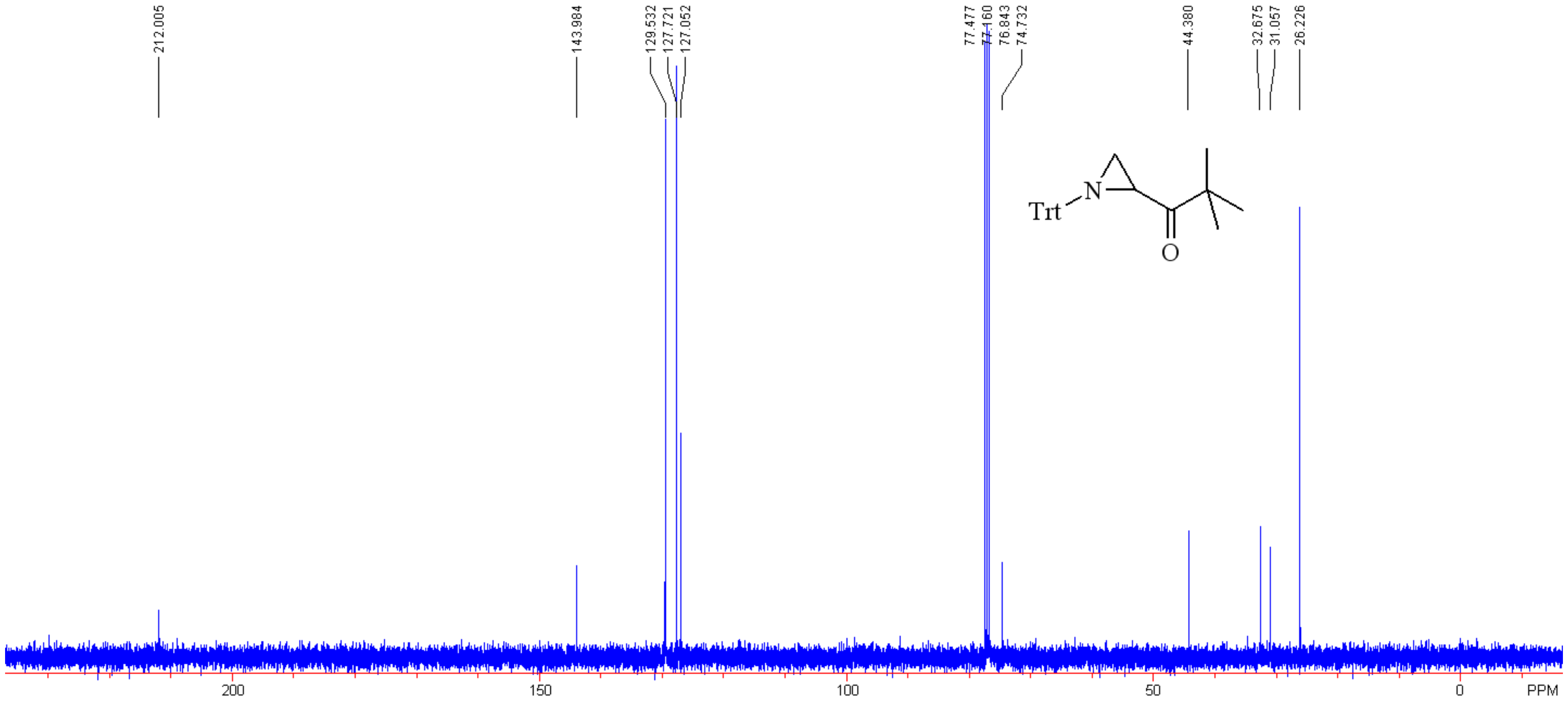

2.2-Dimethyl-1-(1- triphenylmethylaziridine-2-yl)-propane-1-one 5d

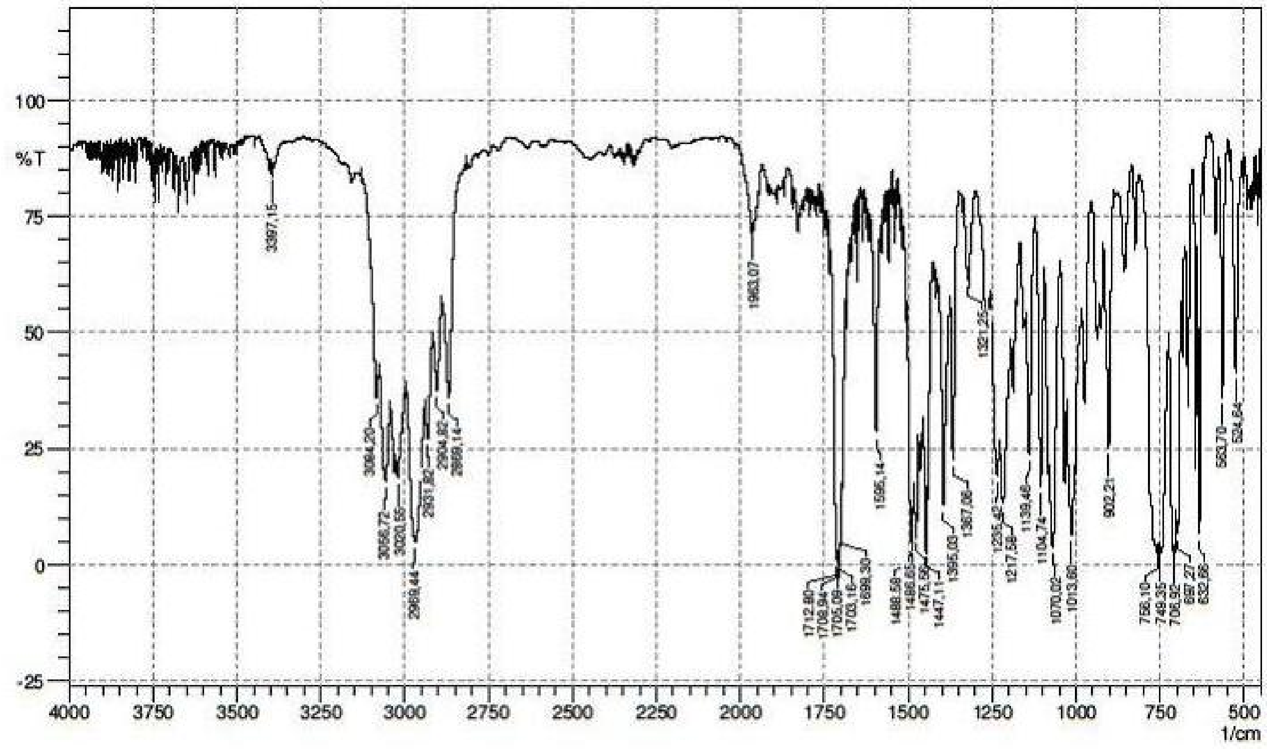

K-38

2.2-Dimethyl-1-(1- triphenylmethylaziridine-2-yl)-propane-1-one 5d

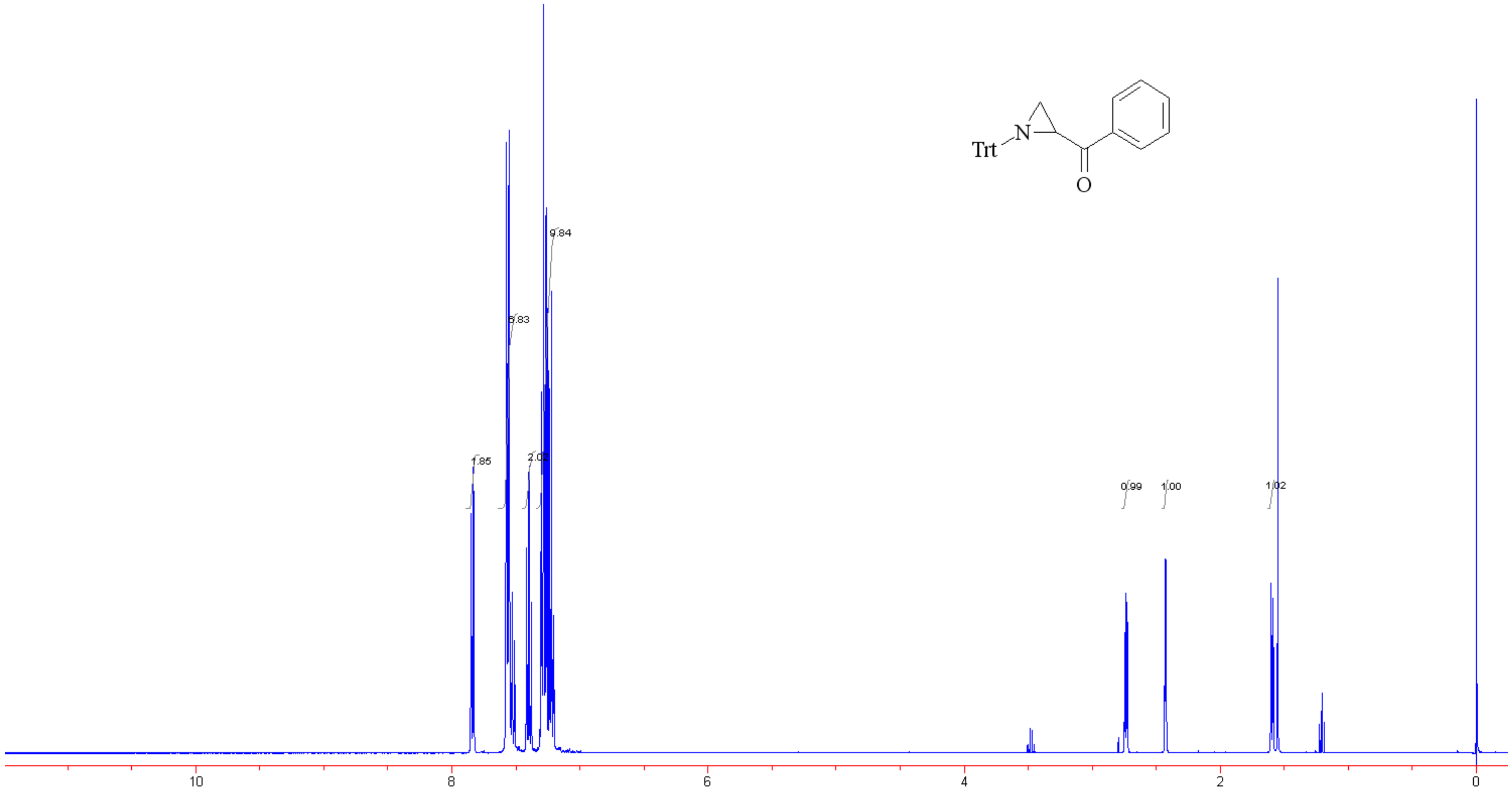

(Triphenylmethylaziridine-2-yl)-phenyl-methanone 5e

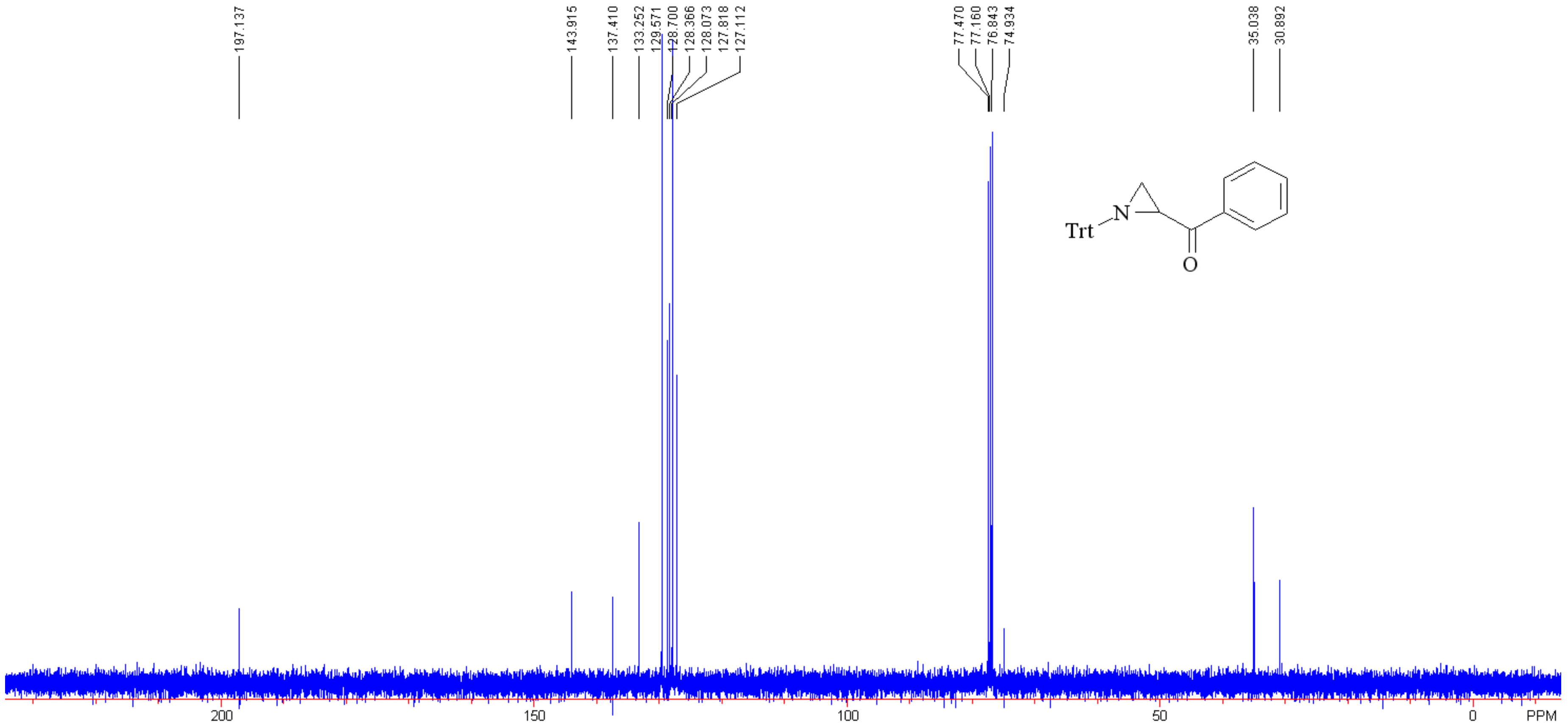

(Triphenylmethylaziridine-2-yl)-phenyl-methanone 5e

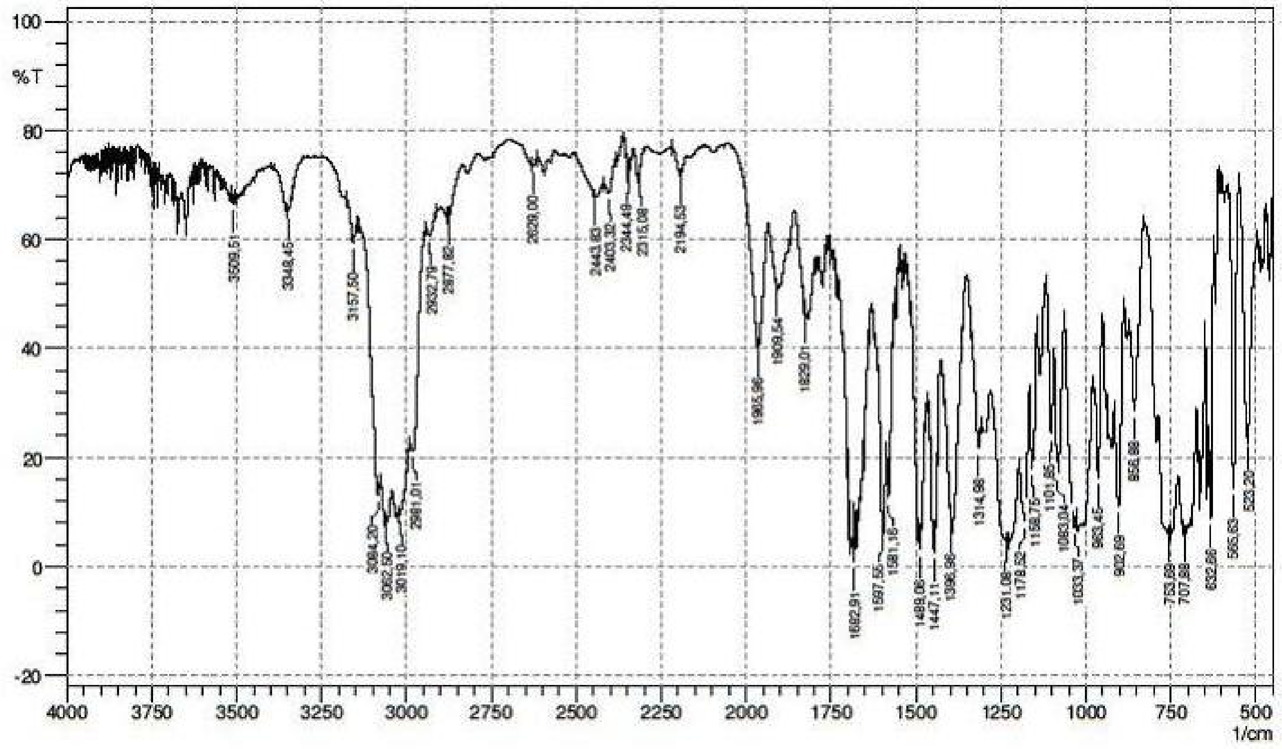

K41

(Triphenylmethylaziridine-2-yl)-phenyl-methanone 5e

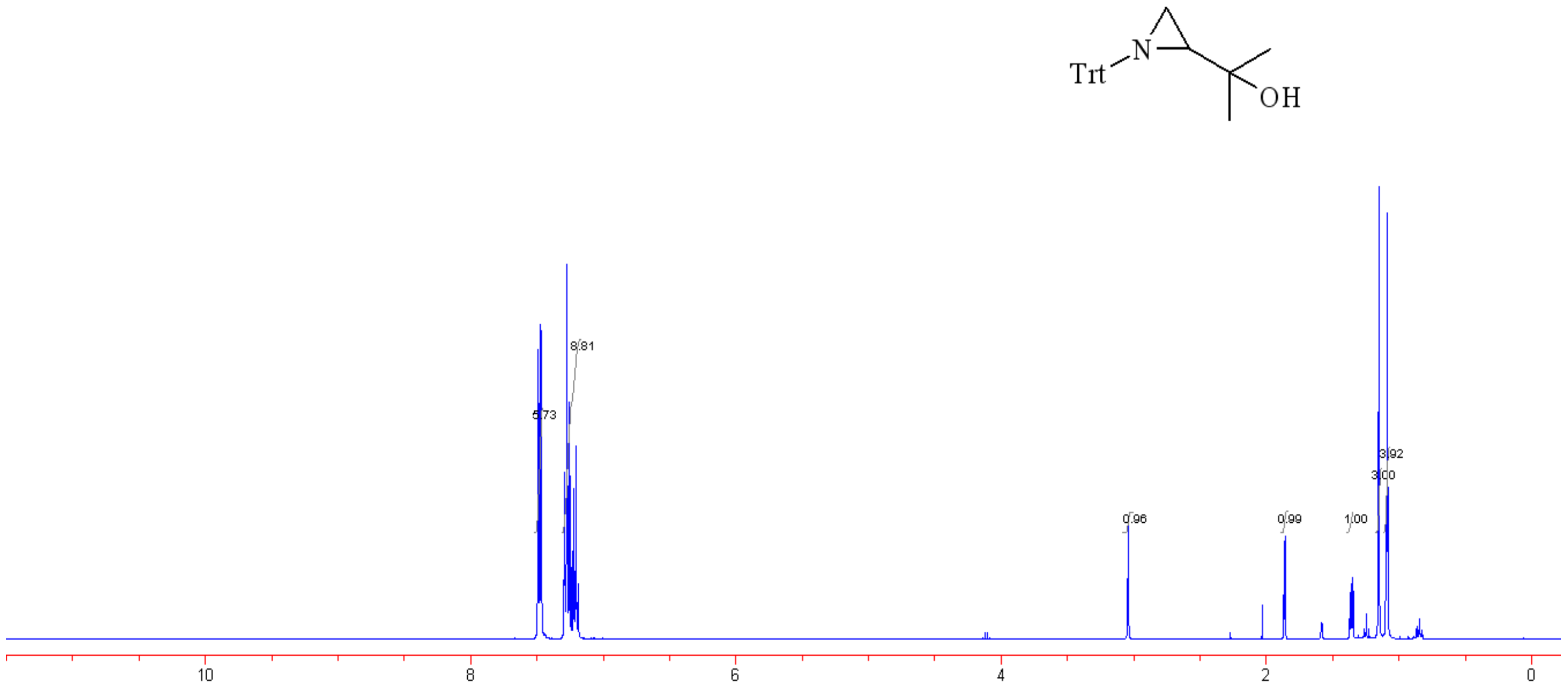

2-(1-Triphenylmethylaziridine-2-yl)-propan-2-ol 6a

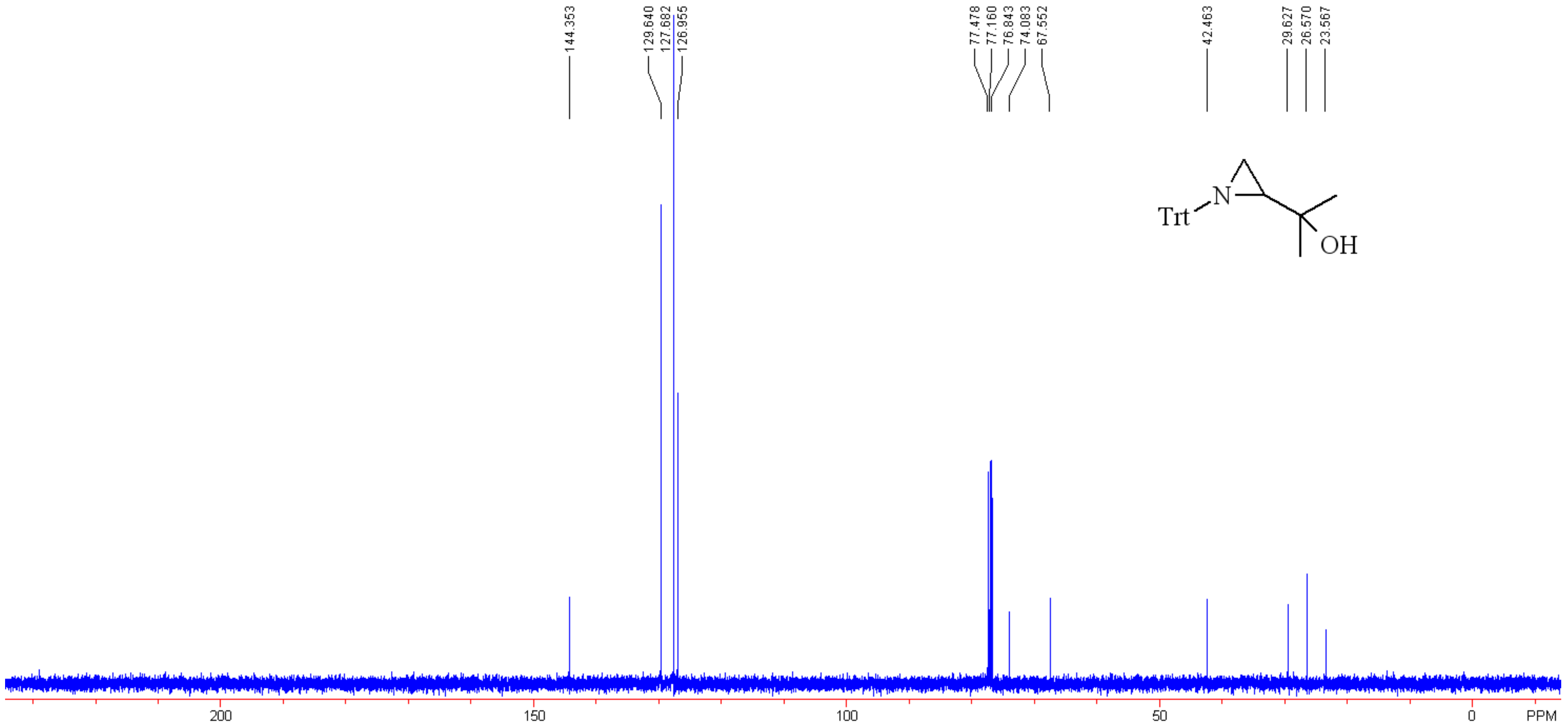

2-(1-Triphenylmethylaziridine-2-yl)-propan-2-ol 6a

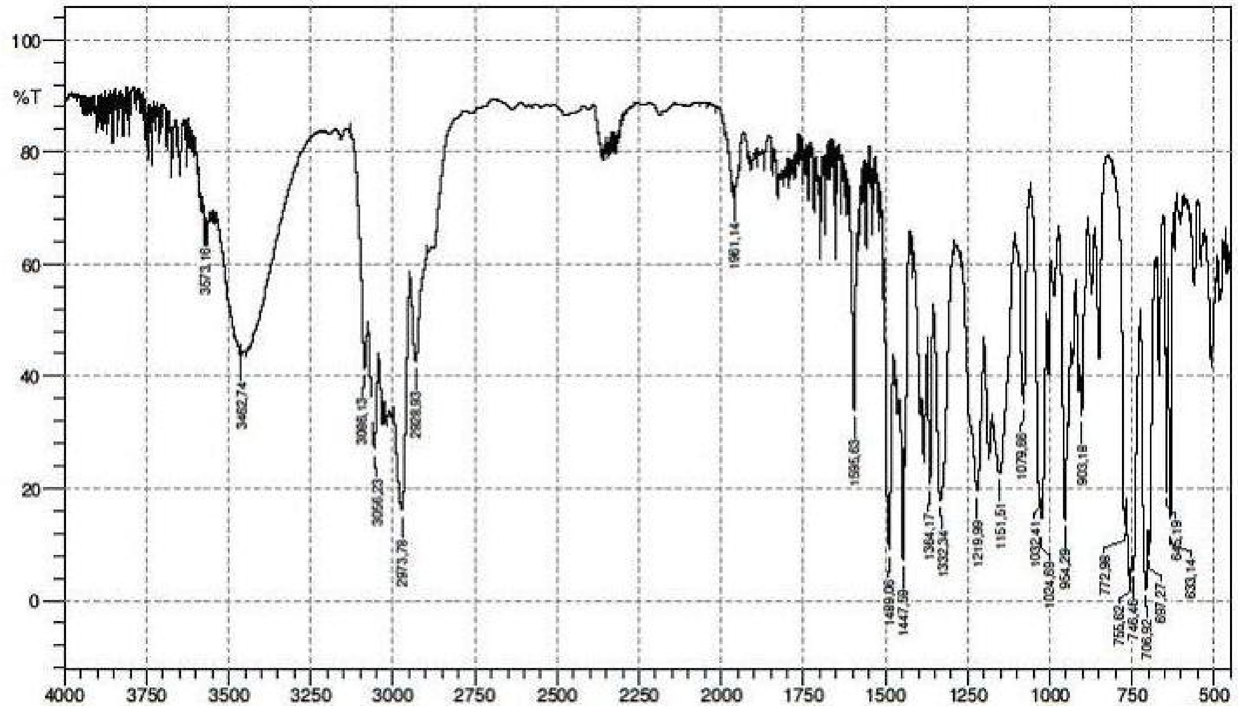

X1009F2

2-(1-Triphenylmethylaziridine-2-yl)-propan-2-ol 6a

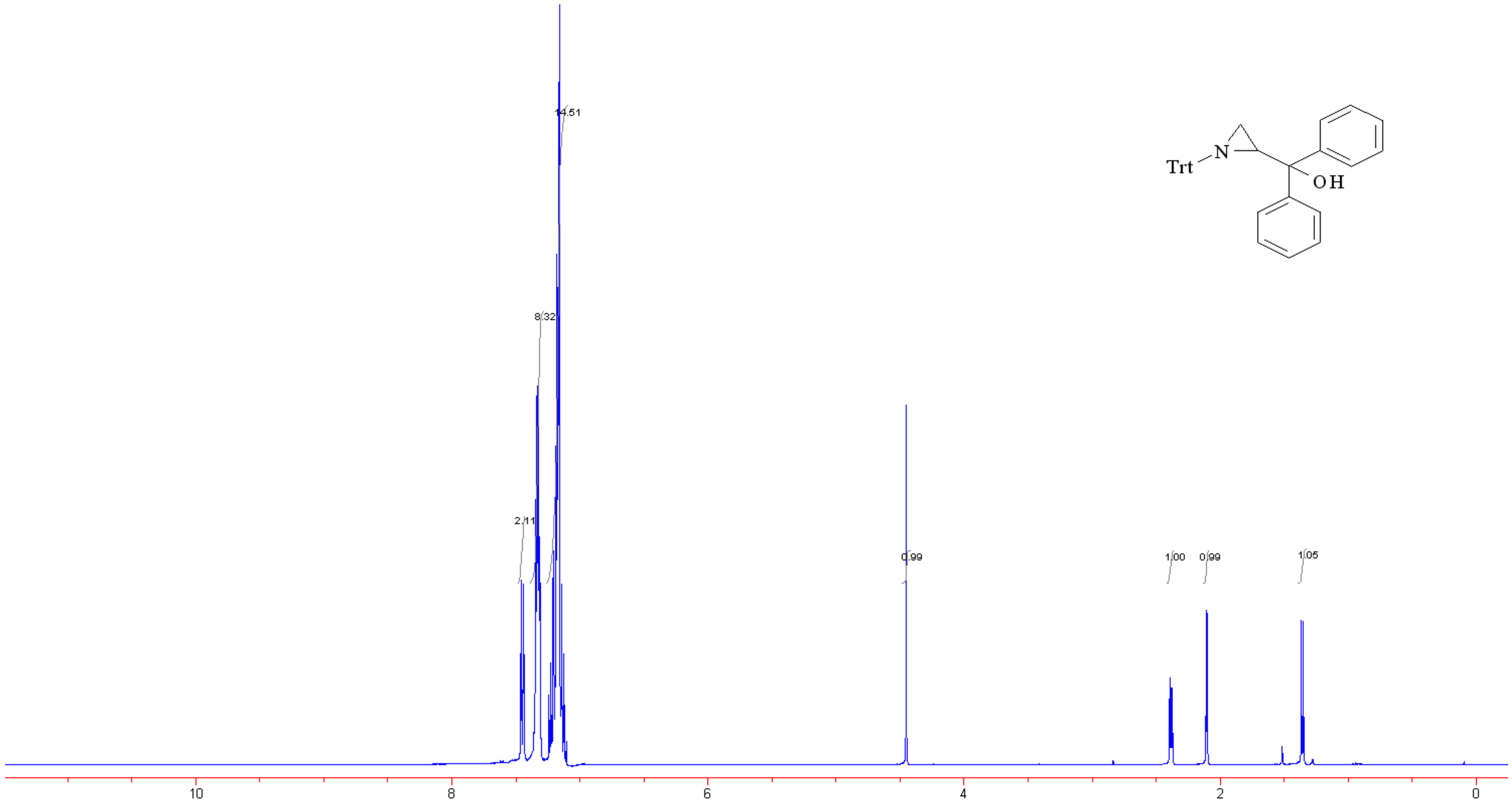

Diphenyl-(1-triphenylmethylaziridine-2-yl)-methanol 6b

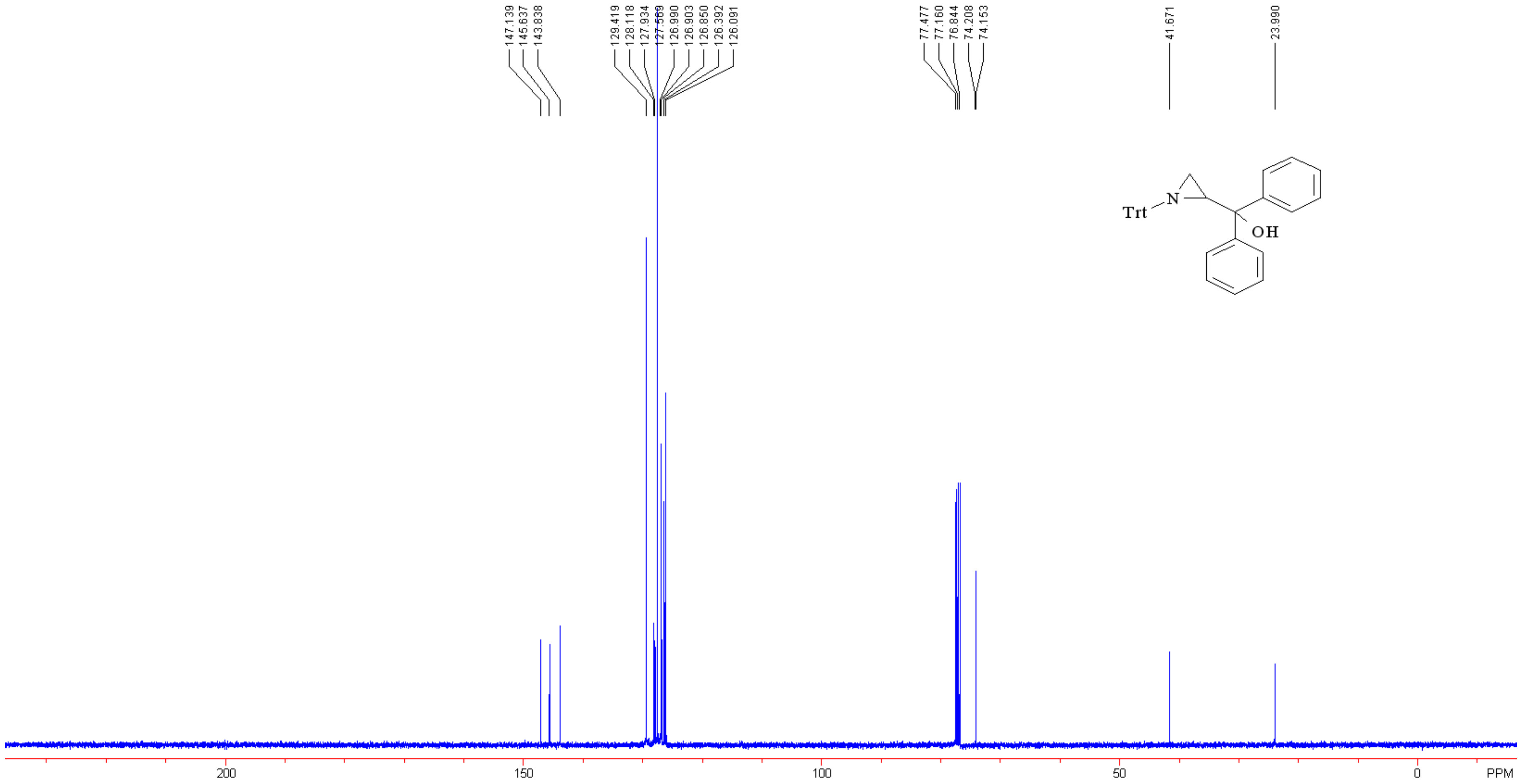

Diphenyl-(1-triphenylmethylaziridine-2-yl)-methanol 6b

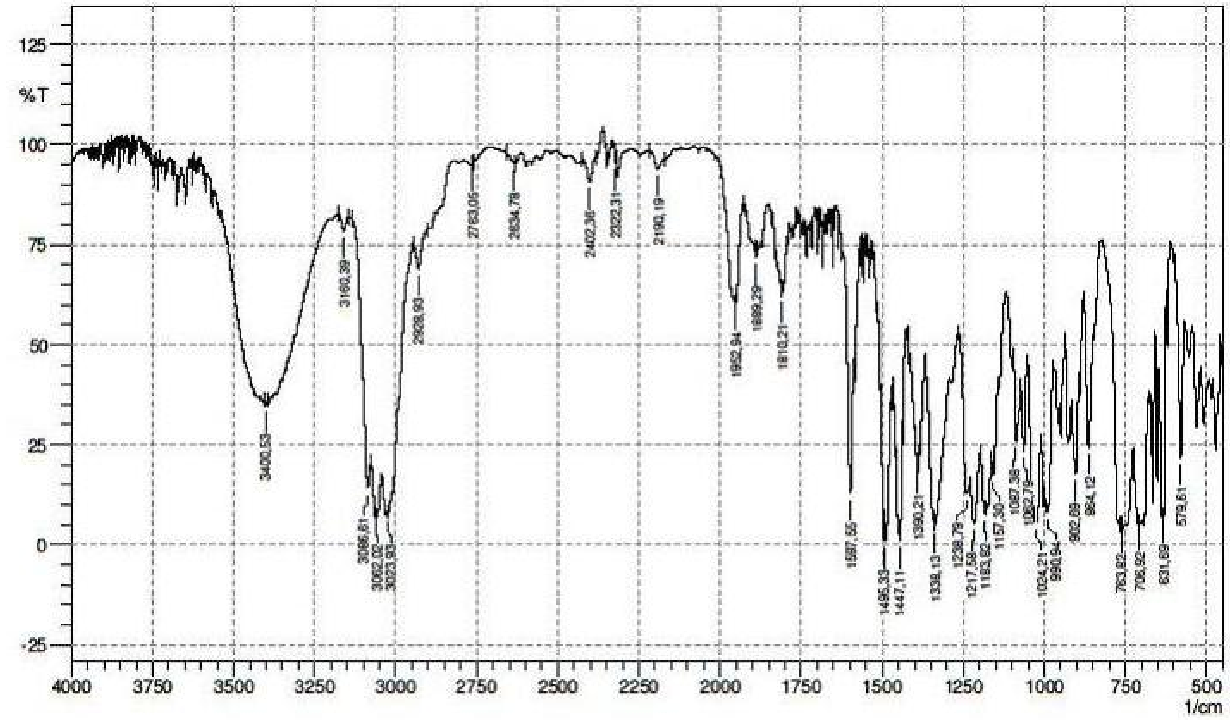

K46

Diphenyl-(1-triphenylmethylaziridine-2-yl)-methanol 6b

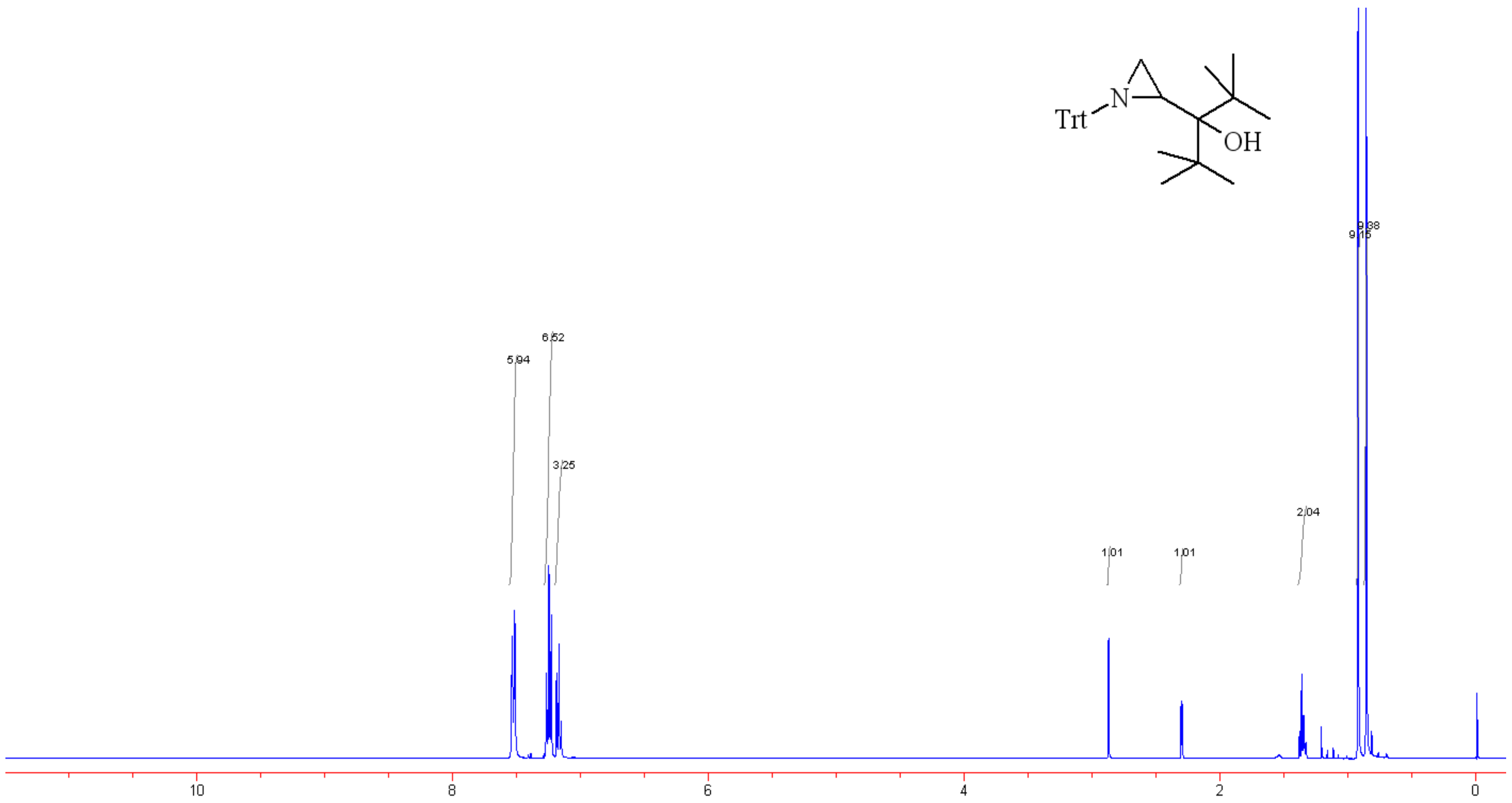

2,2,4,4-Tetramethyl-(1-triphenylmethylaziridine-2-yl)-pentane-3-ol 6c

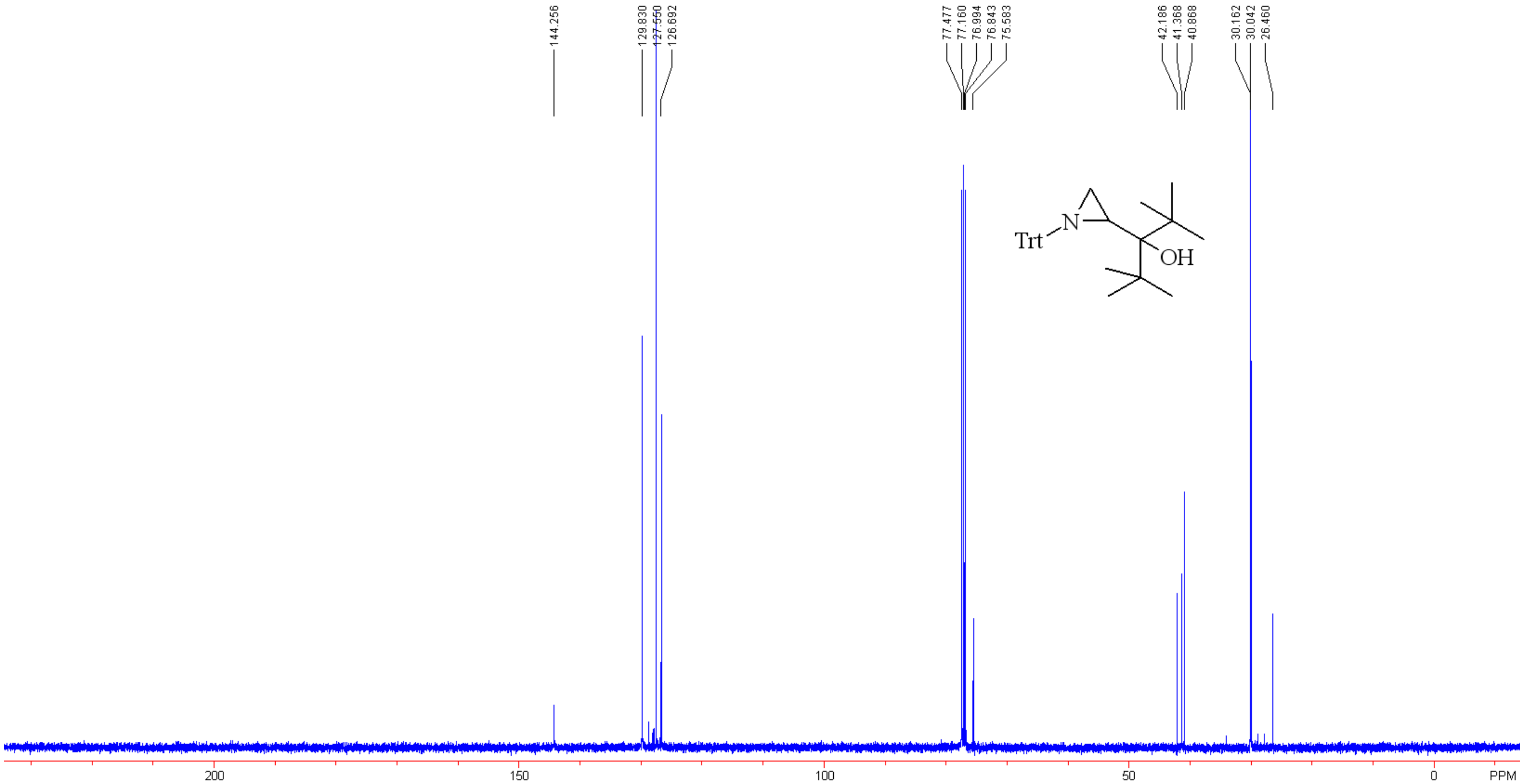

2,2,4,4-Tetramethyl-(1-triphenylmethylaziridine-2-yl)-pentane-3-ol 6c

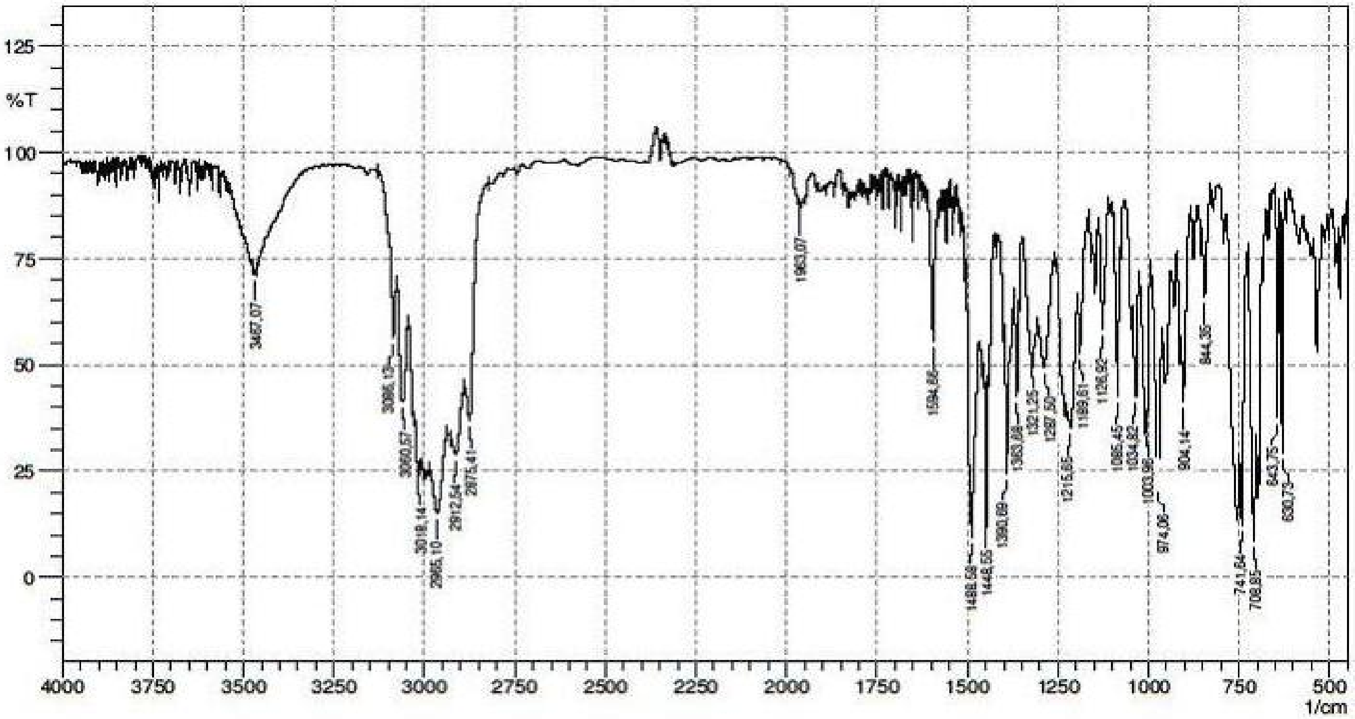

K34

2,2,4,4-Tetramethyl-(1-triphenylmethylaziridine-2-yl)-pentane-3-ol 6c

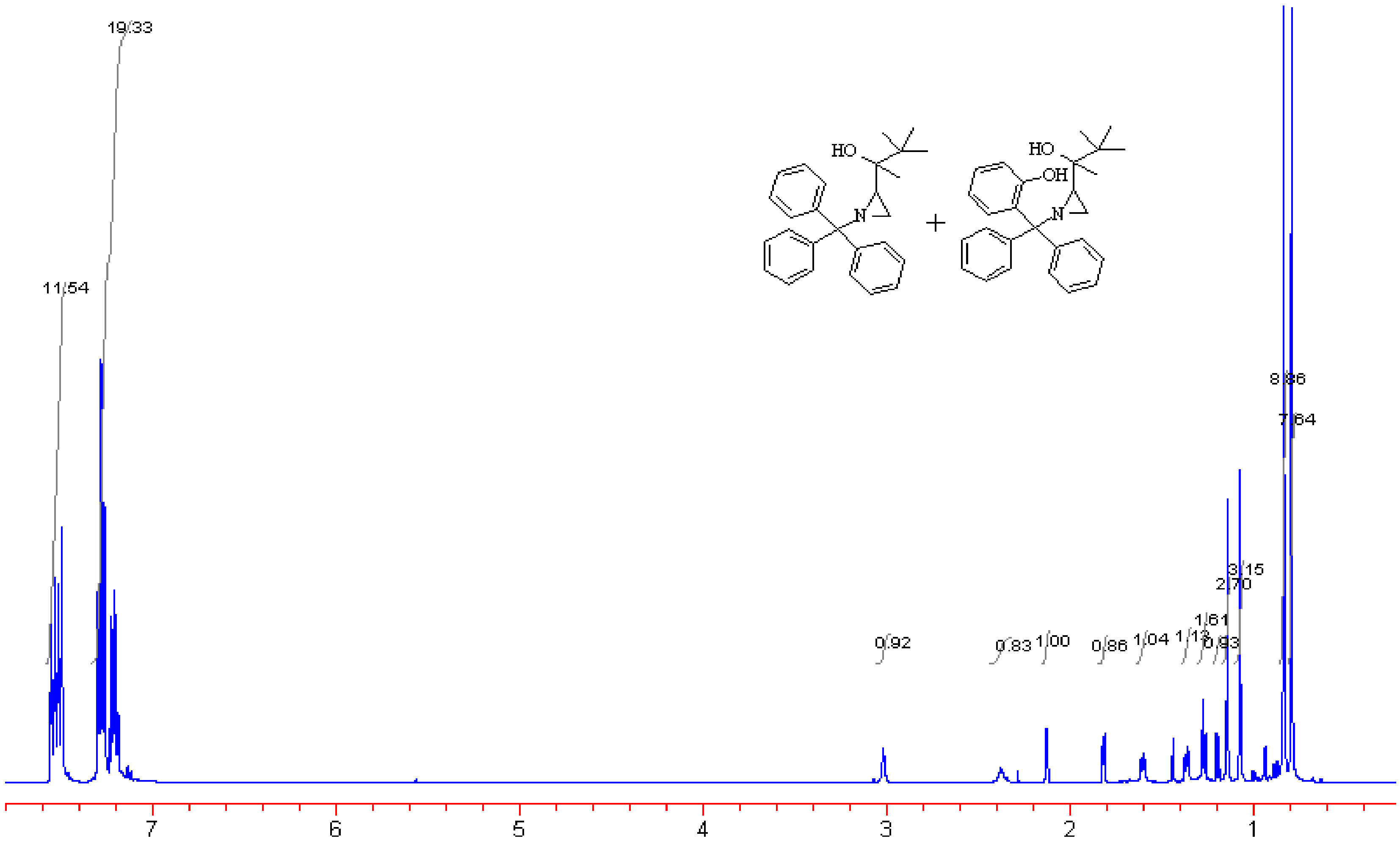

Mixture of 3,3-Dimethyl-2-(1-trityl-aziridin-2-yl)-butan-2-ol 6d (mixture of diastereomers) and phenol 6d1

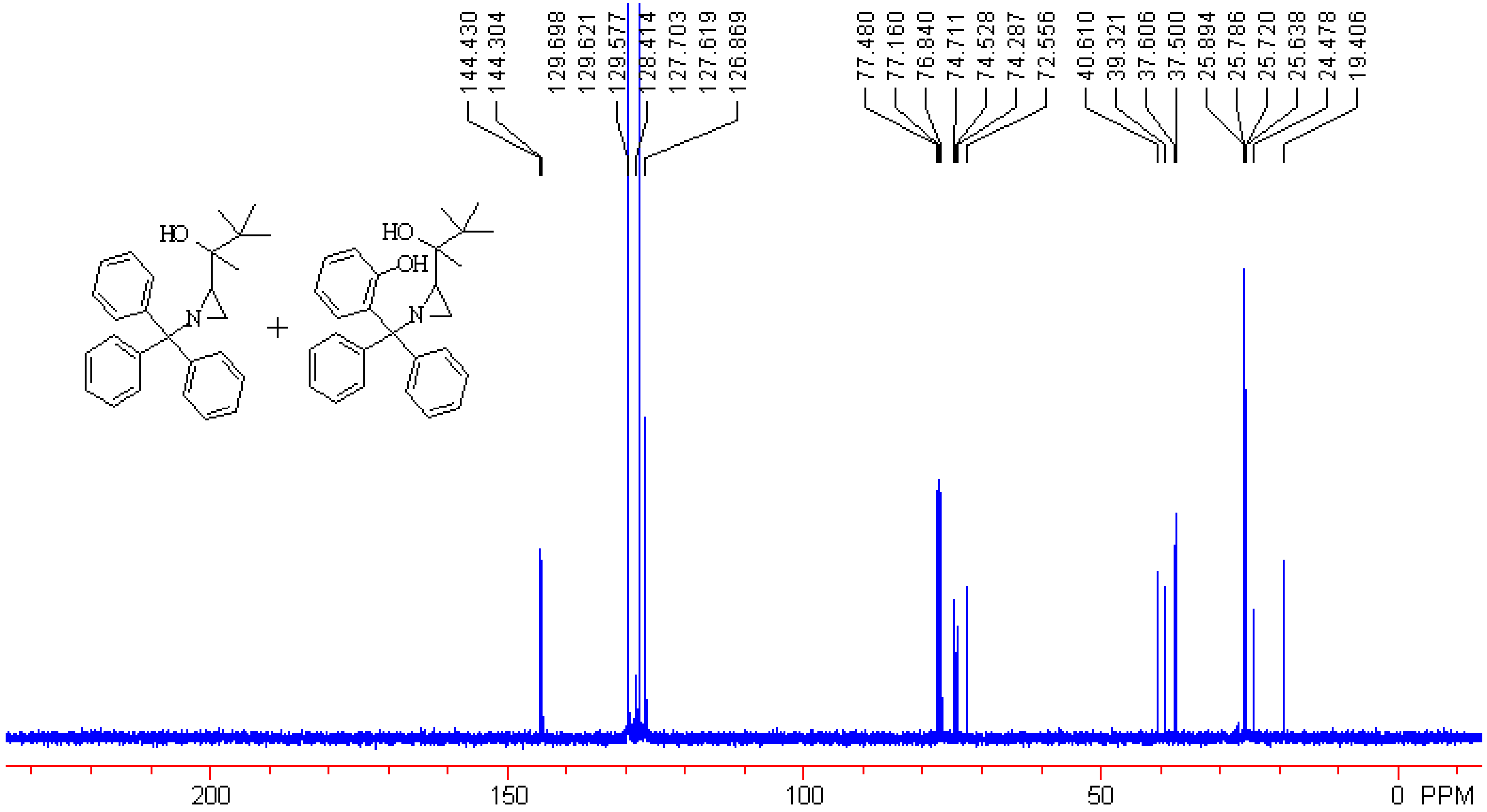

Mixture of 3,3-Dimethyl-2-(1-trityl-aziridin-2-yl)-butan-2-ol 6d (mixture of diastereomers) and phenol 6d1

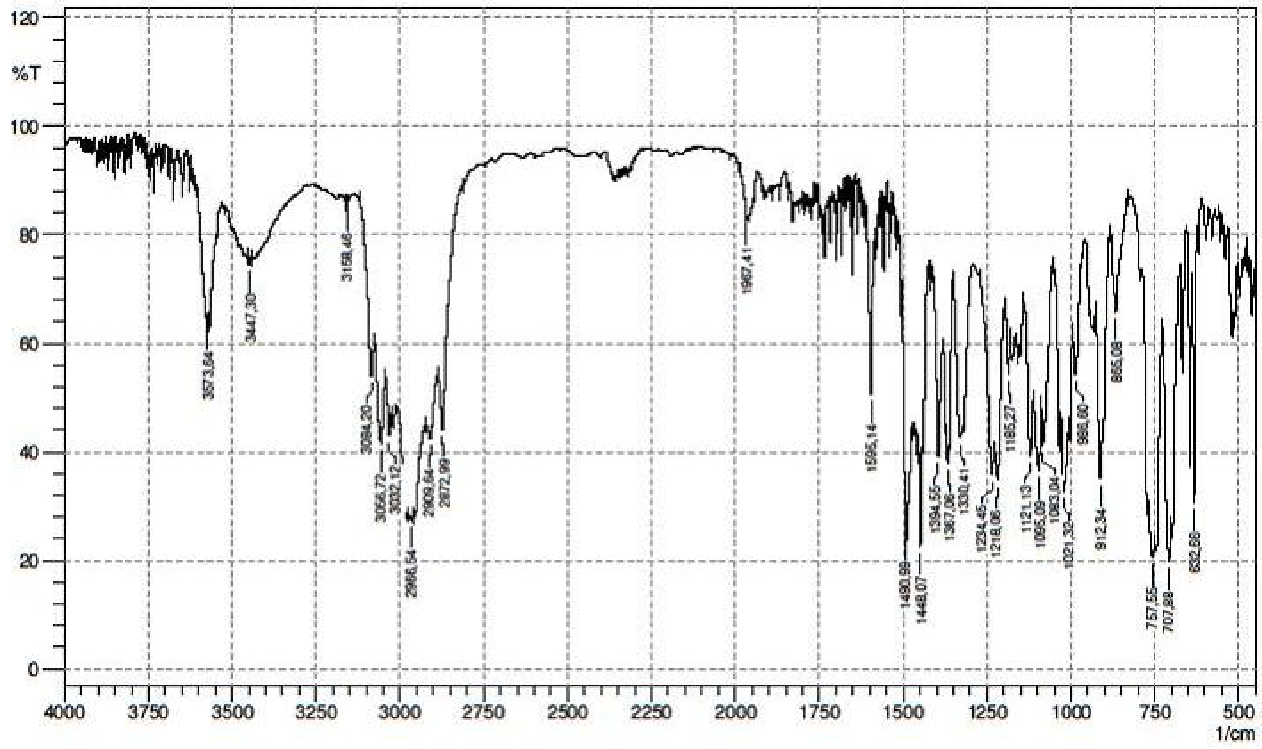

K-37

Mixture of 3,3-Dimethyl-2-(1-trityl-aziridin-2-yl)-butan-2-ol 6d (mixture of diastereomers) and phenol 6d1

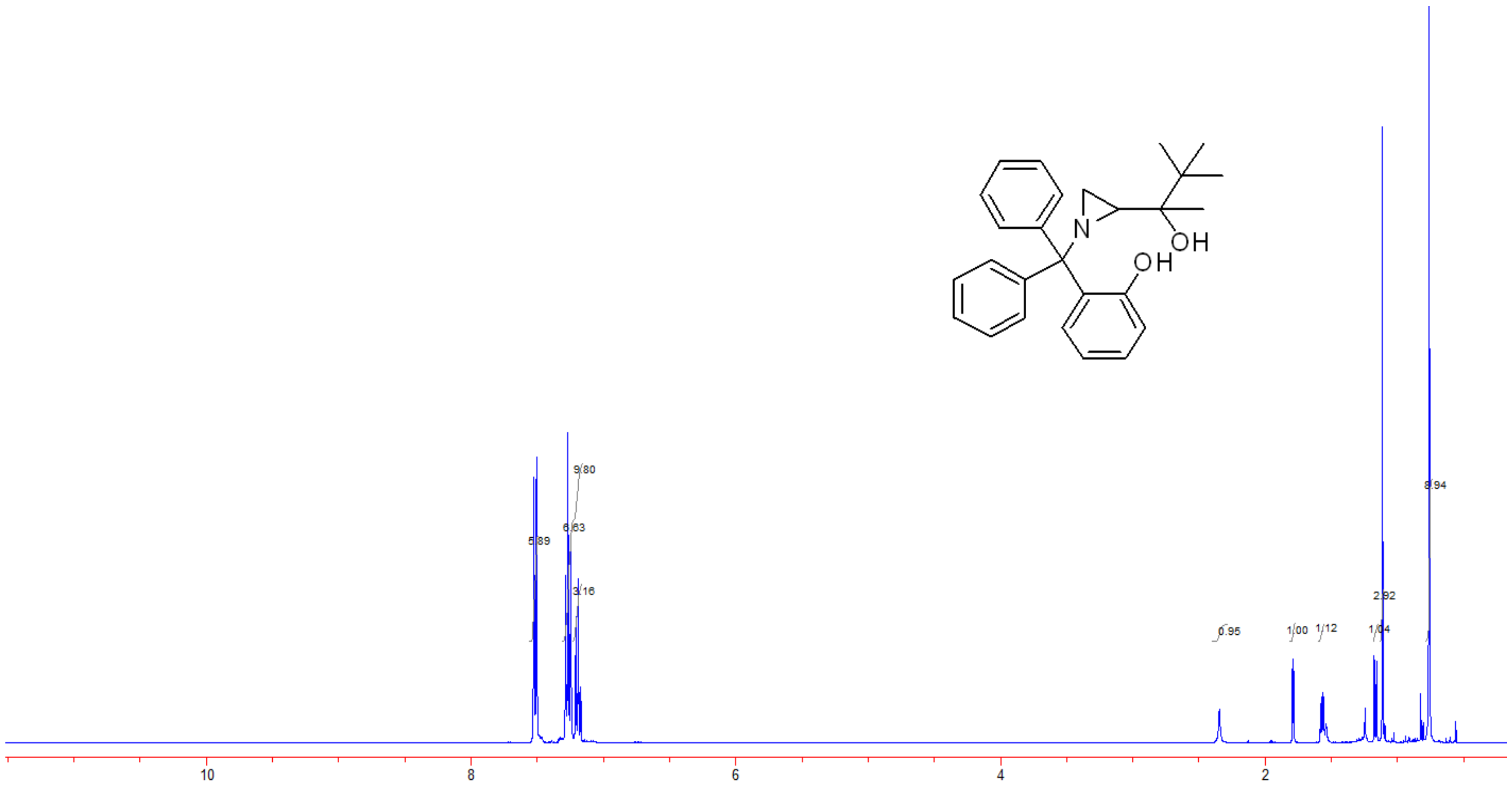

2-{[2-(1-Hydroxy-1,2,2-trimethyl-propyl)-aziridin-1-yl]diphenyl-methyl}phenol (mixture of diastereomers) 6d1

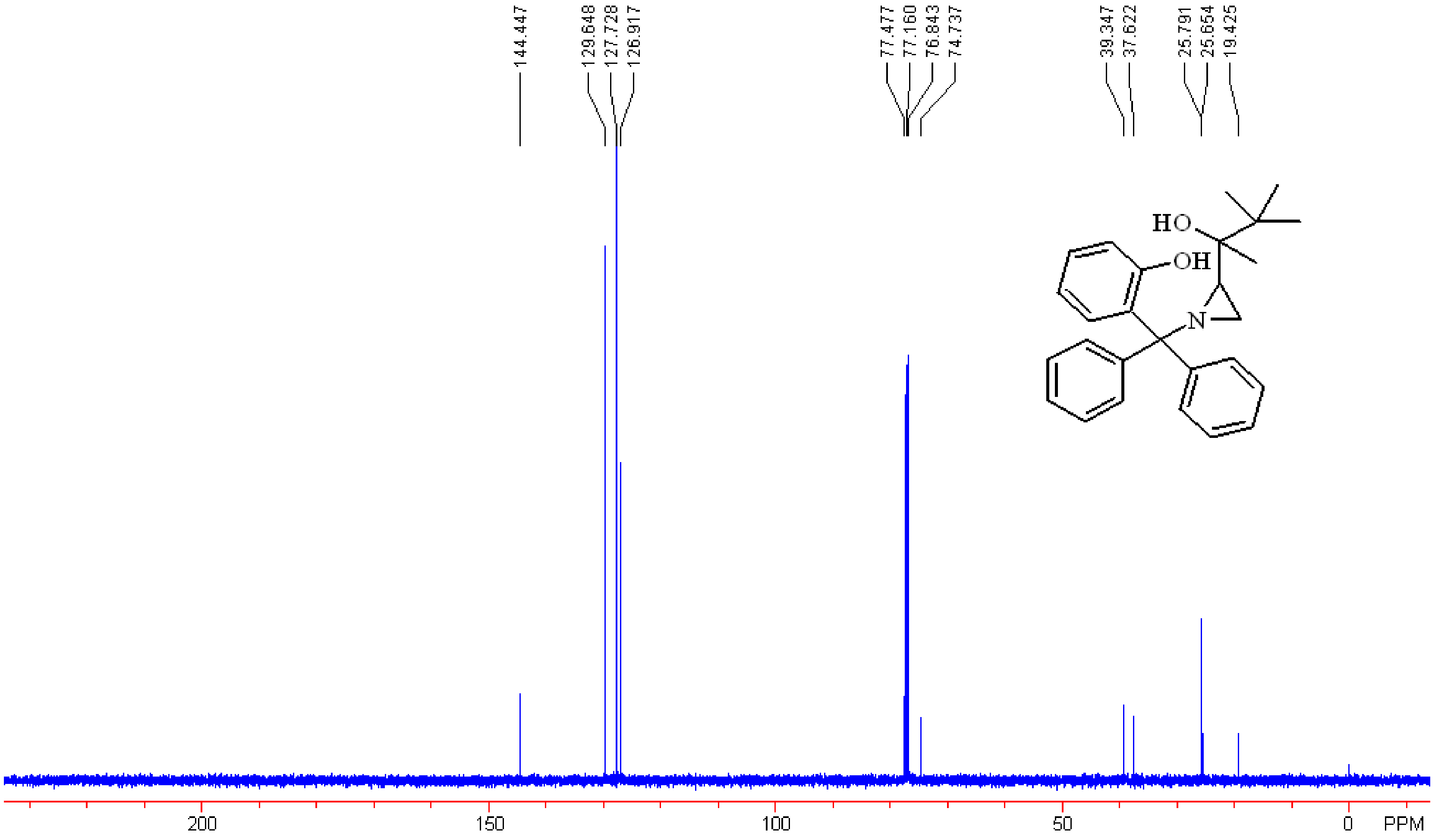

2-{[2-(1-Hydroxy-1,2,2-trimethyl-propyl)-aziridin-1-yl]diphenyl-methyl}phenol (mixture of diastereomers) 6d1

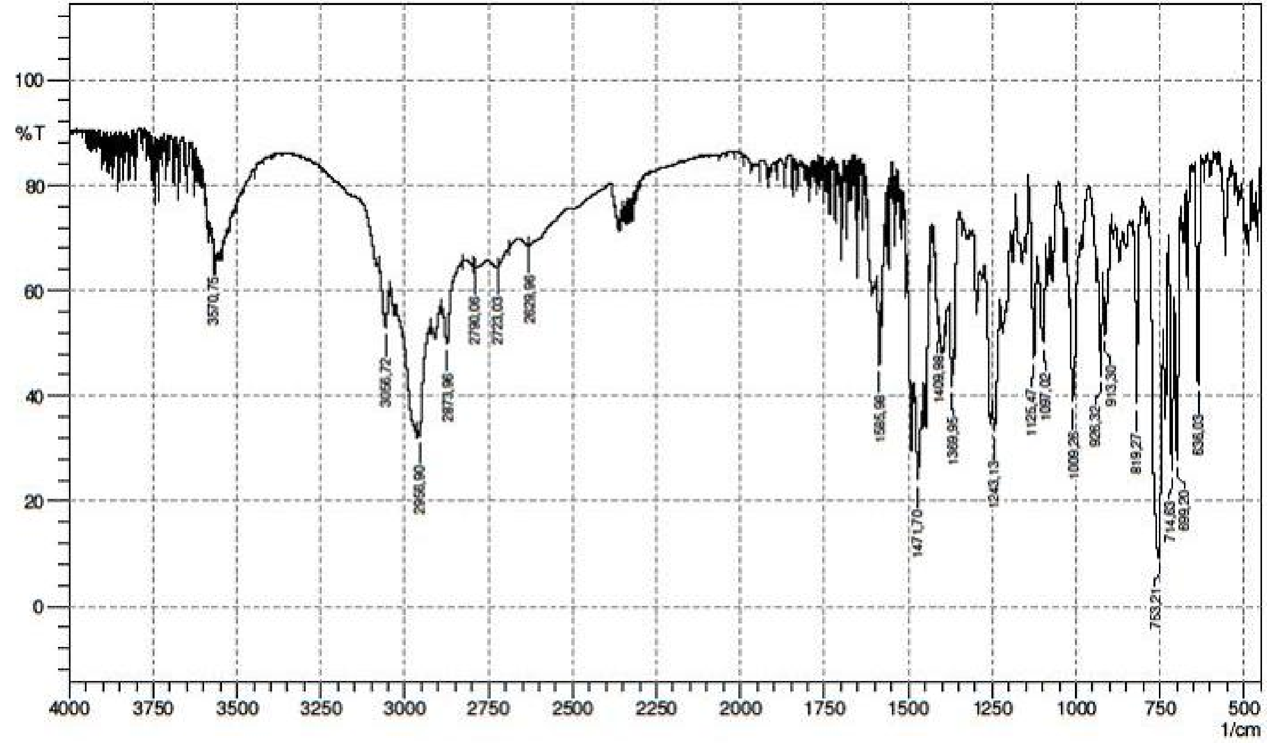

K-44

2-{[2-(1-Hydroxy-1,2,2-trimethyl-propyl)-aziridin-1-yl]diphenyl-methyl}phenol (mixture of diastereomers) 6d1

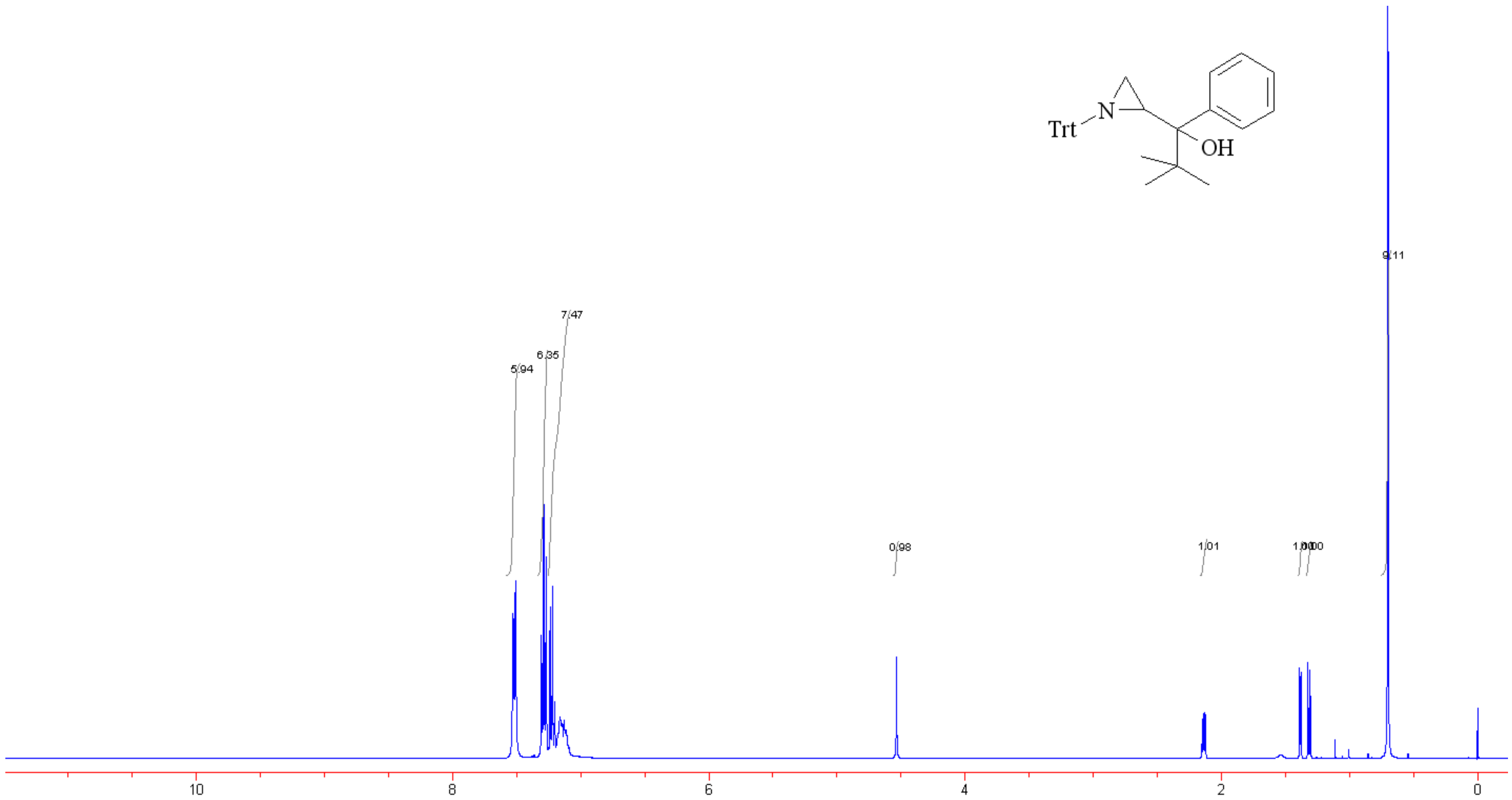

2,2-Dimethyl-1-phenyl-(1-triphenylmethylaziridine-2-yl)-propane-1-ol (mixture of diastereomers) 6e

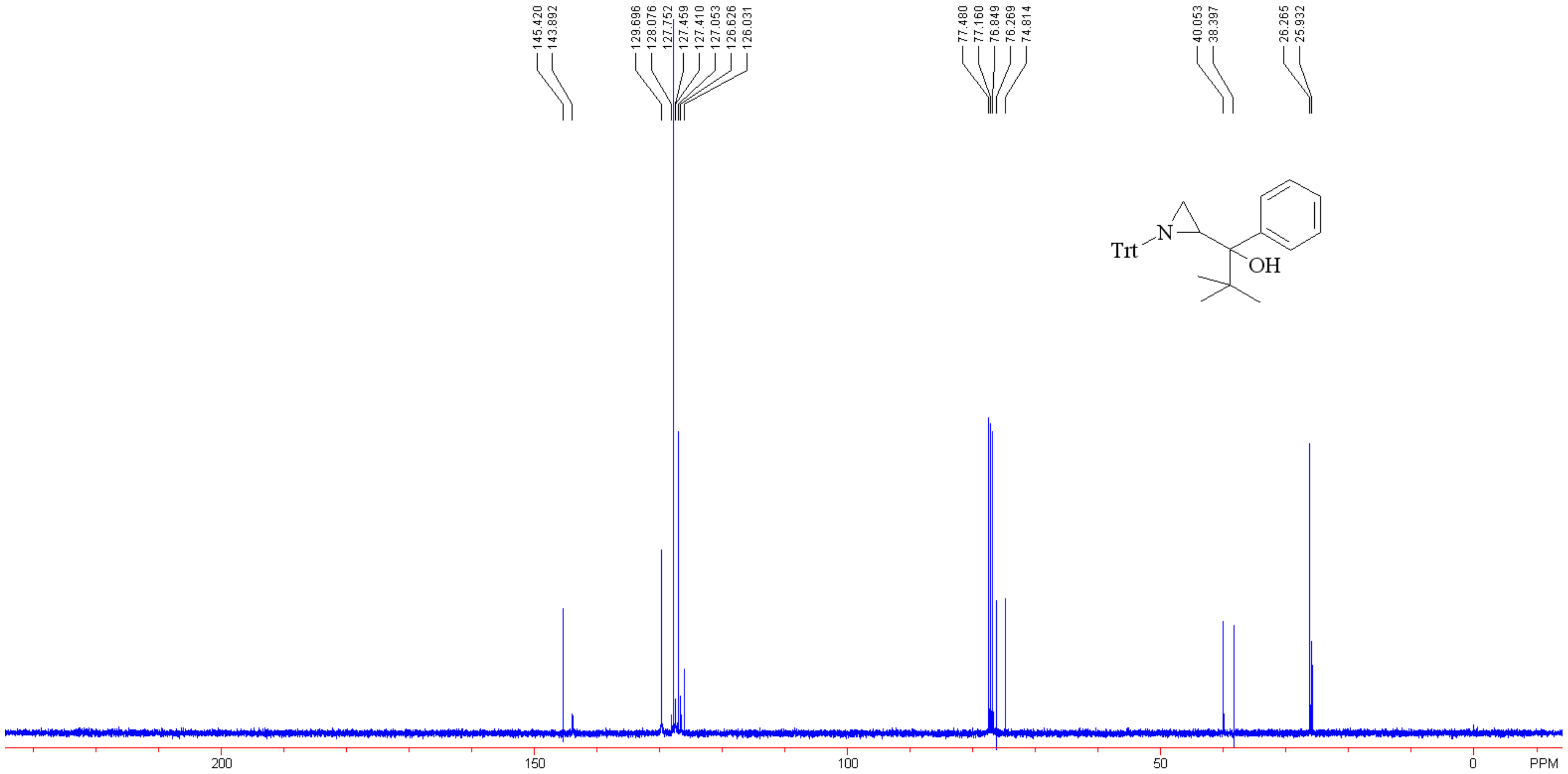

2,2-Dimethyl-1-phenyl-(1-triphenylmethylaziridine-2-yl)-propane-1-ol (mixture of diastereomers) 6e

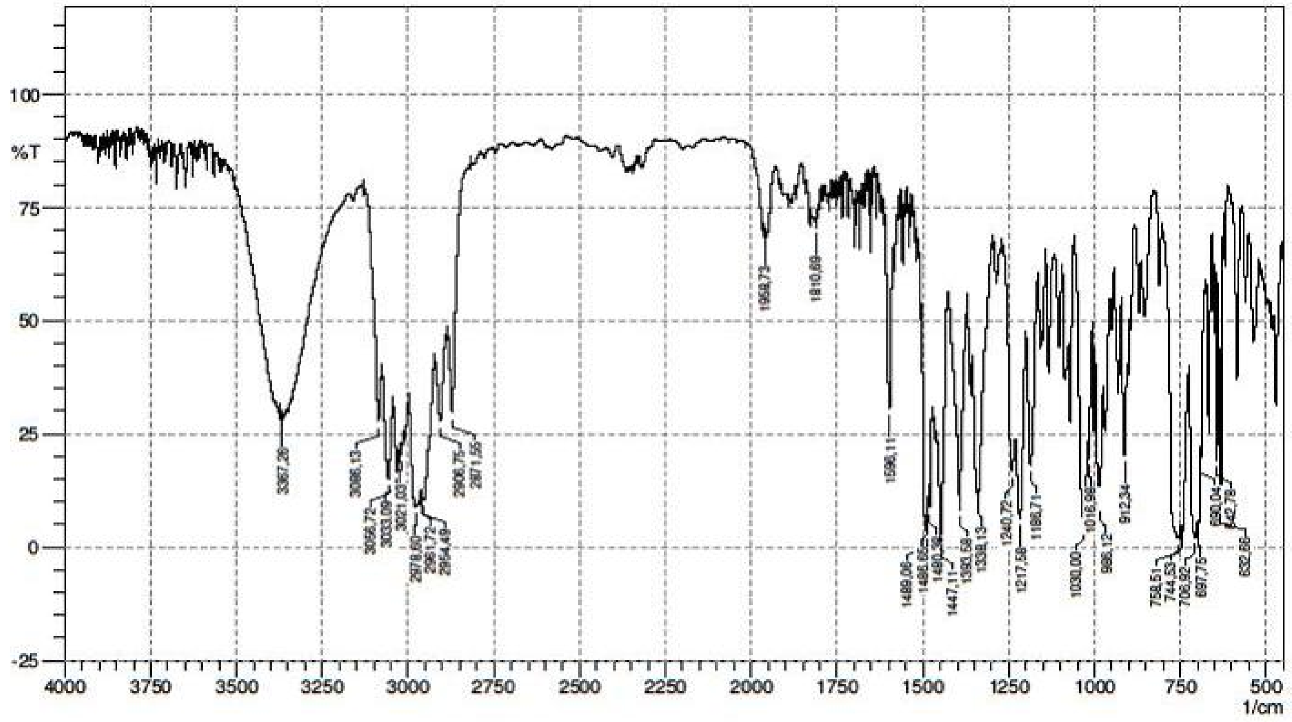

K45KD

2,2-Dimethyl-1-phenyl-(1-triphenylmethylaziridine-2-yl)-propane-1-ol (mixture of diastereomers) 6e

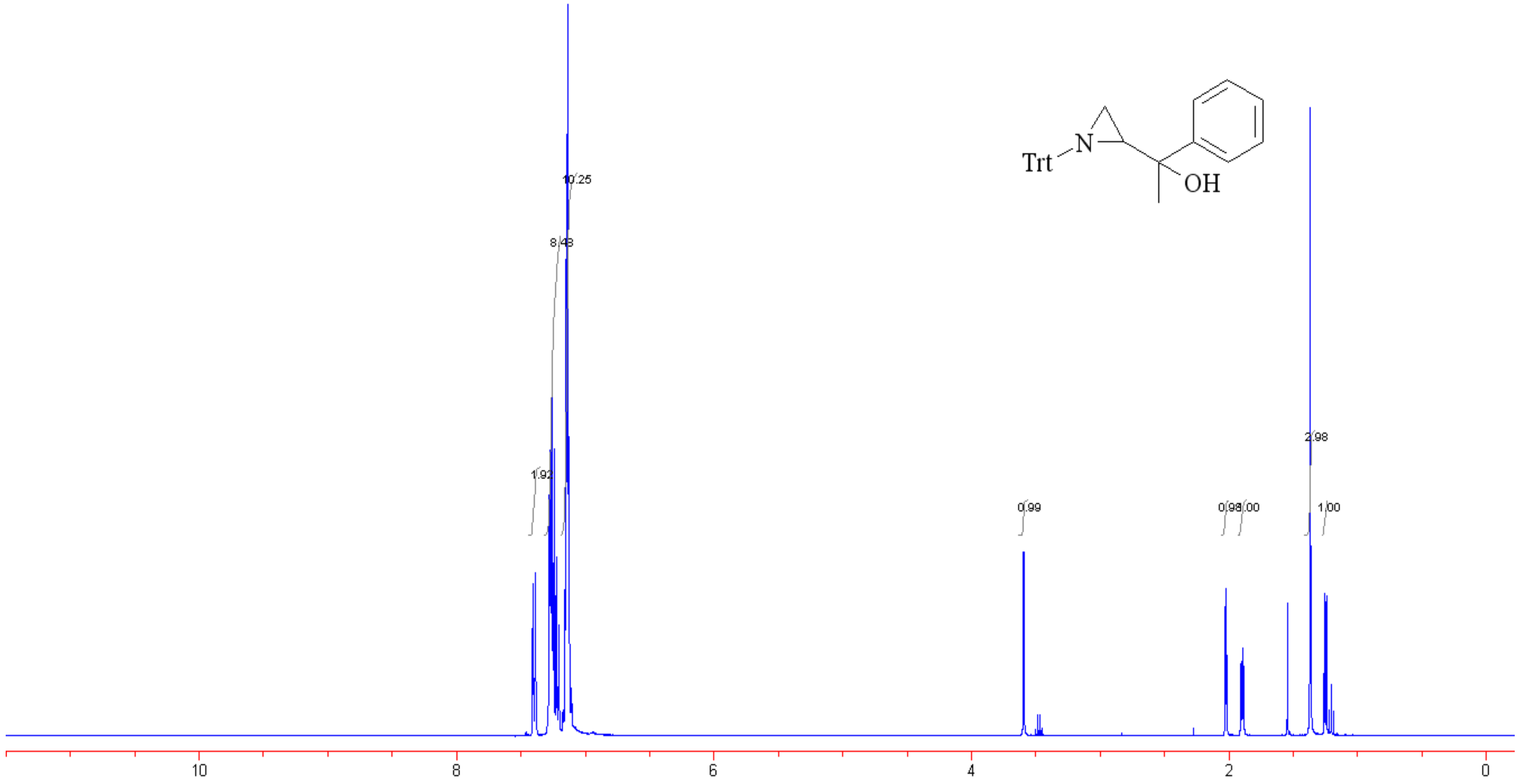

1-Phenyl-(1-triphenylmethylaziridine-2-yl)-ethanol (mixture of diastereomers) 6f

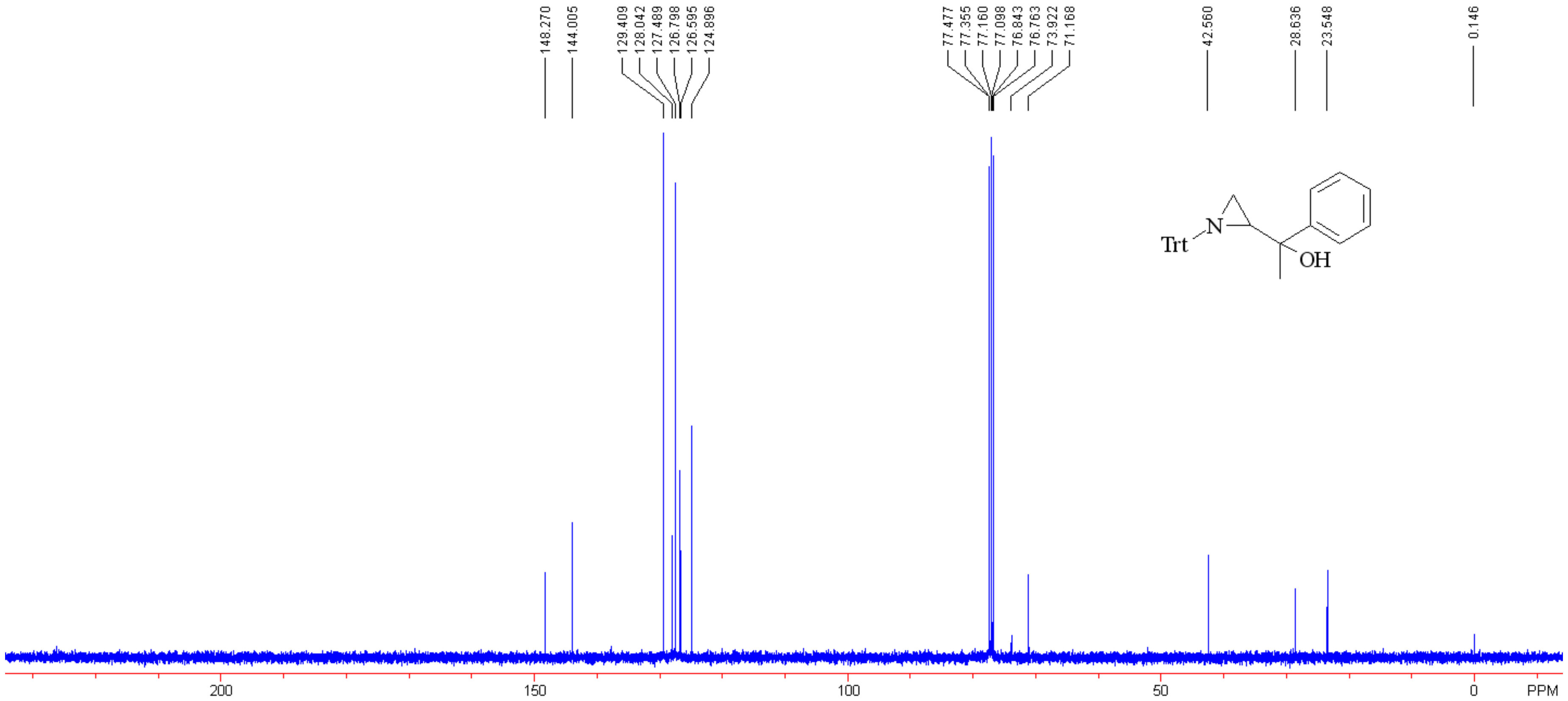

1-Phenyl-(1-triphenylmethylaziridine-2-yl)-ethanol (mixture of diastereomers)
6f

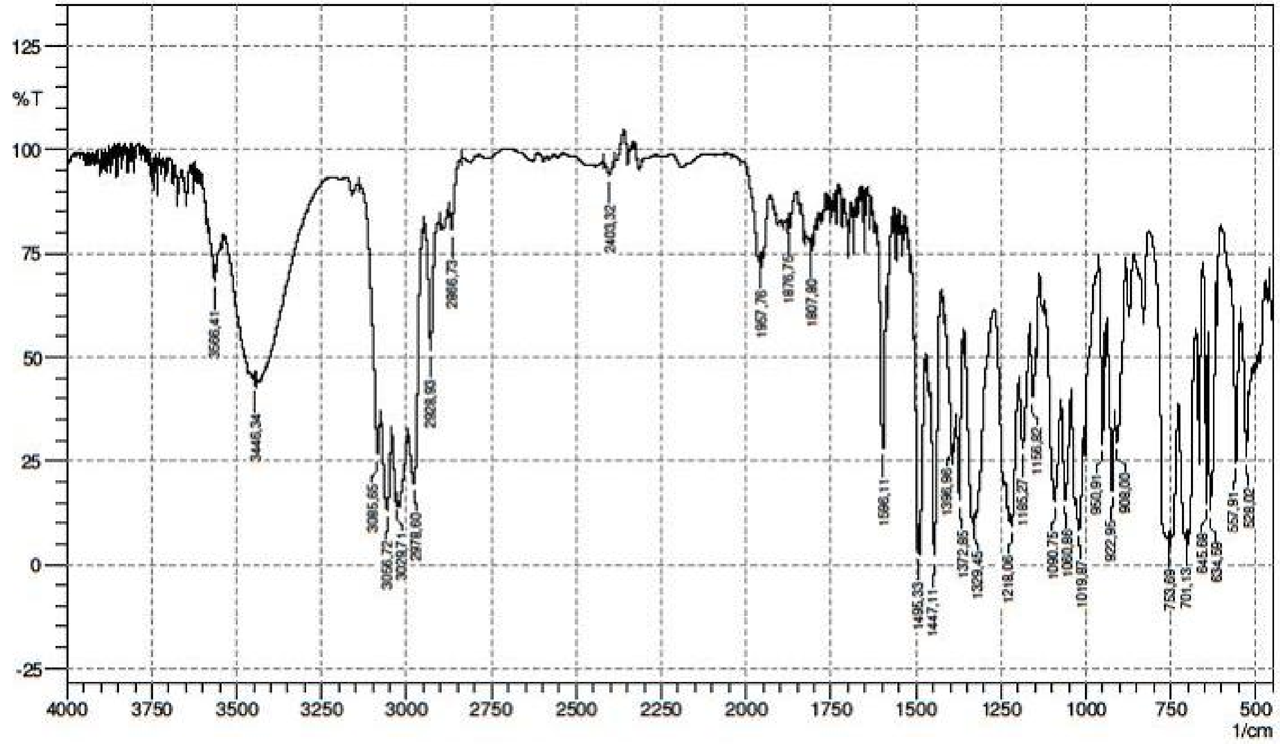

K42

1-Phenyl-(1-triphenylmethylaziridine-2-yl)-ethanol (mixture of diastereomers) 6f

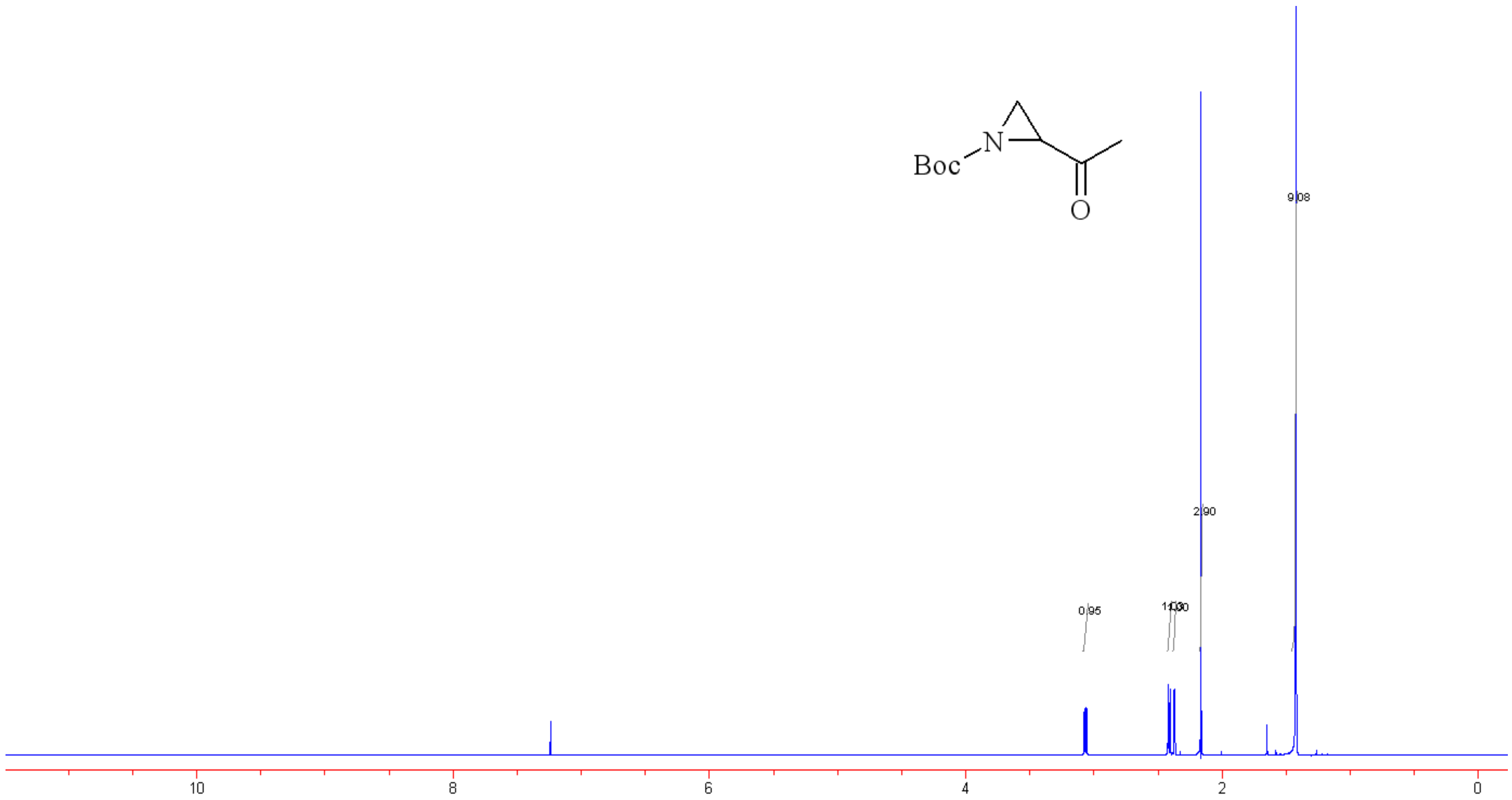

(1-*tert*-Butyloxycarbonylaziridine-2-yl)-methyl-methanone 7a

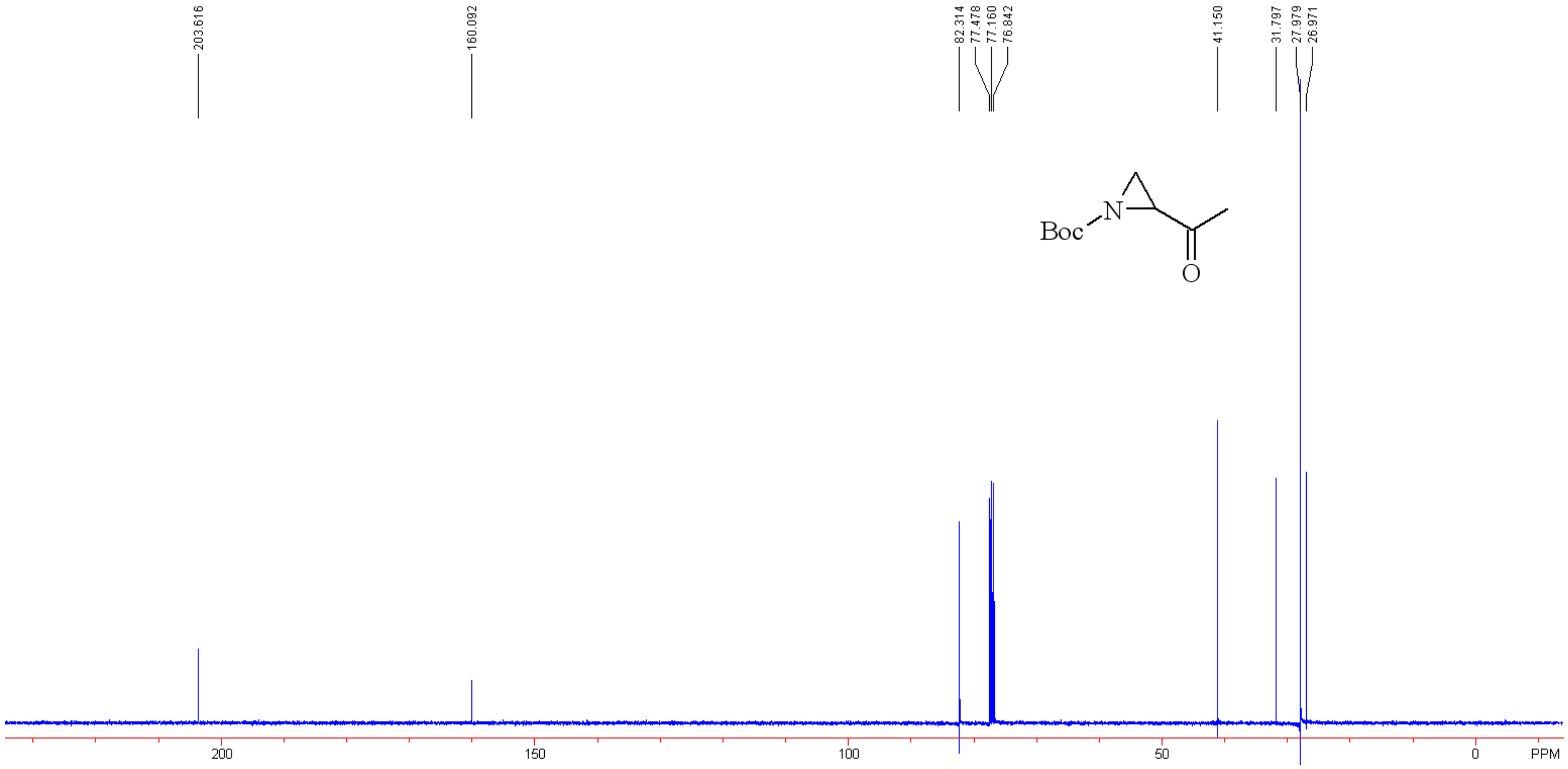

(1-*tert*-Butyloxycarbonylaziridine-2-yl)-methyl-methanone 7a

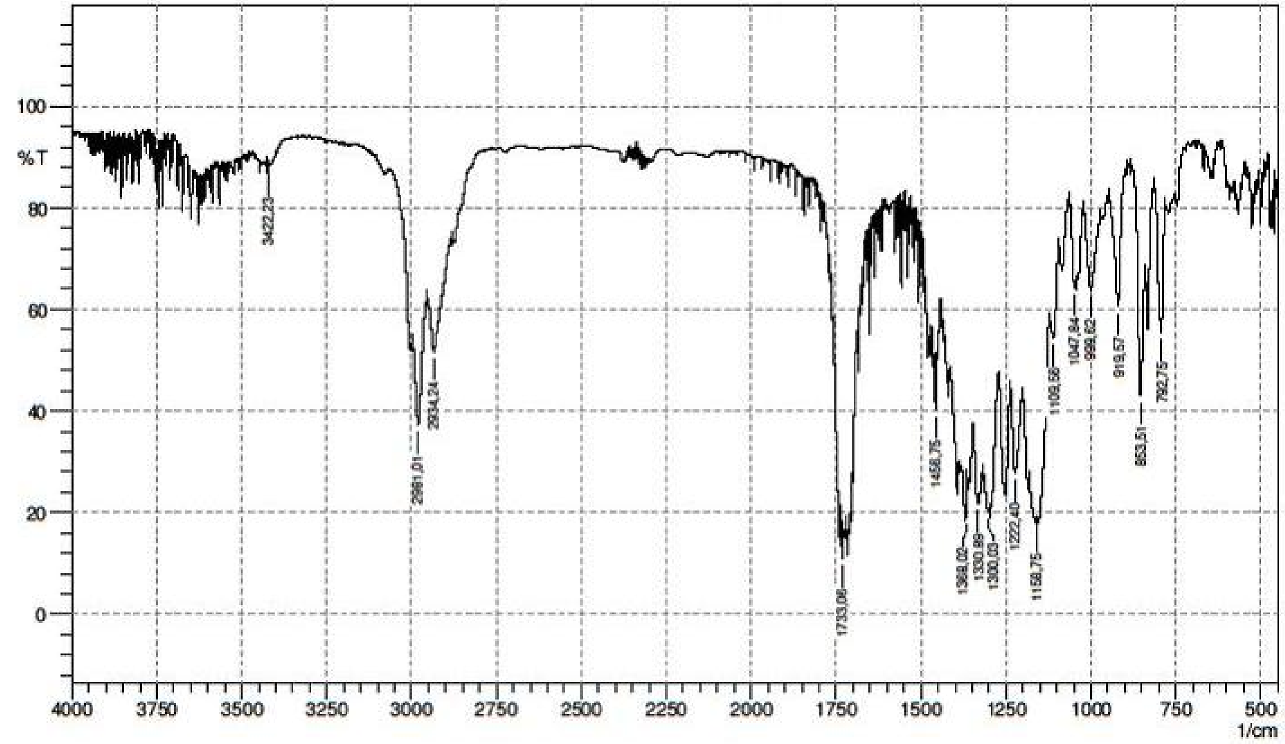

X1019F1

(1-*tert*-Butyloxycarbonylaziridine-2-yl)-methyl-methanone 7a

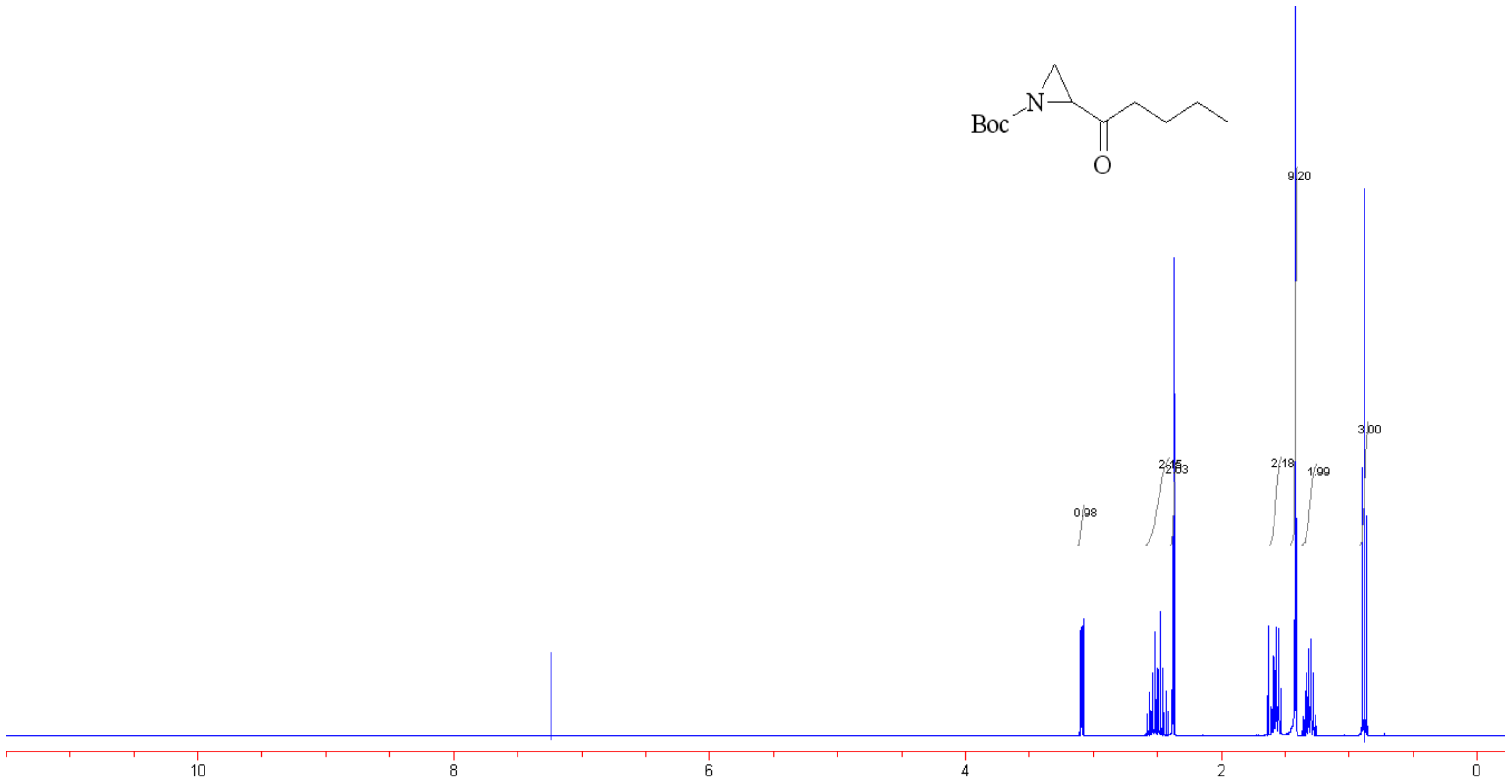

(1-*tert*-Butyloxycarbonylaziridine-2-yl)-n-butyl-methanone 7b

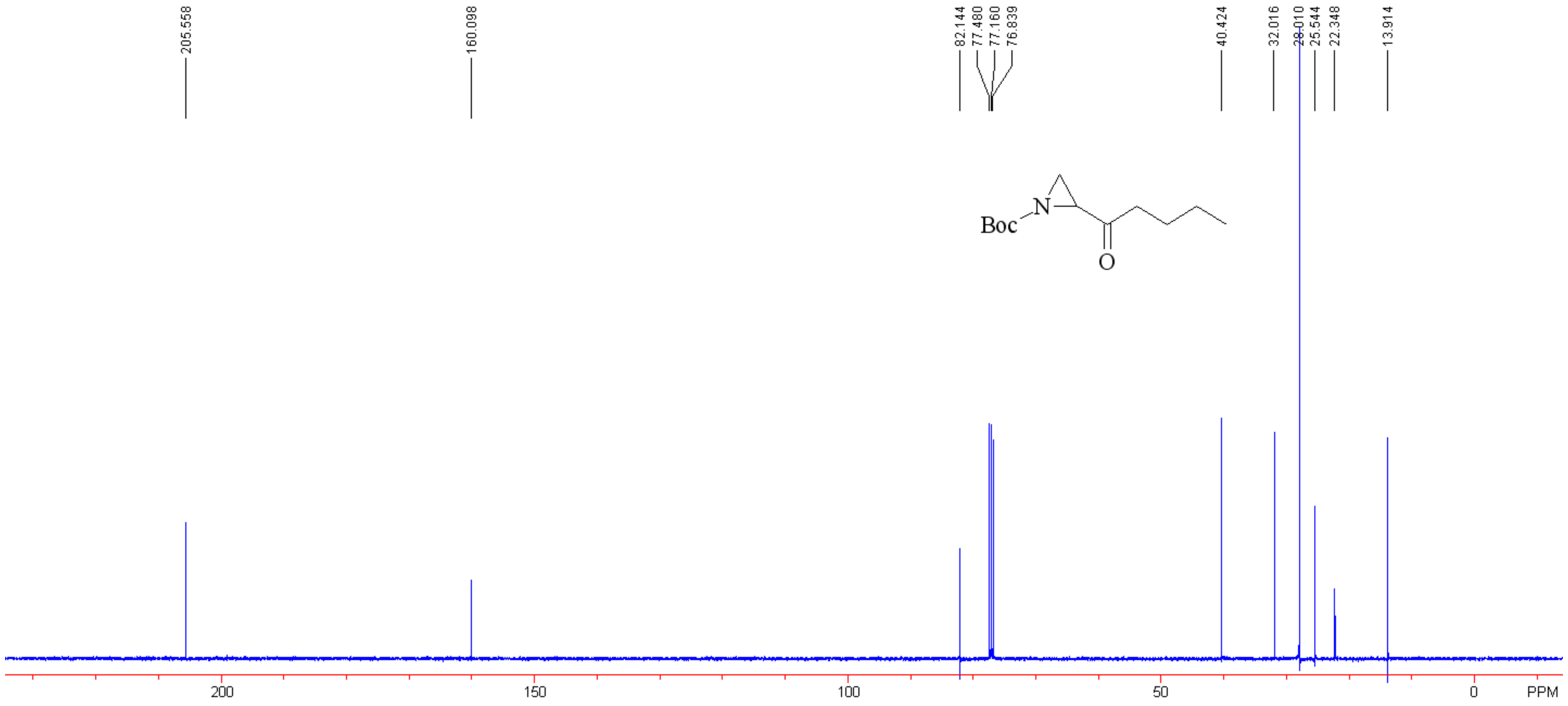

(1-*tert*-Butyloxycarbonylaziridine-2-yl)-n-butyl-methanone 7b

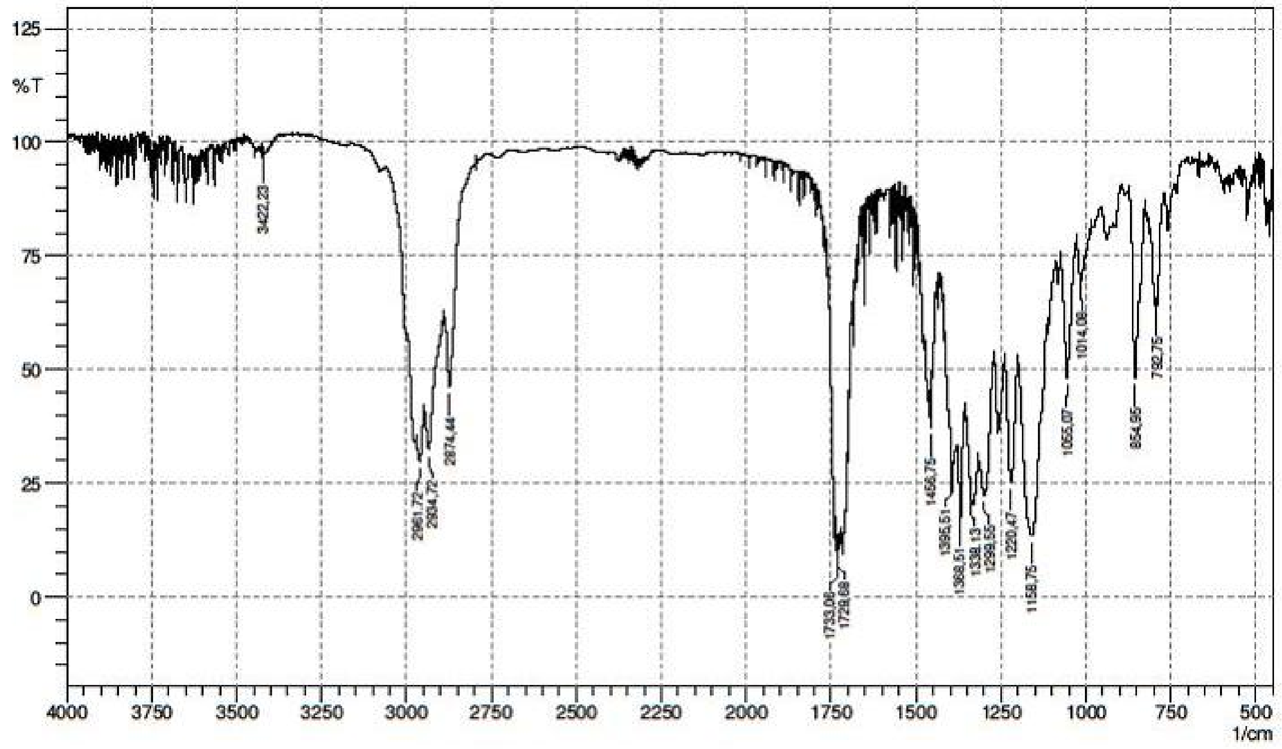

X1020F1

(1-*tert*-Butyloxycarbonylaziridine-2-yl)-n-butyl-methanone 7b

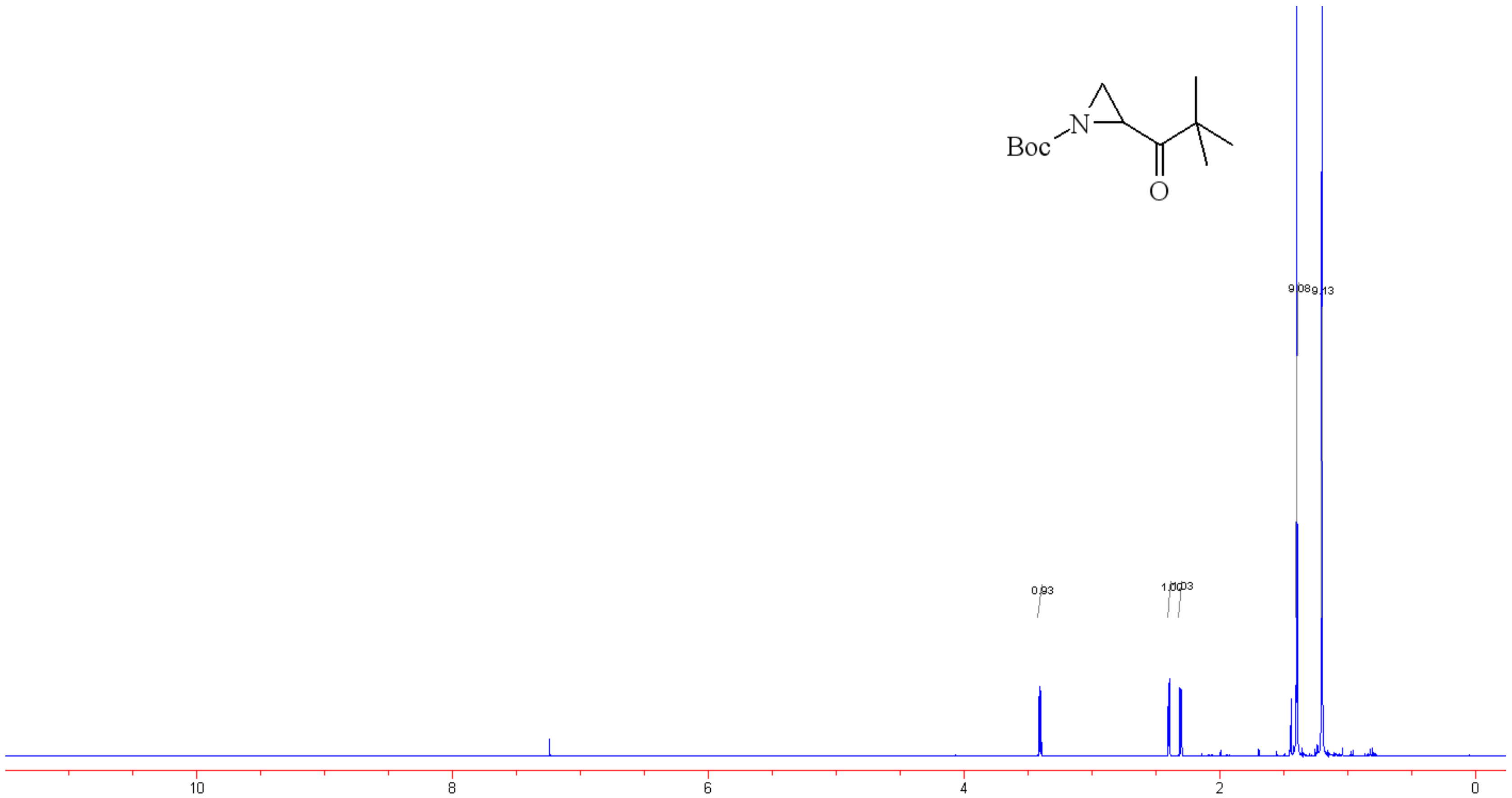

(1-*tert*-Butyloxycarbonylaziridine-2-yl)-t-butyl-methanone 7c

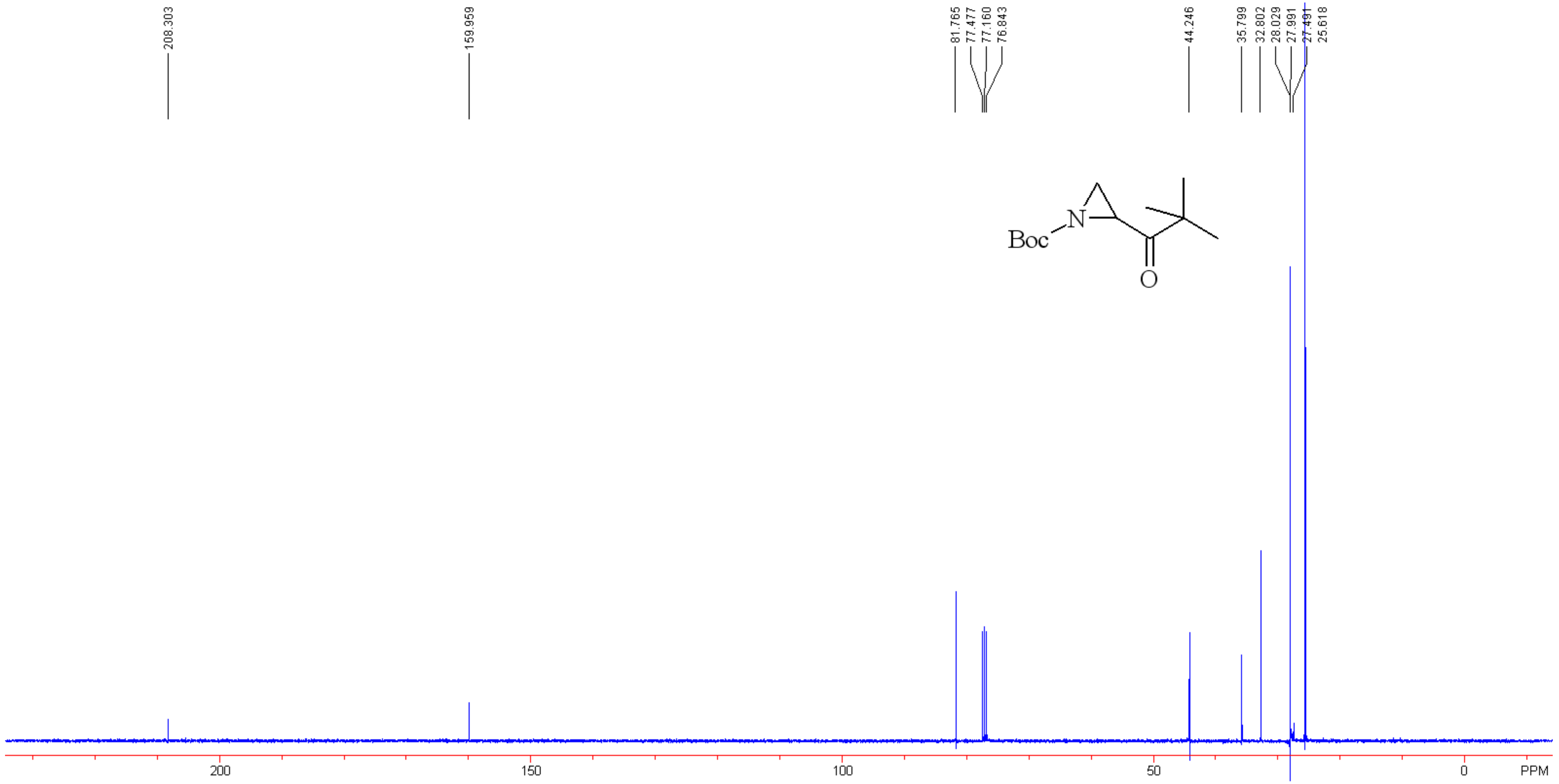

(1-*tert*-Butyloxycarbonylaziridine-2-yl)-t-butyl-methanone 7c

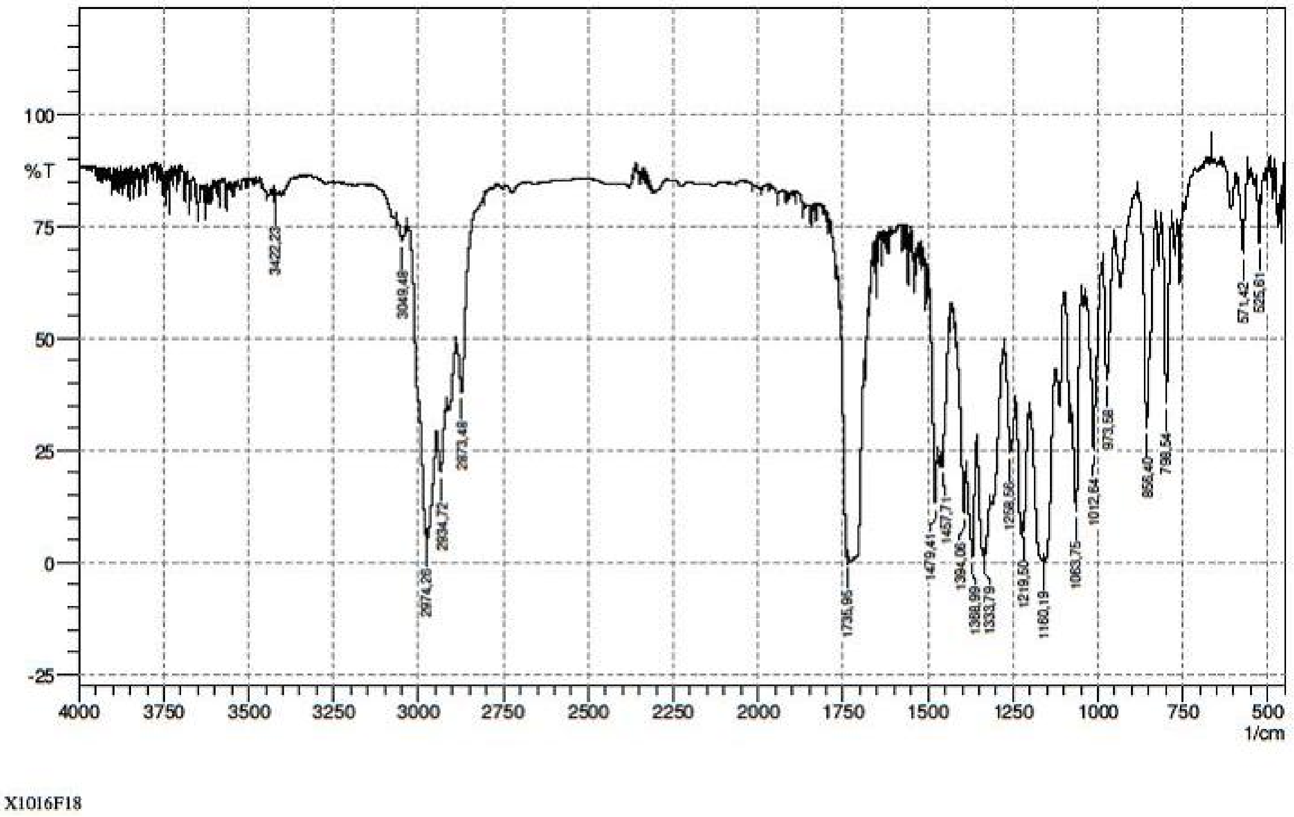

(1-*tert*-Butyloxycarbonylaziridine-2-yl)-t-butyl-methanone 7c

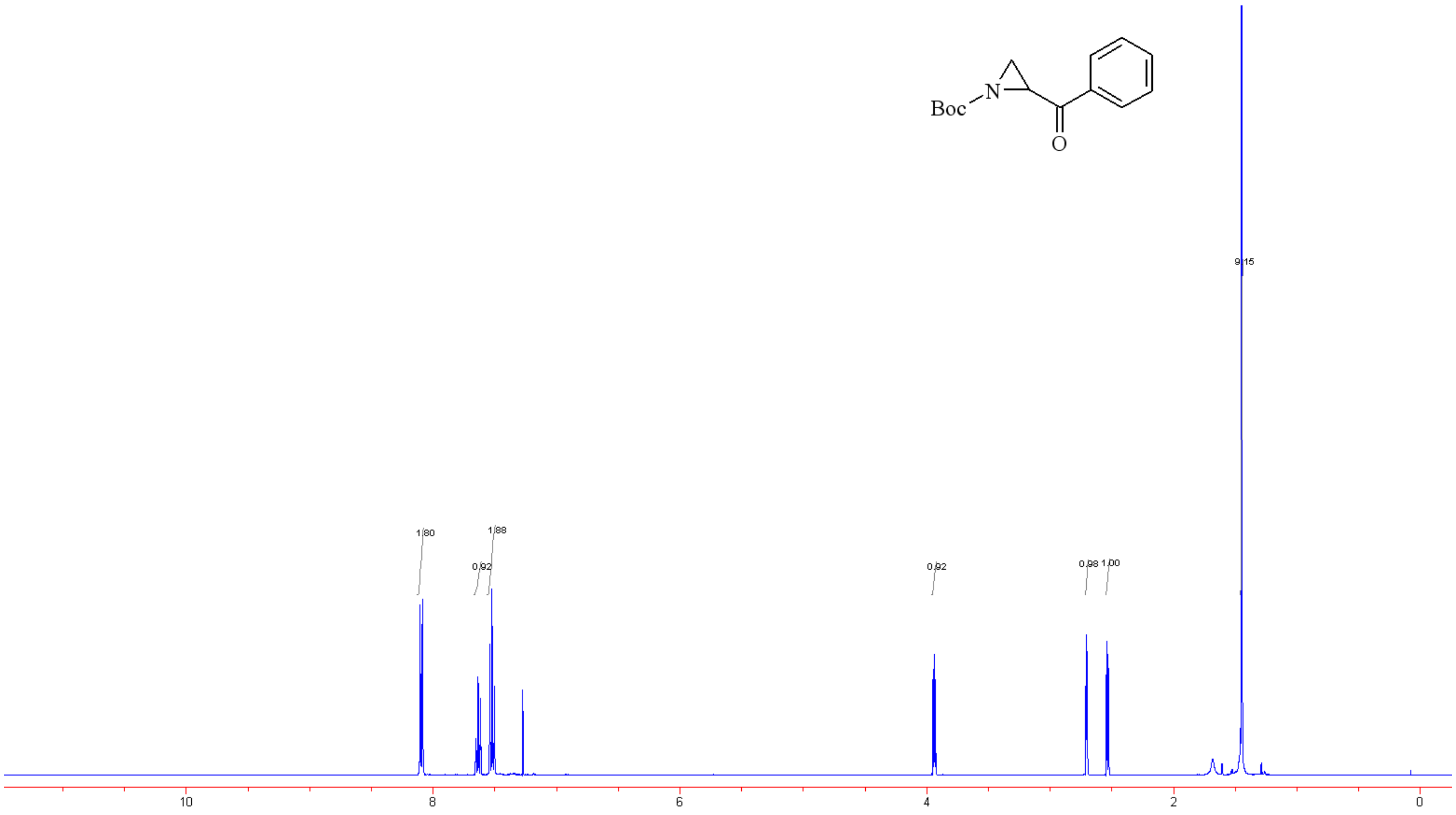

(1-*tert*-Butyloxycarbonylaziridine-2-yl)-phenyl-methanone 7d

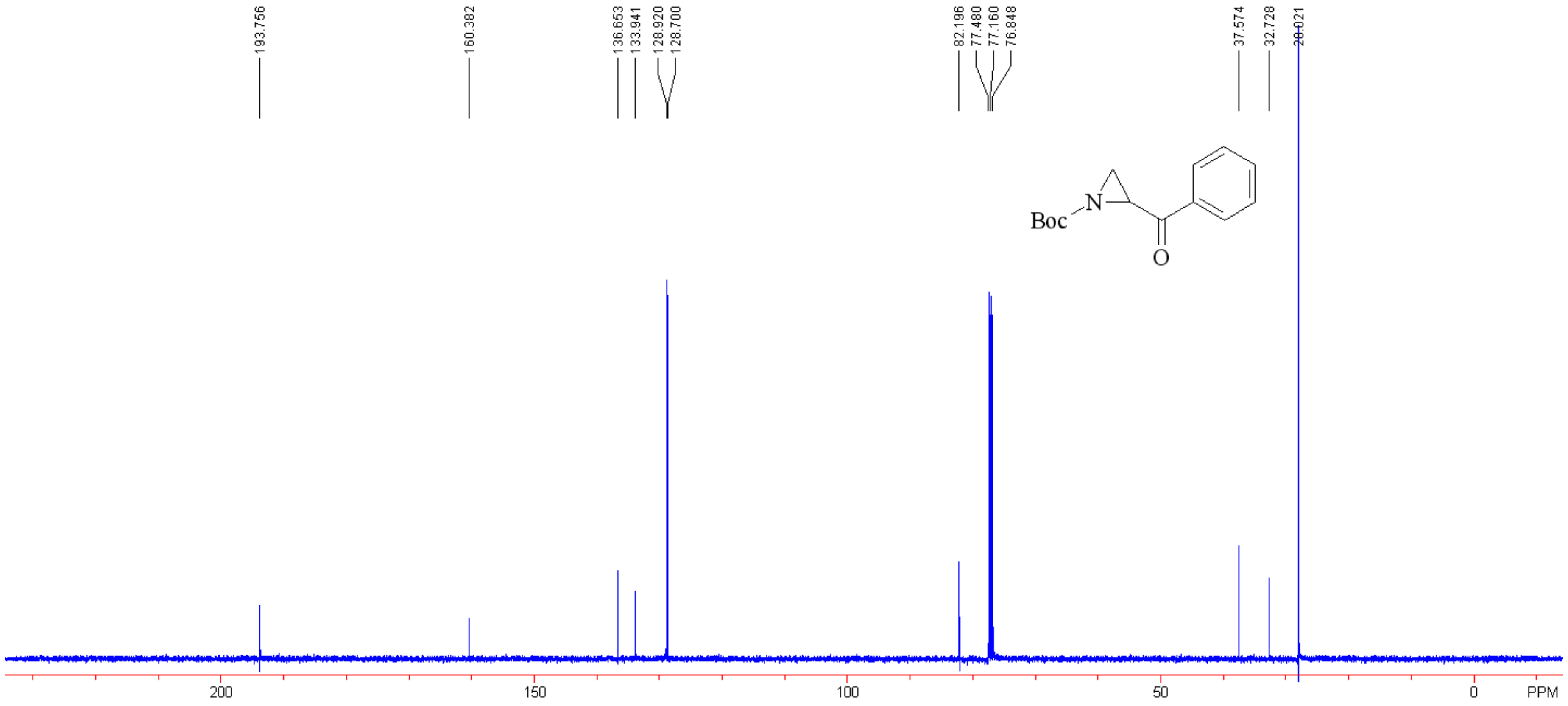

(1-*tert*-Butyloxycarbonylaziridine-2-yl)-phenyl-methanone 7d

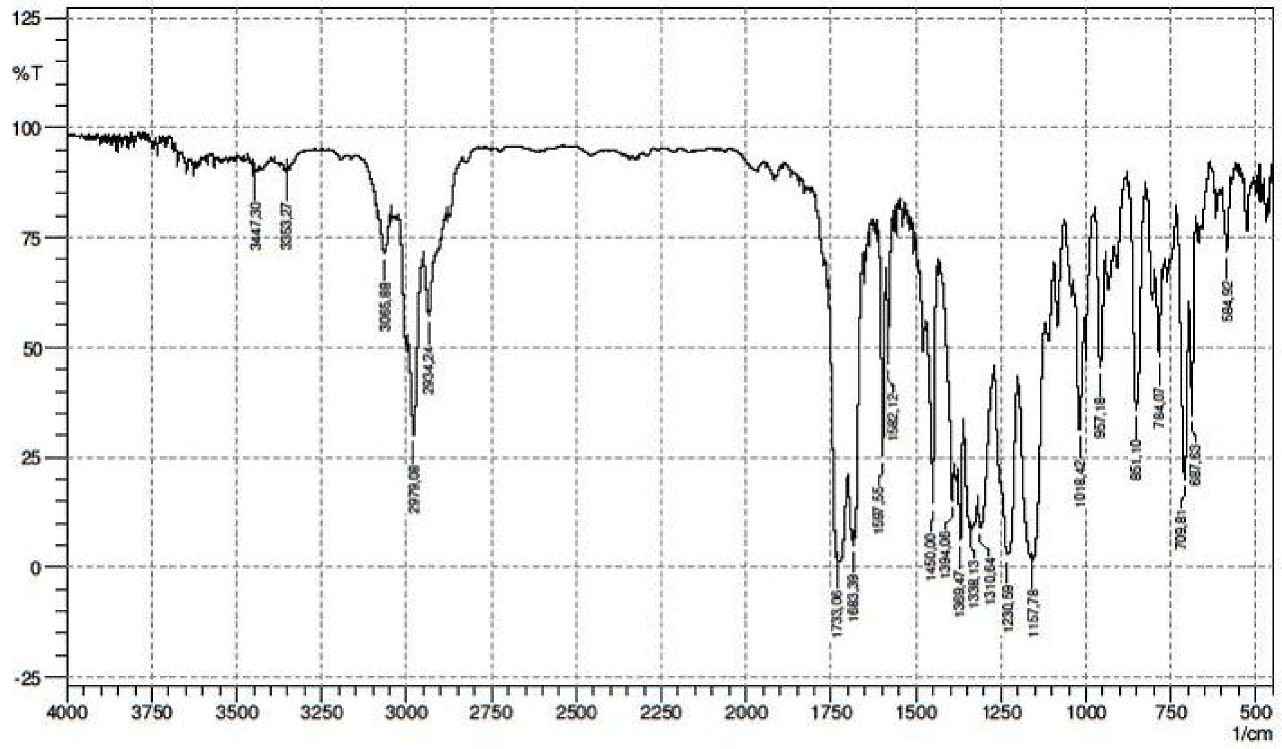

K110

(1-*tert*-Butyloxycarbonylaziridine-2-yl)-phenyl-methanone 7d

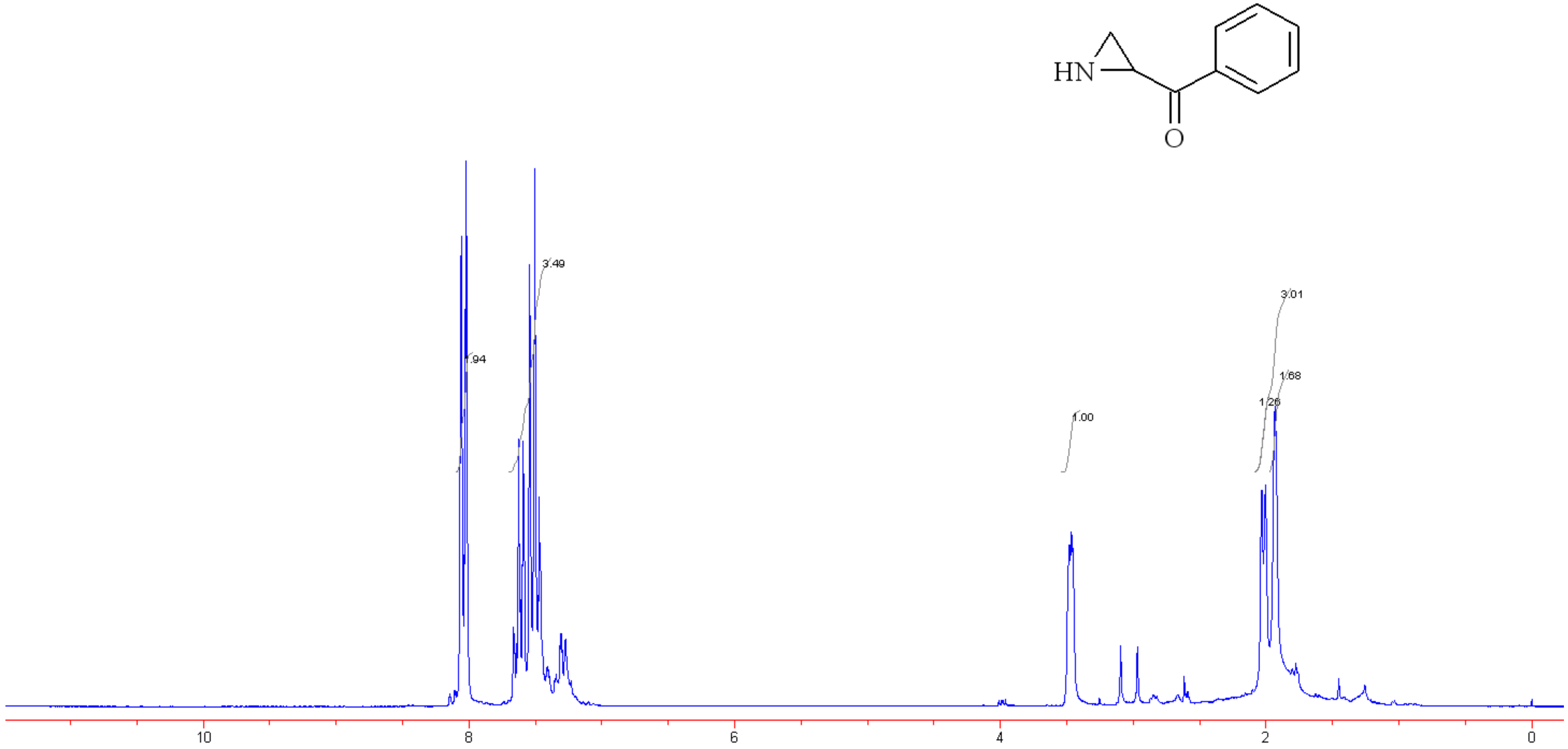

Aziridine-2-yl-phenyl-methanone 8

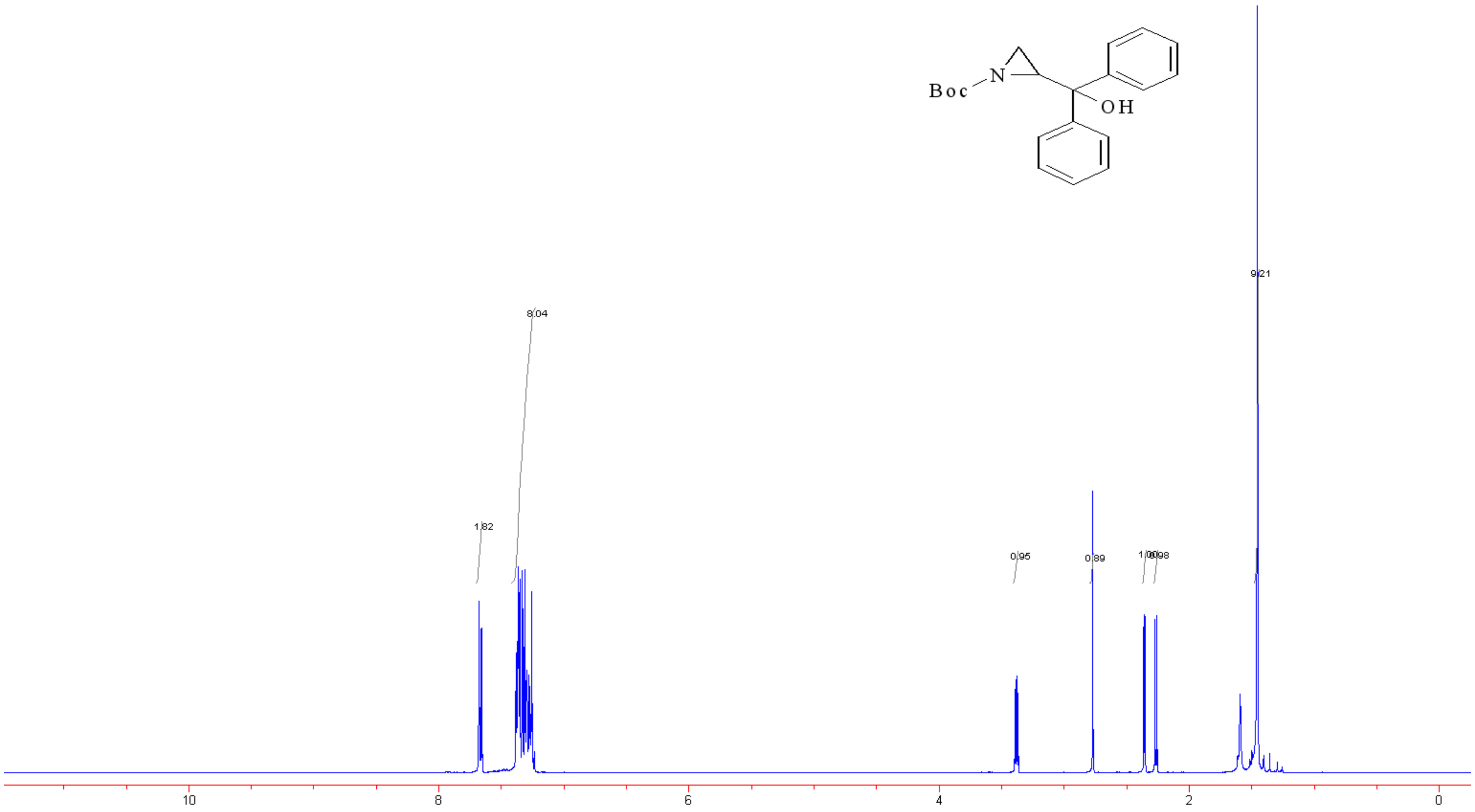

Diphenyl (1-*tert*-butyloxycarbonylaziridine-2-yl)-methanol 9

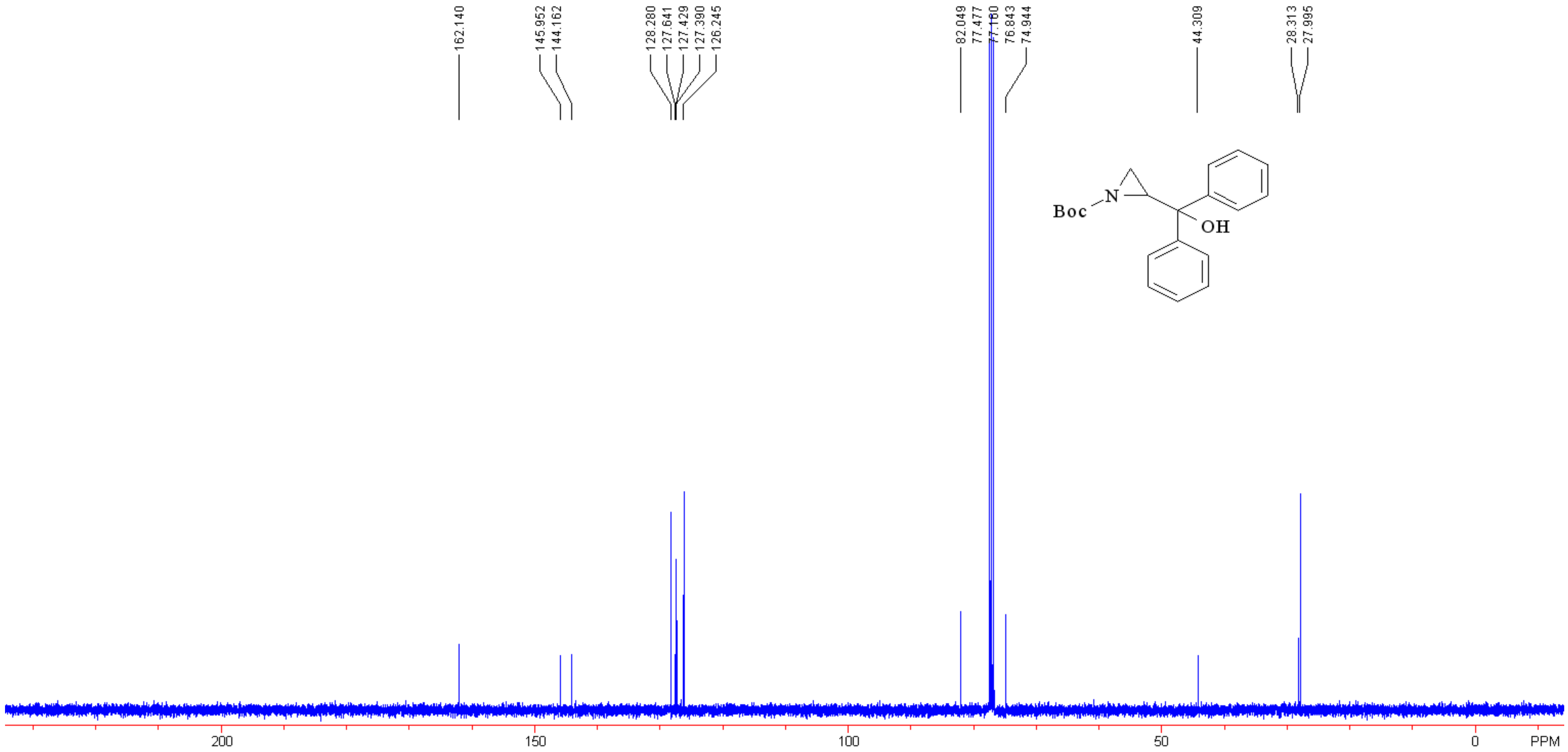

Diphenyl (1-*tert*-butyloxycarbonylaziridine-2-yl)-methanol 9

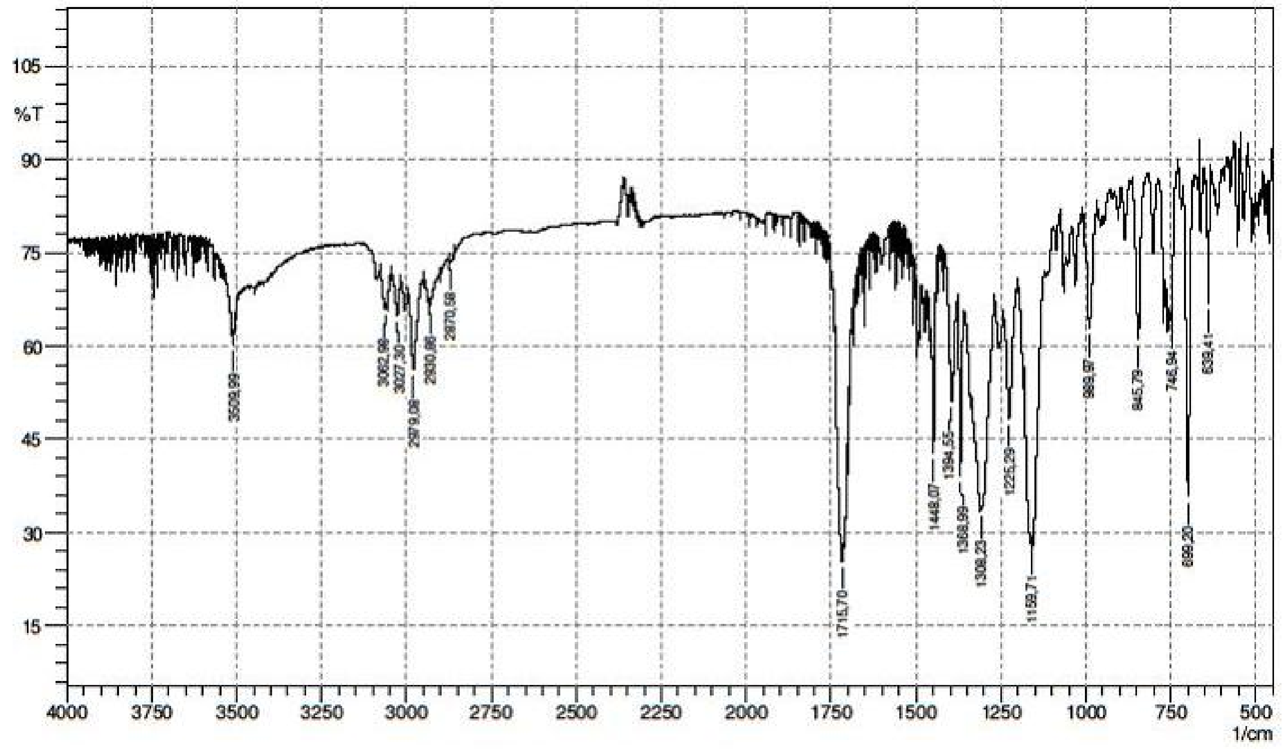

X1022A2

Diphenyl (1-*tert*-butyloxycarbonylaziridine-2-yl)-methanol 9

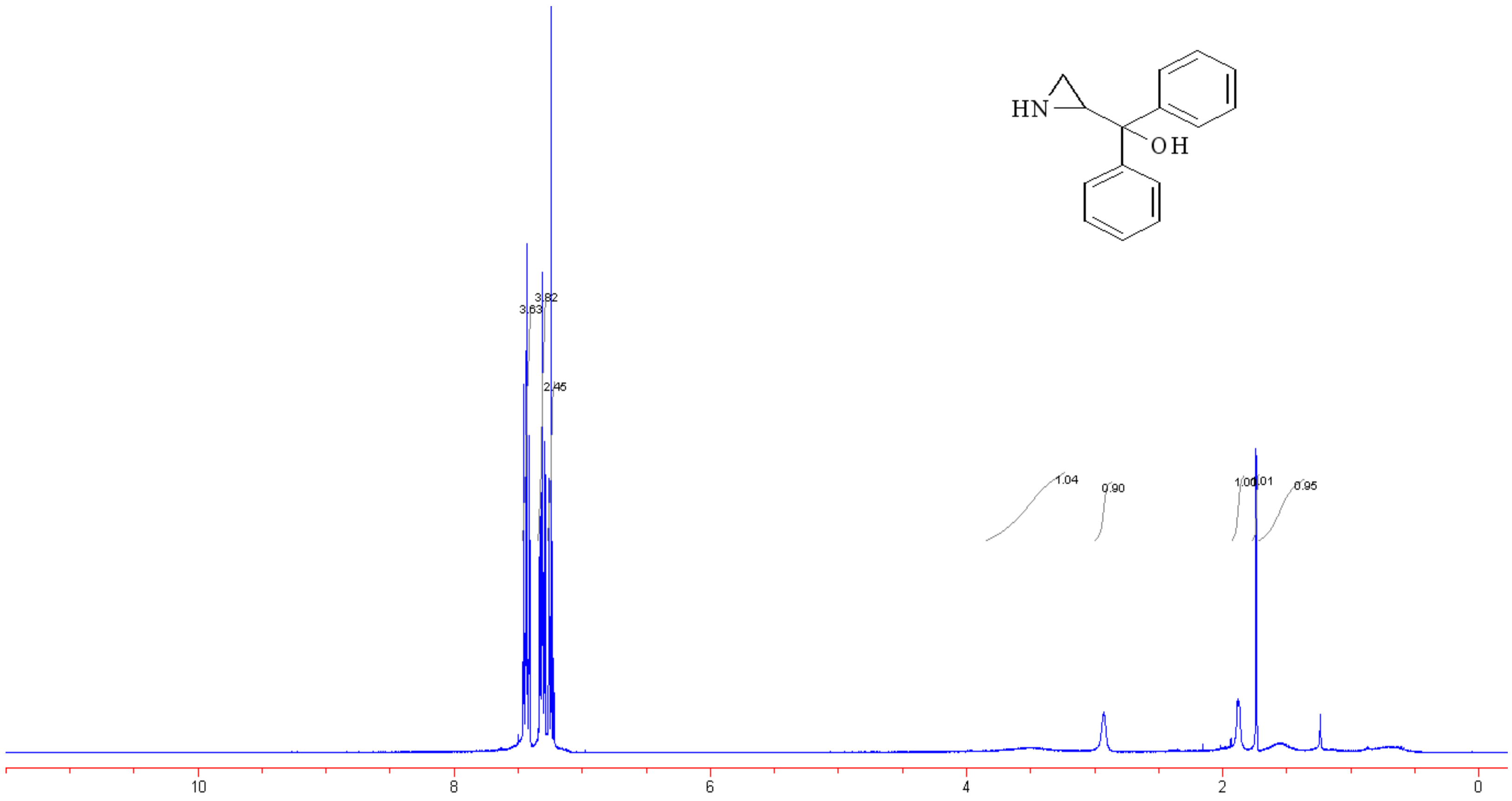

Aziridine-2-yl-diphenyl-methanol 10

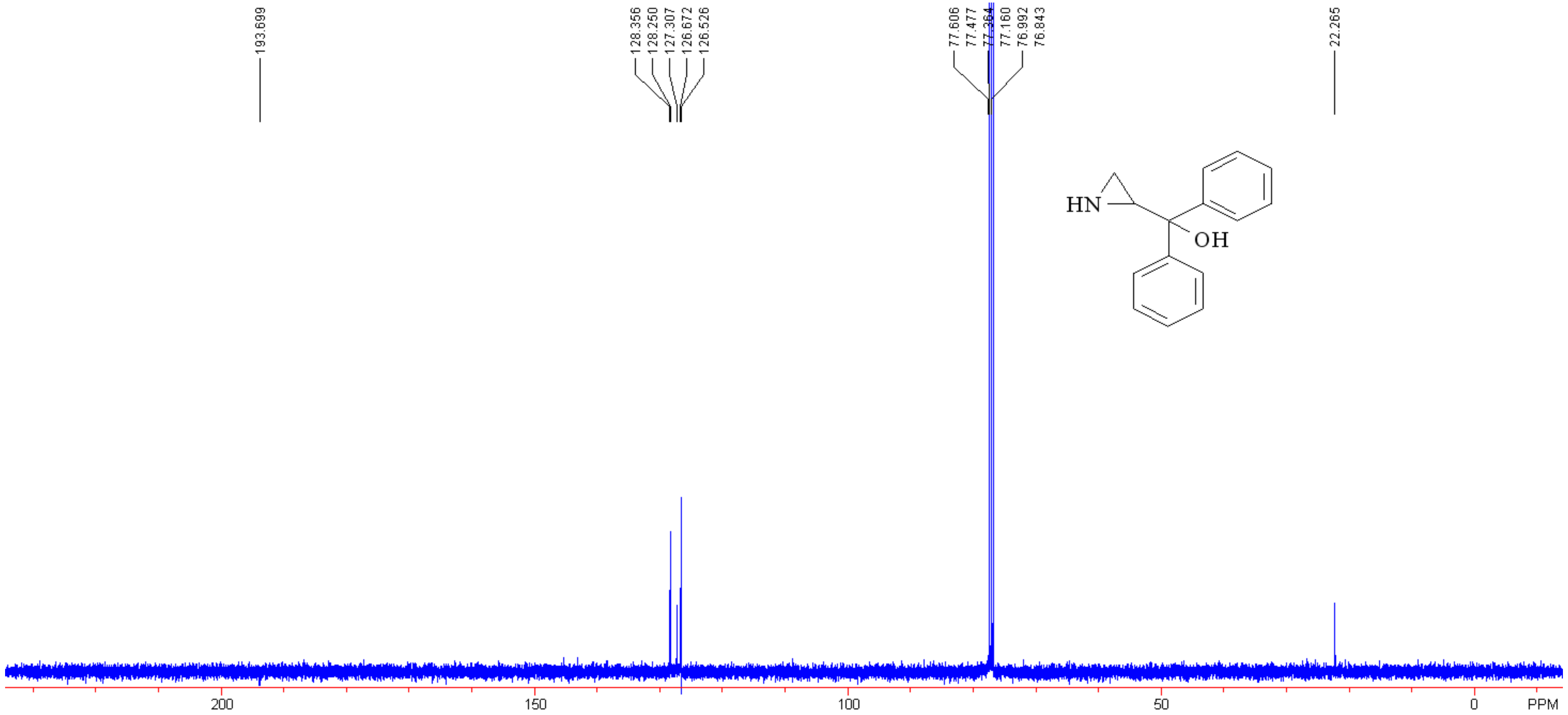

Aziridine-2-yl-diphenyl-methanol 10

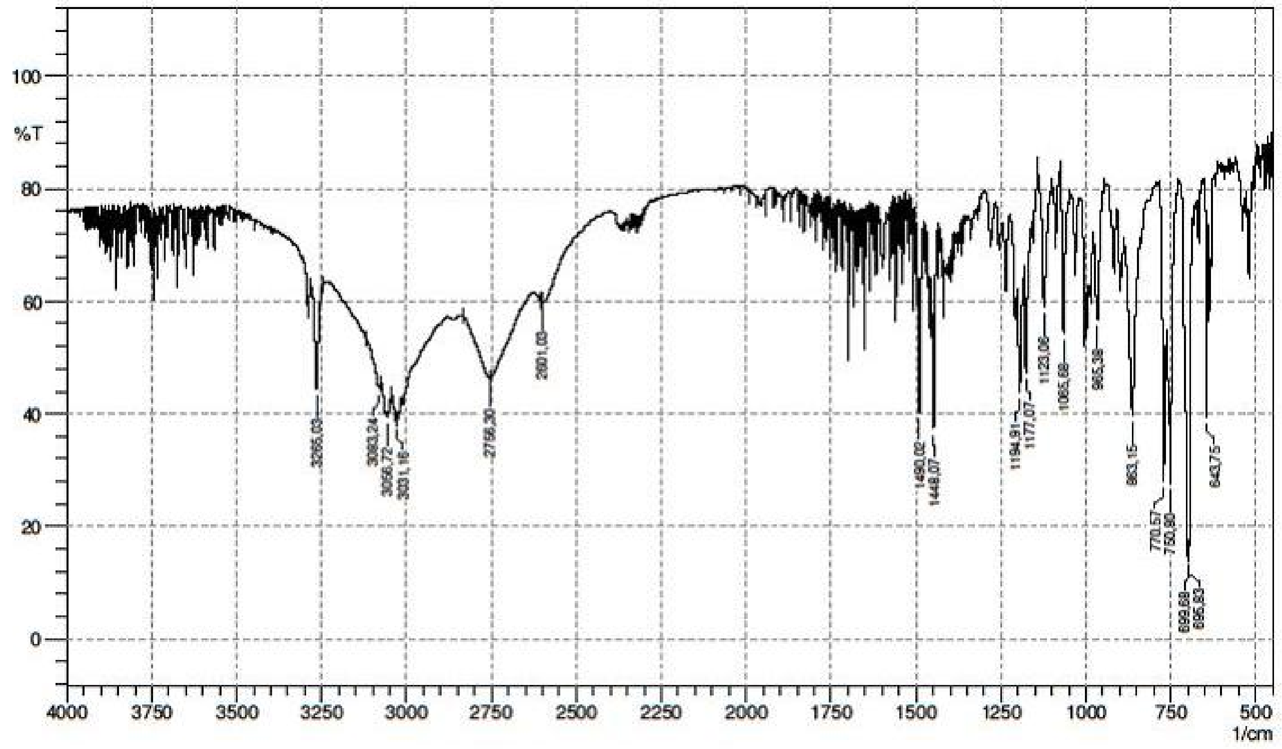

KK111HB

Aziridine-2-yl-diphenyl-methanol 10

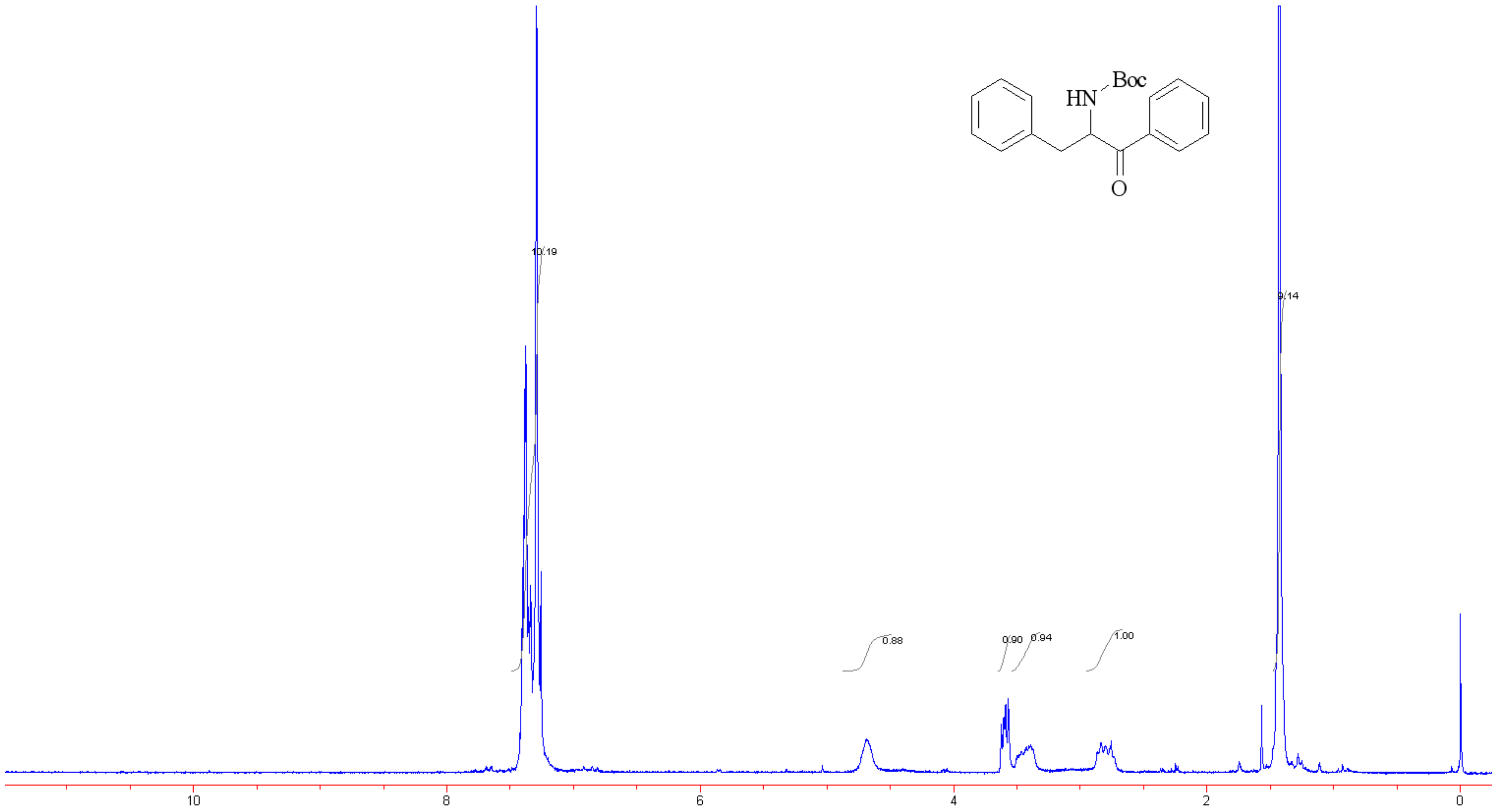

(1-Benzyl-2-oxo-2-phenyl-ethyl) carbaminic acid terc-butyl ester 11

Crystal data for compound 6c

Crystal data

$C_{30}H_{37}NO$	D_x = 1.139 Mg m^{-3}
M_r = 427.61	Mo *Kα* radiation, λ = 0.71073 Å
Monoclinic, *C*2/*c*	*Z* = 8
a = 25.1187 (8) Å	Cell parameters from 2983 reflections
b = 8.7502 (3) Å	θ = 0.9–27.5°
c = 23.5529 (8) Å	μ = 0.07 mm^{-1}
β = 105.5283 (15)°	*T* = 183 K
V = 4987.8 (3) Å^3	Needle, colourless
F(000) = 1856	0.41 × 0.12 × 0.07 mm

Data collection

Bruker-Nonius KappaCCD diffractometer	3227 reflections with $I > 2\sigma(I)$
Radiation source: fine-focus sealed tube	R_{int} = 0.068
Graphite monochromator	$2\theta_{max}$ = 56°,
φ and ω scan	h = −32 32
5848 independent reflections	k = −11 10
9910 measured reflections	l = −31 30

Refinement

Refinement on F^2	Secondary atom site location: difference Fourier map
Least-squares matrix: full	Hydrogen site location: mixed
$R[F^2 > 2\sigma(F^2)]$ = 0.075	H atoms treated by a mixture of independent and constrained refinement
$wR(F^2)$ = 0.166	$w = 1/[\sigma^2(F_o^2) + (0.0484P)^2 + 5.9402P]$ where $P = (F_o^2 + 2F_c^2)/3$
S = 1.01	$(\Delta/\sigma)_{max}$ < 0.001
5848 reflections	$\Delta\rho_{max}$ = 0.25 e Å^{-3}
297 parameters	$\Delta\rho_{min}$ = −0.23 e Å^{-3}
0 restraints	Extinction correction: none
Primary atom site location: structure-invariant direct methods	

Refinement of F^2 against ALL reflections. The weighted R-factor wR and goodness of fit S are based on F^2, conventional R-factors R are based on F, with F set to zero for negative F^2. The threshold expression of $F^2 > 2sigma(F^2)$ is used only for calculating R-factors(gt) etc. and is not relevant to the choice of reflections for refinement. R-factors based on F^2 are statistically about twice as large as those based on F, and R-factors based on ALL data will be even larger.

Fractional atomic coordinates and isotropic or equivalent isotropic displacement parameters (Å^2)

	x	y	z	U_{iso}*/U_{eq}
O24	0.73475 (8)	−0.19275 (19)	0.04706 (7)	0.0297 (4)
H24	0.7653 (12)	−0.201 (3)	0.0415 (12)	0.038 (8)*
N1	0.83566 (8)	−0.0479 (2)	0.08281 (8)	0.0250 (5)
C17	0.92779 (10)	−0.1677 (3)	0.12339 (10)	0.0243 (5)
C4	0.89599 (9)	−0.0149 (2)	0.11110 (10)	0.0234 (5)
C22	0.91048 (10)	−0.2915 (3)	0.08549 (11)	0.0276 (6)
H22	0.8762	−0.2863	0.0553	0.033*
C5	0.90302 (10)	0.0850 (3)	0.16613 (10)	0.0273 (6)
C2	0.79079 (10)	−0.0057 (3)	0.11037 (11)	0.0264 (6)
H2	0.8034 (10)	0.046 (3)	0.1483 (11)	0.031 (7)*
C23	0.74100 (10)	−0.1122 (3)	0.10118 (10)	0.0247 (5)
C16	0.89453 (10)	0.0328 (3)	0.00377 (11)	0.0295 (6)
H16	0.8613	−0.0268	−0.0080	0.035*
C18	0.97778 (11)	−0.1804 (3)	0.16610 (11)	0.0315 (6)
H18	0.9928	−0.0936	0.1941	0.038*
C11	0.91919 (10)	0.0595 (2)	0.06333 (10)	0.0247 (5)
C12	0.96818 (10)	0.1430 (3)	0.07860 (12)	0.0316 (6)
H12	0.9898	0.1527	0.1216	0.038*
C3	0.80065 (10)	0.0848 (3)	0.06083 (11)	0.0324 (6)
H3A	0.8153	0.1860	0.0729	0.039*
H3B	0.7760	0.0812	0.0189	0.039*
C15	0.91721 (12)	0.0912 (3)	−0.03920 (12)	0.0372 (7)
H15	0.8996	0.0718	−0.0800	0.045*
C29	0.68688 (10)	−0.0098 (3)	0.09175 (10)	0.0322 (6)
C25	0.75166 (11)	−0.2379 (3)	0.15189 (11)	0.0369 (7)
C10	0.90218 (11)	0.2440 (3)	0.16216 (12)	0.0358 (6)
H10	0.8999	0.2911	0.1248	0.043*
C21	0.94187 (11)	−0.4229 (3)	0.09116 (11)	0.0333 (6)

H21	0.9292	−0.5062	0.0644	0.040*
C20	0.99056 (12)	−0.4350 (3)	0.13479 (12)	0.0389 (7)
H20	1.0140	−0.5224	0.1389	0.047*
C6	0.90550 (10)	0.0208 (3)	0.22081 (11)	0.0346 (6)
H6	0.9021	−0.0943	0.2238	0.041*
C31	0.67607 (11)	0.0803 (3)	0.03315 (12)	0.0398 (7)
H31A	0.7028	0.1727	0.0405	0.048*
H31B	0.6830	0.0114	0.0013	0.048*
H31C	0.6403	0.1287	0.0223	0.048*
C13	0.99038 (12)	0.2021 (3)	0.03501 (14)	0.0402 (7)
H13	1.0239	0.2608	0.0465	0.048*
C14	0.96521 (12)	0.1774 (3)	−0.02363 (13)	0.0415 (7)
H14	0.9804	0.2110	−0.0574	0.050*
C19	1.00870 (12)	−0.3134 (3)	0.17223 (12)	0.0392 (7)
H19	1.0441	−0.3125	0.2049	0.047*
C7	0.90755 (11)	0.1127 (4)	0.26972 (12)	0.0457 (8)
H7	0.9086	0.0659	0.3069	0.055*
C26	0.80872 (12)	−0.3097 (3)	0.15813 (14)	0.0485 (8)
H26A	0.8155	−0.3860	0.1885	0.058*
H26B	0.8369	−0.2326	0.1681	0.058*
H26C	0.8092	−0.3564	0.1214	0.058*
C30	0.69077 (13)	0.1118 (4)	0.13973 (13)	0.0522 (8)
H30A	0.6570	0.1692	0.1313	0.063*
H30B	0.7210	0.1795	0.1404	0.063*
H30C	0.6967	0.0629	0.1774	0.063*
C9	0.90472 (12)	0.3345 (3)	0.21115 (14)	0.0493 (8)
H9	0.9046	0.4439	0.2078	0.059*
C32	0.63417 (11)	−0.1044 (3)	0.08664 (13)	0.0447 (7)
H32A	0.6031	−0.0367	0.0808	0.054*

H32B	0.6379	−0.1617	0.1223	0.054*
H32C	0.6286	−0.1734	0.0539	0.054*
C8	0.90746 (12)	0.2702 (4)	0.26461 (15)	0.0531 (9)
H8	0.9090	0.3344	0.2981	0.064*
C27	0.71139 (13)	−0.3732 (3)	0.13751 (15)	0.0559 (9)
H27A	0.7197	−0.4443	0.1697	0.073*
H27B	0.7150	−0.4233	0.1025	0.073*
H27C	0.6742	−0.3367	0.1313	0.073*
C28	0.75147 (15)	−0.1735 (4)	0.21238 (12)	0.0631 (10)
H28A	0.7579	−0.2547	0.2408	0.082*
H28B	0.7163	−0.1272	0.2100	0.082*
H28C	0.7801	−0.0981	0.2242	0.082*

Atomic displacement parameters (Å^2)

	U^{11}	U^{22}	U^{33}	U^{12}	U^{13}	U^{23}
O24	0.0242 (10)	0.0379 (10)	0.0297 (10)	−0.0031 (8)	0.0121 (8)	−0.0100 (8)
N1	0.0210 (11)	0.0291 (11)	0.0255 (11)	−0.0010 (9)	0.0071 (9)	0.0013 (9)
C17	0.0248 (14)	0.0241 (12)	0.0252 (12)	−0.0010 (10)	0.0090 (11)	0.0033 (10)
C4	0.0192 (13)	0.0245 (12)	0.0263 (12)	−0.0008 (10)	0.0056 (10)	−0.0018 (10)
C22	0.0277 (14)	0.0265 (13)	0.0284 (13)	−0.0027 (11)	0.0073 (11)	0.0029 (11)
C5	0.0189 (13)	0.0328 (14)	0.0305 (14)	−0.0005 (11)	0.0069 (11)	−0.0042 (11)
C2	0.0220 (14)	0.0308 (13)	0.0273 (13)	0.0005 (11)	0.0086 (11)	−0.0001 (11)
C23	0.0221 (13)	0.0325 (13)	0.0211 (12)	−0.0022 (10)	0.0086 (11)	−0.0060 (10)
C16	0.0266 (14)	0.0293 (13)	0.0344 (14)	0.0032 (11)	0.0115 (12)	0.0019 (11)
C18	0.0306 (15)	0.0293 (13)	0.0304 (14)	0.0009 (12)	0.0010 (12)	−0.0026 (11)
C11	0.0231 (13)	0.0191 (11)	0.0351 (14)	0.0032 (10)	0.0131 (11)	0.0013 (10)
C12	0.0244 (14)	0.0265 (13)	0.0466 (16)	0.0013 (11)	0.0140 (13)	−0.0003 (12)
C3	0.0252 (14)	0.0349 (14)	0.0376 (15)	0.0048 (11)	0.0094 (12)	0.0104 (12)
C15	0.0438 (18)	0.0377 (15)	0.0353 (15)	0.0112 (13)	0.0196 (13)	0.0077 (12)

C29	0.0250 (14)	0.0436 (15)	0.0287 (14)	0.0003 (12)	0.0083 (11)	−0.0091 (12)
C25	0.0327 (16)	0.0451 (16)	0.0349 (15)	−0.0043 (13)	0.0124 (13)	0.0085 (13)
C10	0.0318 (16)	0.0342 (14)	0.0442 (16)	0.0004 (12)	0.0150 (13)	−0.0085 (13)
C21	0.0368 (17)	0.0245 (13)	0.0384 (15)	−0.0019 (12)	0.0098 (13)	0.0002 (11)
C20	0.0403 (17)	0.0289 (14)	0.0481 (17)	0.0099 (13)	0.0130 (14)	0.0072 (13)
C6	0.0241 (15)	0.0455 (16)	0.0326 (15)	−0.0045 (12)	0.0052 (12)	−0.0057 (13)
C31	0.0293 (16)	0.0463 (16)	0.0430 (16)	0.0105 (13)	0.0083 (13)	0.0027 (13)
C13	0.0342 (16)	0.0282 (14)	0.067 (2)	−0.0048 (12)	0.0283 (16)	−0.0011 (14)
C14	0.0480 (19)	0.0322 (15)	0.0566 (19)	0.0093 (14)	0.0350 (16)	0.0117 (14)
C19	0.0351 (16)	0.0413 (16)	0.0362 (15)	0.0072 (13)	0.0008 (13)	0.0050 (13)
C7	0.0310 (17)	0.076 (2)	0.0293 (15)	−0.0026 (15)	0.0062 (13)	−0.0101 (15)
C26	0.0439 (19)	0.0445 (17)	0.0573 (19)	0.0025 (14)	0.0142 (15)	0.0240 (15)
C30	0.0447 (19)	0.064 (2)	0.0468 (18)	0.0129 (16)	0.0106 (15)	−0.0180 (15)
C9	0.0428 (19)	0.0450 (17)	0.065 (2)	−0.0055 (14)	0.0237 (17)	−0.0229 (16)
C32	0.0279 (16)	0.0624 (19)	0.0472 (18)	−0.0034 (14)	0.0160 (14)	−0.0041 (15)
C8	0.0370 (18)	0.069 (2)	0.056 (2)	−0.0075 (16)	0.0175 (16)	−0.0362 (18)
C27	0.0405 (19)	0.0486 (18)	0.082 (2)	−0.0063 (15)	0.0211 (18)	0.0212 (17)
C28	0.080 (3)	0.080 (2)	0.0328 (17)	0.012 (2)	0.0217 (17)	0.0173 (17)

Geometric parameters (Å, °)

O24—C23	1.428 (3)	C25—C26	1.535 (4)
O24—H24	0.82 (3)	C10—C9	1.387 (4)
N1—C3	1.465 (3)	C10—H10	0.9599
N1—C2	1.490 (3)	C21—C20	1.375 (4)
N1—C4	1.511 (3)	C21—H21	0.9599
C17—C18	1.387 (3)	C20—C19	1.380 (4)
C17—C22	1.397 (3)	C20—H20	0.9535
C17—C4	1.544 (3)	C6—C7	1.394 (4)
C4—C5	1.534 (3)	C6—H6	1.0146
C4—C11	1.543 (3)	C31—H31A	1.0362

C22—C21	1.380 (3)	C31—H31B	1.0131
C22—H22	0.9600	C31—H31C	0.9642
C5—C6	1.391 (3)	C13—C14	1.373 (4)
C5—C10	1.394 (3)	C13—H13	0.9601
C2—C3	1.485 (3)	C14—H14	1.0143
C2—C23	1.528 (3)	C19—H19	1.0087
C2—H2	0.98 (2)	C7—C8	1.384 (4)
C23—C25	1.593 (3)	C7—H7	0.9597
C23—C29	1.593 (3)	C26—H26A	0.9599
C16—C15	1.385 (3)	C26—H26B	0.9600
C16—C11	1.393 (3)	C26—H26C	0.9601
C16—H16	0.9598	C30—H30A	0.9600
C18—C19	1.384 (3)	C30—H30B	0.9599
C18—H18	1.0103	C30—H30C	0.9600
C11—C12	1.393 (3)	C9—C8	1.364 (4)
C12—C13	1.392 (4)	C9—H9	0.9601
C12—H12	1.0174	C32—H32A	0.9600
C3—H3A	0.9717	C32—H32B	0.9601
C3—H3B	1.0158	C32—H32C	0.9599
C15—C14	1.386 (4)	C8—H8	0.9602
C15—H15	0.9601	C27—H27A	0.9600
C29—C30	1.536 (4)	C27—H27B	0.9600
C29—C32	1.539 (4)	C27—H27C	0.9600
C29—C31	1.549 (4)	C28—H28A	0.9600
C25—C28	1.533 (4)	C28—H28B	0.9600
C25—C27	1.535 (4)	C28—H28C	0.9600
C23—O24—H24	107.8 (19)	C9—C10—H10	119.7
C3—N1—C2	60.32 (15)	C5—C10—H10	119.1
C3—N1—C4	116.14 (18)	C20—C21—C22	120.5 (2)

C2—N1—C4	123.15 (18)	C20—C21—H21	120.3
C18—C17—C22	117.9 (2)	C22—C21—H21	119.2
C18—C17—C4	121.9 (2)	C21—C20—C19	119.6 (2)
C22—C17—C4	119.6 (2)	C21—C20—H20	122.6
N1—C4—C5	110.88 (18)	C19—C20—H20	117.7
N1—C4—C11	106.81 (18)	C5—C6—C7	121.0 (3)
C5—C4—C11	113.27 (18)	C5—C6—H6	118.8
N1—C4—C17	108.94 (18)	C7—C6—H6	120.0
C5—C4—C17	113.47 (19)	C29—C31—H31A	107.3
C11—C4—C17	102.99 (17)	C29—C31—H31B	109.5
C21—C22—C17	120.8 (2)	H31A—C31—H31B	110.8
C21—C22—H22	119.2	C29—C31—H31C	112.9
C17—C22—H22	120.0	H31A—C31—H31C	102.6
C6—C5—C10	117.5 (2)	H31B—C31—H31C	113.4
C6—C5—C4	121.2 (2)	C14—C13—C12	121.0 (3)
C10—C5—C4	121.1 (2)	C14—C13—H13	120.0
C3—C2—N1	59.02 (14)	C12—C13—H13	119.0
C3—C2—C23	121.1 (2)	C13—C14—C15	119.0 (2)
N1—C2—C23	118.2 (2)	C13—C14—H14	125.1
C3—C2—H2	112.9 (14)	C15—C14—H14	115.7
N1—C2—H2	114.7 (14)	C20—C19—C18	120.1 (3)
C23—C2—H2	117.8 (14)	C20—C19—H19	124.5
O24—C23—C2	108.53 (18)	C18—C19—H19	115.4
O24—C23—C25	106.53 (19)	C8—C7—C6	120.2 (3)
C2—C23—C25	110.37 (19)	C8—C7—H7	120.2
O24—C23—C29	105.51 (18)	C6—C7—H7	119.6
C2—C23—C29	108.20 (19)	C25—C26—H26A	109.4
C25—C23—C29	117.31 (19)	C25—C26—H26B	110.1
C15—C16—C11	121.0 (2)	H26A—C26—H26B	109.5

C15—C16—H16	119.1	C25—C26—H26C	108.9
C11—C16—H16	119.9	H26A—C26—H26C	109.5
C19—C18—C17	121.1 (2)	H26B—C26—H26C	109.5
C19—C18—H18	117.6	C29—C30—H30A	109.4
C17—C18—H18	121.3	C29—C30—H30B	109.5
C16—C11—C12	118.1 (2)	H30A—C30—H30B	109.5
C16—C11—C4	120.7 (2)	C29—C30—H30C	109.6
C12—C11—C4	120.9 (2)	H30A—C30—H30C	109.5
C13—C12—C11	120.3 (3)	H30B—C30—H30C	109.5
C13—C12—H12	119.4	C8—C9—C10	120.8 (3)
C11—C12—H12	120.0	C8—C9—H9	119.0
N1—C3—C2	60.66 (15)	C10—C9—H9	120.2
N1—C3—H3A	118.3	C29—C32—H32A	109.2
C2—C3—H3A	112.7	C29—C32—H32B	109.4
N1—C3—H3B	118.1	H32A—C32—H32B	109.5
C2—C3—H3B	124.1	C29—C32—H32C	109.8
H3A—C3—H3B	113.4	H32A—C32—H32C	109.5
C16—C15—C14	120.4 (3)	H32B—C32—H32C	109.5
C16—C15—H15	119.8	C9—C8—C7	119.3 (3)
C14—C15—H15	119.7	C9—C8—H8	119.9
C30—C29—C32	108.3 (2)	C7—C8—H8	120.8
C30—C29—C31	105.4 (2)	C25—C27—H27A	109.5
C32—C29—C31	105.2 (2)	C25—C27—H27B	109.5
C30—C29—C23	113.8 (2)	H27A—C27—H27B	109.5
C32—C29—C23	113.1 (2)	C25—C27—H27C	109.5
C31—C29—C23	110.48 (19)	H27A—C27—H27C	109.5
C28—C25—C27	108.8 (2)	H27B—C27—H27C	109.5
C28—C25—C26	107.2 (2)	C25—C28—H28A	109.5
C27—C25—C26	104.4 (2)	C25—C28—H28B	109.5

C28—C25—C23	113.6 (2)	H28A—C28—H28B	109.5
C27—C25—C23	113.5 (2)	C25—C28—H28C	109.5
C26—C25—C23	108.6 (2)	H28A—C28—H28C	109.5
C9—C10—C5	121.2 (3)	H28B—C28—H28C	109.5
C3—N1—C4—C5	62.5 (2)	C16—C11—C12—C13	2.2 (3)
C2—N1—C4—C5	−7.8 (3)	C4—C11—C12—C13	176.2 (2)
C3—N1—C4—C11	−61.4 (2)	C4—N1—C3—C2	−114.9 (2)
C2—N1—C4—C11	−131.6 (2)	C23—C2—C3—N1	−106.4 (2)
C3—N1—C4—C17	−171.99 (18)	C11—C16—C15—C14	0.1 (4)
C2—N1—C4—C17	117.8 (2)	O24—C23—C29—C30	−167.5 (2)
C18—C17—C4—N1	−157.0 (2)	C2—C23—C29—C30	−51.5 (3)
C22—C17—C4—N1	32.4 (3)	C25—C23—C29—C30	74.1 (3)
C18—C17—C4—C5	−32.9 (3)	O24—C23—C29—C32	68.3 (2)
C22—C17—C4—C5	156.4 (2)	C2—C23—C29—C32	−175.7 (2)
C18—C17—C4—C11	89.9 (3)	C25—C23—C29—C32	−50.1 (3)
C22—C17—C4—C11	−80.8 (2)	O24—C23—C29—C31	−49.3 (2)
C18—C17—C22—C21	1.0 (3)	C2—C23—C29—C31	66.7 (2)
C4—C17—C22—C21	172.0 (2)	C25—C23—C29—C31	−167.7 (2)
N1—C4—C5—C6	85.5 (3)	O24—C23—C25—C28	−172.1 (2)
C11—C4—C5—C6	−154.4 (2)	C2—C23—C25—C28	70.3 (3)
C17—C4—C5—C6	−37.5 (3)	C29—C23—C25—C28	−54.2 (3)
N1—C4—C5—C10	−88.7 (3)	O24—C23—C25—C27	−47.0 (3)
C11—C4—C5—C10	31.3 (3)	C2—C23—C25—C27	−164.6 (2)
C17—C4—C5—C10	148.3 (2)	C29—C23—C25—C27	70.9 (3)
C4—N1—C2—C3	103.5 (2)	O24—C23—C25—C26	68.7 (3)
C3—N1—C2—C23	111.2 (2)	C2—C23—C25—C26	−49.0 (3)
C4—N1—C2—C23	−145.3 (2)	C29—C23—C25—C26	−173.5 (2)
C3—C2—C23—O24	42.3 (3)	C6—C5—C10—C9	1.1 (4)
N1—C2—C23—O24	−26.7 (3)	C4—C5—C10—C9	175.5 (2)

C3—C2—C23—C25	158.7 (2)	C17—C22—C21—C20	0.8 (4)
N1—C2—C23—C25	89.7 (2)	C22—C21—C20—C19	−1.5 (4)
C3—C2—C23—C29	−71.7 (3)	C10—C5—C6—C7	−0.5 (4)
N1—C2—C23—C29	−140.70 (19)	C4—C5—C6—C7	−175.0 (2)
C22—C17—C18—C19	−2.0 (4)	C11—C12—C13—C14	−1.1 (4)
C4—C17—C18—C19	−172.9 (2)	C12—C13—C14—C15	−0.5 (4)
C5—C4—C11—C12	38.9 (3)	C6—C7—C8—C9	0.7 (5)
C17—C4—C11—C12	−84.1 (2)		

All esds (except the esd in the dihedral angle between two l.s. planes) are estimated using the full covariance matrix. The cell esds are taken into account individually in the estimation of esds in distances, angles and torsion angles; correlations between esds in cell parameters are only used when they are defined by crystal symmetry. An approximate (isotropic) treatment of cell esds is used for estimating esds involving l.s. planes.

Crystal data for compound 6d1

Crystal data

$C_{27}H_{31}NO_2$	Monoclinic, *P*2₁/*n*
M_r = 401.53	D_x = 1.196 Mg m^{-3}
Z = 4	Mo *Kα* radiation, λ = 0.71073 Å
a = 10.1290 (2) Å	Cell parameters from 2931 reflections
b = 21.4734 (5) Å	θ = 0.9–27.5°
c = 11.2441 (3) Å	μ = 0.07 mm^{-1}
β = 114.2409 (10)°	*T* = 183 K
V = 2229.98 (9) Å^3	Prism, colourless
F(000) = 864	0.34 × 0.24 × 0.03 mm

Data collection

Bruker-Nonius KappaCCD diffractometer	3852 reflections with $I > 2\sigma(I)$
Radiation source: fine-focus sealed tube	R_{int} = 0.039
Graphite monochromator	$2\theta_{max}$ = 58.0°,
φ and ω scan	h = −13 13
5716 independent reflections	k = −29 26
9463 measured reflections	l = −15 15

Refinement

Refinement on F^2	Secondary atom site location: difference Fourier map
Least-squares matrix: full	Hydrogen site location: mixed
$R[F^2 > 2\sigma(F^2)]$ = 0.060	H atoms treated by a mixture of independent and constrained refinement
$wR(F^2)$ = 0.137	$w = 1/[\sigma^2(F_o^2) + (0.0437P)^2 + 1.0682P]$ where $P = (F_o^2 + 2F_c^2)/3$

S = 1.01	$(\Delta/\sigma)_{max}$ = 0.011
5716 reflections	$\Delta\rho_{max}$ = 0.25 e Å^{-3}
279 parameters	$\Delta\rho_{min}$ = −0.20 e Å^{-3}
0 restraints	Extinction correction: none
Primary atom site location: structure-invariant direct methods	

Refinement of F^2 against ALL reflections. The weighted R-factor wR and goodness of fit S are based on F^2, conventional R-factors R are based on F, with F set to zero for negative F^2. The threshold expression of $F^2 > 2sigma(F^2)$ is used only for calculating R-factors(gt) etc. and is not relevant to the choice of reflections for refinement. R-factors based on F^2 are statistically about twice as large as those based on F, and R-factors based on ALL data will be even larger.

Fractional atomic coordinates and isotropic or equivalent isotropic displacement parameters (Å^2)

	X	y	z	U_{iso}*/U_{eq}
N1	0.39493 (14)	0.17771 (6)	0.60222 (12)	0.0253 (3)
O25	0.57074 (13)	0.10366 (6)	0.48488 (13)	0.0334 (3)
H25	0.616 (3)	0.1270 (12)	0.557 (3)	0.064 (8)*

O11	0.67065 (14)	0.19706 (6)	0.72208 (13)	0.0386 (3)
H11	0.573 (3)	0.2035 (11)	0.668 (2)	0.061 (7)*
C4	0.39462 (16)	0.15780 (8)	0.73076 (15)	0.0249 (3)
C6	0.67213 (18)	0.15281 (8)	0.81040 (16)	0.0298 (4)
C12	0.27332 (17)	0.11127 (8)	0.71433 (15)	0.0278 (4)
C24	0.43845 (17)	0.13648 (8)	0.40819 (16)	0.0265 (4)
C18	0.38036 (17)	0.21844 (8)	0.79856 (16)	0.0272 (4)
C2	0.34621 (17)	0.13685 (8)	0.48639 (15)	0.0261 (4)
H2	0.2991	0.0962	0.4836	0.031*
C27	0.36294 (19)	0.09696 (9)	0.28152 (17)	0.0337 (4)
C5	0.54491 (17)	0.12994 (8)	0.81572 (15)	0.0260 (4)
C3	0.25577 (17)	0.19018 (9)	0.49144 (16)	0.0312 (4)
H3A	0.1687	0.1815	0.5013	0.037*

H3B	0.2531	0.2303	0.4479	0.037*
C9	0.6947 (2)	0.06445 (9)	0.99840 (17)	0.0378 (4)
H9	0.6980	0.0315	1.0596	0.045*
C26	0.47580 (19)	0.20216 (8)	0.38009 (17)	0.0324 (4)
H26A	0.5393	0.2014	0.3332	0.039*
H26B	0.3926	0.2259	0.3266	0.039*
H26C	0.5196	0.2274	0.4565	0.039*
C13	0.28552 (19)	0.04961 (8)	0.67974 (16)	0.0328 (4)
H13	0.3701	0.0335	0.6700	0.039*
C10	0.56019 (19)	0.08596 (8)	0.91192 (16)	0.0314 (4)
H10	0.4696	0.0654	0.9144	0.038*
C19	0.3596 (2)	0.27637 (8)	0.73947 (18)	0.0339 (4)
H19	0.3363	0.2799	0.6440	0.041*

C20	0.3461 (2)	0.32944 (9)	0.80463 (19)	0.0390 (4)
H20	0.3305	0.3694	0.7528	0.047*
C17	0.14797 (19)	0.12802 (10)	0.72896 (17)	0.0368 (4)
H17	0.1356	0.1736	0.7498	0.044*
C22	0.3766 (2)	0.26822 (9)	0.99038 (18)	0.0370 (4)
H22	0.3943	0.2663	1.0872	0.044*
C23	0.3905 (2)	0.21536 (9)	0.92582 (17)	0.0346 (4)
H23	0.4081	0.1761	0.9704	0.041*
C21	0.35381 (19)	0.32561 (9)	0.92951 (18)	0.0364 (4)
H21	0.3376	0.3646	0.9735	0.044*
C7	0.80758 (19)	0.13136 (9)	0.89766 (18)	0.0380 (4)
H7	0.8957	0.1512	0.8921	0.046*
C28	0.3393 (2)	0.02969 (10)	0.3154 (2)	0.0473 (5)

H28A	0.2929	0.0061	0.2365	0.057*
H28B	0.2792	0.0298	0.3629	0.057*
H28C	0.4311	0.0111	0.3680	0.057*
C29	0.4585 (2)	0.09445 (10)	0.20555 (19)	0.0427 (5)
H29A	0.4119	0.0701	0.1278	0.051*
H29B	0.5498	0.0759	0.2594	0.051*
H29C	0.4741	0.1360	0.1824	0.051*
C14	0.1767 (2)	0.00666 (10)	0.65940 (18)	0.0430 (5)
H14	0.1875	−0.0350	0.6337	0.052*
C8	0.8184 (2)	0.08744 (10)	0.99092 (19)	0.0411 (5)
H8	0.9183	0.0738	1.0528	0.049*
C15	0.0540 (2)	0.02368 (11)	0.6753 (2)	0.0494 (6)
H15	−0.0208	−0.0064	0.6625	0.059*

C16	0.0398 (2)	0.08378 (12)	0.7103 (2)	0.0491 (6)
H16	−0.0455	0.0951	0.7224	0.059*
C30	0.2165 (2)	0.12577 (12)	0.1938 (2)	0.0553 (6)
H30A	0.1714	0.1010	0.1165	0.066*
H30B	0.2312	0.1673	0.1701	0.066*
H30C	0.1551	0.1270	0.2400	0.066*

Atomic displacement parameters (Å^2)

	U^{11}	U^{22}	U^{33}	U^{12}	U^{13}	U^{23}
N1	0.0277 (7)	0.0260 (7)	0.0225 (6)	0.0021 (6)	0.0107 (5)	0.0016 (5)
O25	0.0314 (6)	0.0355 (7)	0.0326 (7)	0.0066 (5)	0.0123 (5)	0.0048 (6)

O11	0.0334 (7)	0.0405 (8)	0.0398 (7)	−0.0111 (6)	0.0127 (6)	0.0005 (6)
C4	0.0269 (8)	0.0251 (8)	0.0225 (7)	0.0004 (6)	0.0099 (6)	0.0014 (6)
C6	0.0316 (8)	0.0280 (9)	0.0282 (8)	−0.0028 (7)	0.0107 (7)	−0.0069 (7)
C12	0.0292 (8)	0.0320 (9)	0.0206 (7)	−0.0015 (7)	0.0085 (7)	0.0030 (7)
C24	0.0279 (8)	0.0258 (9)	0.0260 (8)	0.0029 (7)	0.0112 (7)	0.0025 (7)
C18	0.0278 (8)	0.0275 (9)	0.0264 (8)	0.0019 (7)	0.0114 (7)	−0.0013 (7)
C2	0.0279 (8)	0.0268 (9)	0.0222 (8)	−0.0035 (6)	0.0089 (7)	−0.0028 (6)
C27	0.0380 (9)	0.0372 (10)	0.0278 (9)	−0.0014 (8)	0.0154 (8)	−0.0041 (8)
C5	0.0279 (8)	0.0245 (8)	0.0233 (8)	0.0004 (6)	0.0082 (7)	−0.0044 (6)
C3	0.0268 (8)	0.0398 (10)	0.0270 (8)	0.0049 (7)	0.0111 (7)	0.0042 (7)
C9	0.0416 (10)	0.0363 (10)	0.0267 (9)	0.0048 (8)	0.0051 (8)	−0.0001 (8)
C26	0.0356 (9)	0.0311 (10)	0.0330 (9)	0.0005 (7)	0.0166 (8)	0.0041 (7)
C13	0.0365 (9)	0.0303 (10)	0.0275 (8)	−0.0036 (7)	0.0088 (7)	0.0050 (7)

C10	0.0357 (9)	0.0295 (9)	0.0254 (8)	0.0014 (7)	0.0089 (7)	−0.0009 (7)
C19	0.0443 (10)	0.0280 (9)	0.0311 (9)	0.0050 (8)	0.0172 (8)	0.0012 (7)
C20	0.0501 (11)	0.0281 (10)	0.0387 (10)	0.0065 (8)	0.0182 (9)	0.0009 (8)
C17	0.0317 (9)	0.0484 (12)	0.0307 (9)	−0.0013 (8)	0.0132 (8)	0.0042 (8)
C22	0.0435 (10)	0.0396 (11)	0.0302 (9)	0.0020 (8)	0.0174 (8)	−0.0046 (8)
C23	0.0454 (10)	0.0313 (10)	0.0293 (9)	0.0037 (8)	0.0176 (8)	0.0027 (7)
C21	0.0364 (9)	0.0346 (10)	0.0389 (10)	0.0056 (8)	0.0162 (8)	−0.0087 (8)
C7	0.0278 (8)	0.0422 (11)	0.0395 (10)	−0.0039 (8)	0.0093 (8)	−0.0120 (9)
C28	0.0671 (14)	0.0401 (12)	0.0458 (11)	−0.0179 (10)	0.0343 (11)	−0.0165 (9)
C29	0.0605 (13)	0.0410 (11)	0.0347 (10)	0.0020 (9)	0.0277 (9)	0.0004 (8)
C14	0.0516 (12)	0.0354 (11)	0.0328 (10)	−0.0132 (9)	0.0082 (9)	0.0045 (8)
C8	0.0339 (9)	0.0428 (11)	0.0345 (10)	0.0082 (8)	0.0017 (8)	−0.0073 (9)
C15	0.0431 (11)	0.0607 (15)	0.0376 (11)	−0.0225 (10)	0.0096 (9)	0.0073 (10)

C16	0.0324 (10)	0.0785 (17)	0.0381 (11)	−0.0083 (10)	0.0161 (9)	0.0076 (11)
C30	0.0426 (11)	0.0734 (17)	0.0361 (11)	0.0019 (11)	0.0022 (9)	−0.0144 (11)

Geometric parameters (Å, °)

N1—C3	1.472 (2)	C26—H26C	0.9569
N1—C2	1.477 (2)	C13—C14	1.382 (3)
N1—C4	1.508 (2)	C13—H13	0.9708
O25—C24	1.4446 (19)	C10—H10	1.0289
O25—H25	0.90 (3)	C19—C20	1.392 (3)
O11—C6	1.370 (2)	C19—H19	1.0032
O11—H11	0.94 (2)	C20—C21	1.376 (3)
C4—C12	1.535 (2)	C20—H20	1.0131
C4—C18	1.545 (2)	C17—C16	1.399 (3)
C4—C5	1.547 (2)	C17—H17	1.0264

C6—C7	1.397 (2)	C22—C21	1.382 (3)
C6—C5	1.403 (2)	C22—C23	1.385 (3)
C12—C17	1.393 (2)	C22—H22	1.0286
C12—C13	1.400 (2)	C23—H23	0.9599
C24—C2	1.523 (2)	C21—H21	1.0197
C24—C26	1.526 (2)	C7—C8	1.381 (3)
C24—C27	1.562 (2)	C7—H7	1.0138
C18—C19	1.385 (2)	C28—H28A	0.9601
C18—C23	1.393 (2)	C28—H28B	0.9599
C2—C3	1.482 (2)	C28—H28C	0.9600
C2—H2	0.9881	C29—H29A	0.9600
C27—C29	1.532 (3)	C29—H29B	0.9601
C27—C30	1.535 (3)	C29—H29C	0.9602

C27—C28	1.538 (3)	C14—C15	1.376 (3)
C5—C10	1.396 (2)	C14—H14	0.9602
C3—H3A	0.9520	C8—H8	1.0084
C3—H3B	0.9851	C15—C16	1.374 (3)
C9—C8	1.380 (3)	C15—H15	0.9600
C9—C10	1.389 (2)	C16—H16	0.9600
C9—H9	0.9790	C30—H30A	0.9600
C26—H26A	0.9848	C30—H30B	0.9601
C26—H26B	0.9570	C30—H30C	0.9601
C3—N1—C2	60.33 (11)	C14—C13—H13	115.1
C3—N1—C4	118.96 (13)	C12—C13—H13	123.4
C2—N1—C4	123.03 (13)	C9—C10—C5	122.17 (17)
C24—O25—H25	105.8 (16)	C9—C10—H10	117.9

C6—O11—H11	105.6 (15)	C5—C10—H10	119.7
N1—C4—C12	112.69 (12)	C18—C19—C20	120.77 (17)
N1—C4—C18	105.74 (13)	C18—C19—H19	120.3
C12—C4—C18	112.06 (13)	C20—C19—H19	118.0
N1—C4—C5	108.22 (13)	C21—C20—C19	120.75 (18)
C12—C4—C5	111.02 (13)	C21—C20—H20	124.6
C18—C4—C5	106.78 (12)	C19—C20—H20	114.7
O11—C6—C7	116.94 (16)	C12—C17—C16	120.2 (2)
O11—C6—C5	122.46 (15)	C12—C17—H17	118.4
C7—C6—C5	120.60 (17)	C16—C17—H17	121.4
C17—C12—C13	117.77 (16)	C21—C22—C23	120.21 (17)
C17—C12—C4	122.76 (16)	C21—C22—H22	118.8
C13—C12—C4	119.44 (15)	C23—C22—H22	120.6

O25—C24—C2	107.24 (13)	C22—C23—C18	121.24 (17)
O25—C24—C26	109.05 (13)	C22—C23—H23	118.9
C2—C24—C26	112.18 (14)	C18—C23—H23	119.9
O25—C24—C27	105.43 (13)	C20—C21—C22	119.11 (17)
C2—C24—C27	110.02 (13)	C20—C21—H21	119.5
C26—C24—C27	112.55 (14)	C22—C21—H21	121.3
C19—C18—C23	117.89 (16)	C8—C7—C6	120.48 (18)
C19—C18—C4	123.15 (15)	C8—C7—H7	122.4
C23—C18—C4	118.95 (15)	C6—C7—H7	117.0
N1—C2—C3	59.68 (10)	C27—C28—H28A	109.5
N1—C2—C24	116.74 (13)	C27—C28—H28B	109.5
C3—C2—C24	124.15 (15)	H28A—C28—H28B	109.5
N1—C2—H2	122.3	C27—C28—H28C	109.5

C3—C2—H2	112.6	H28A—C28—H28C	109.5
C24—C2—H2	112.3	H28B—C28—H28C	109.5
C29—C27—C30	108.74 (17)	C27—C29—H29A	109.9
C29—C27—C28	107.66 (16)	C27—C29—H29B	109.2
C30—C27—C28	109.28 (17)	H29A—C29—H29B	109.5
C29—C27—C24	110.04 (15)	C27—C29—H29C	109.3
C30—C27—C24	110.44 (15)	H29A—C29—H29C	109.5
C28—C27—C24	110.63 (15)	H29B—C29—H29C	109.5
C10—C5—C6	117.27 (15)	C15—C14—C13	120.2 (2)
C10—C5—C4	120.54 (15)	C15—C14—H14	120.5
C6—C5—C4	121.76 (15)	C13—C14—H14	119.3
N1—C3—C2	59.99 (10)	C7—C8—C9	119.97 (17)
N1—C3—H3A	118.5	C7—C8—H8	117.8

C2—C3—H3A	118.0	C9—C8—H8	122.2
N1—C3—H3B	113.6	C16—C15—C14	119.55 (19)
C2—C3—H3B	123.2	C16—C15—H15	120.4
H3A—C3—H3B	113.0	C14—C15—H15	120.1
C8—C9—C10	119.50 (18)	C15—C16—C17	120.9 (2)
C8—C9—H9	122.2	C15—C16—H16	119.0
C10—C9—H9	118.2	C17—C16—H16	120.1
C24—C26—H26A	111.6	C27—C30—H30A	109.5
C24—C26—H26B	113.1	C27—C30—H30B	109.5
H26A—C26—H26B	105.8	H30A—C30—H30B	109.5
C24—C26—H26C	113.6	C27—C30—H30C	109.5
H26A—C26—H26C	109.0	H30A—C30—H30C	109.5
H26B—C26—H26C	103.1	H30B—C30—H30C	109.5

C14—C13—C12	121.45 (18)		
C3—N1—C4—C12	40.8 (2)	O11—C6—C5—C10	−178.53 (16)
C2—N1—C4—C12	−30.8 (2)	C7—C6—C5—C10	0.7 (2)
C3—N1—C4—C18	−81.93 (16)	O11—C6—C5—C4	−6.0 (2)
C2—N1—C4—C18	−153.57 (13)	C7—C6—C5—C4	173.25 (15)
C3—N1—C4—C5	163.95 (14)	N1—C4—C5—C10	−153.50 (15)
C2—N1—C4—C5	92.32 (16)	C12—C4—C5—C10	−29.3 (2)
N1—C4—C12—C17	−104.32 (18)	C18—C4—C5—C10	93.07 (18)
C18—C4—C12—C17	14.8 (2)	N1—C4—C5—C6	34.2 (2)
C5—C4—C12—C17	134.11 (16)	C12—C4—C5—C6	158.37 (15)
N1—C4—C12—C13	73.89 (18)	C18—C4—C5—C6	−79.21 (18)
C18—C4—C12—C13	−166.99 (14)	C4—N1—C3—C2	−113.69 (15)
C5—C4—C12—C13	−47.69 (19)	C24—C2—C3—N1	−103.49 (16)

N1—C4—C18—C19	5.1 (2)	C17—C12—C13—C14	0.6 (2)
C12—C4—C18—C19	−118.06 (17)	C4—C12—C13—C14	−177.70 (15)
C5—C4—C18—C19	120.17 (17)	C8—C9—C10—C5	0.7 (3)
N1—C4—C18—C23	−174.11 (14)	C6—C5—C10—C9	−1.0 (3)
C12—C4—C18—C23	62.76 (19)	C4—C5—C10—C9	−173.59 (16)
C5—C4—C18—C23	−59.00 (19)	C23—C18—C19—C20	−1.4 (3)
C4—N1—C2—C3	107.11 (16)	C4—C18—C19—C20	179.40 (17)
C3—N1—C2—C24	115.69 (17)	C18—C19—C20—C21	0.3 (3)
C4—N1—C2—C24	−137.20 (15)	C13—C12—C17—C16	0.5 (2)
O25—C24—C2—N1	72.11 (17)	C4—C12—C17—C16	178.77 (16)
C26—C24—C2—N1	−47.60 (19)	C21—C22—C23—C18	−0.8 (3)
C27—C24—C2—N1	−173.71 (13)	C19—C18—C23—C22	1.7 (3)
O25—C24—C2—C3	142.15 (15)	C4—C18—C23—C22	−179.13 (16)

C26—C24—C2—C3	22.5 (2)	C19—C20—C21—C22	0.6 (3)
C27—C24—C2—C3	−103.66 (18)	C23—C22—C21—C20	−0.4 (3)
O25—C24—C27—C29	−62.45 (18)	O11—C6—C7—C8	179.05 (17)
C2—C24—C27—C29	−177.77 (15)	C5—C6—C7—C8	−0.3 (3)
C26—C24—C27—C29	56.32 (19)	C12—C13—C14—C15	−1.2 (3)
O25—C24—C27—C30	177.50 (16)	C6—C7—C8—C9	0.0 (3)
C2—C24—C27—C30	62.2 (2)	C10—C9—C8—C7	−0.2 (3)
C26—C24—C27—C30	−63.7 (2)	C13—C14—C15—C16	0.6 (3)
O25—C24—C27—C28	56.38 (18)	C14—C15—C16—C17	0.5 (3)
C2—C24—C27—C28	−58.94 (19)	C12—C17—C16—C15	−1.1 (3)
C26—C24—C27—C28	175.15 (15)		

All esds (except the esd in the dihedral angle between two l.s. planes) are estimated using the full covariance matrix. The cell esds are taken into account individually in the estimation of esds in distances, angles and torsion angles; correlations between esds in cell parameters are only used when they are defined by crystal symmetry.

An approximate (isotropic) treatment of cell esds is used for estimating esds involving l.s. planes.
